# Supplementary material for: Suppression of ATM kinase signaling accelerates cellular senescence
Source: Stem Cell Reports. 2026 Jun 11;21(7):102956. doi: 10.1016/j.stemcr.2026.102956 (PMC13385434; doi:10.1016/j.stemcr.2026.102956)
Supplement: Document S1. Figures S1–S11 [file mmc1.pdf]

Stem Cell Reports, Volume *21*

Supplemental Information

Suppression of ATM kinase signaling accelerates cellular senescence

Kei-ichi Ishikawa, Takahiro Shiga, Takumi Hirose, Naoko Kuzumaki, Sakura Miyoshi, Akihiro Yamaguchi, Hidetaka Tamune, Avijite Kumer Sarkar, Kento Nakai, Kazuyoshi Baba, Shigeo Okabe, Nobutaka Hattori, Hideyuki Okano, and Wado Akamatsu

Figure S1.

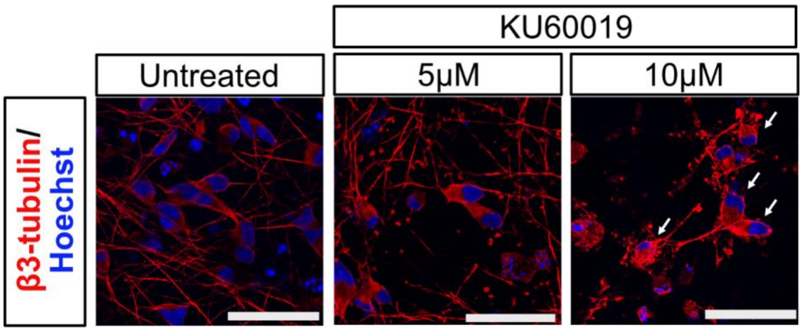

Figure S1. High-dose KU60019 treatment induces neurotoxic morphological changes, related to Figure 1.

Representative images of β3-tubulin immunostaining in 201B7 hiPSC-derived dopaminergic neurons treated with or without KU60019. Neurons treated with 10 μM KU60019 showed axonal fragmentation at day 10 (arrows). Scale bar, 50 μm.

Figure S2.

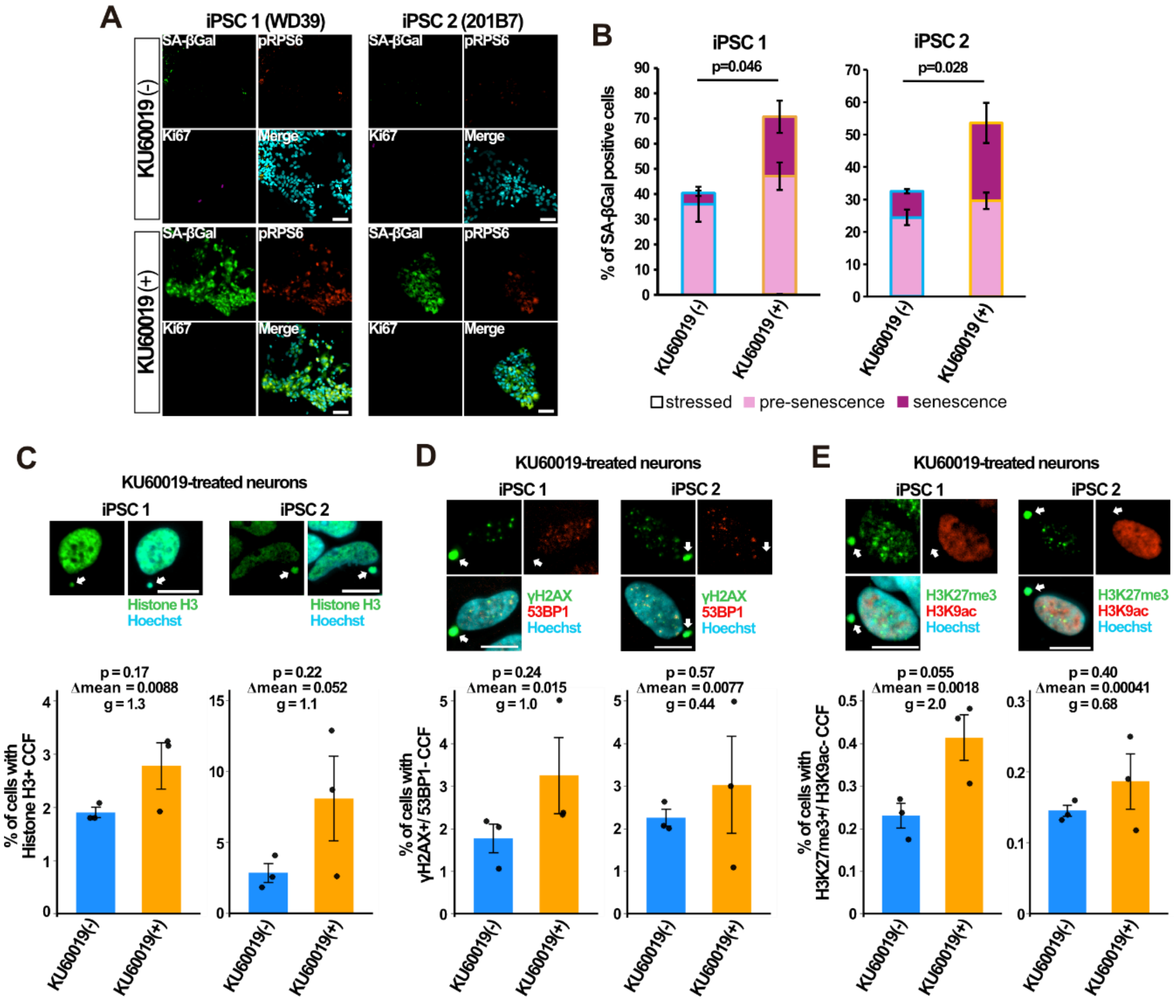

Figure S2. KU60019-treated hiPSC-derived neurons exhibit senescence-associated phenotypes, related to Figure 2.

(A) Representative images of SA-βGal, pRPS6, and Ki67 staining in untreated and KU60019-treated neurons after 14 days of differentiation. Scale bar, 100 μm.

(B) Percentage of senescent (SA-βGal$^+$, pRPS6$^+$, Ki67$^-$), pre-senescent (SA-βGal$^+$, pRPS6$^-$), and stressed (SA-βGal$^+$, pRPS6$^+$, Ki67$^+$) cells. n = 4 independent experiments.

(C) Representative images of Histone H3 and Hoechst 33258 staining in KU60019-treated neurons after 14 days of differentiation (upper panels). Scale bar, 10 μm. Lower panels show the percentage of cells with histone H3$^+$ cytoplasmic chromatin fragments (CCFs). CCFs were defined as Hoechst$^+$ extranuclear DNA structures. n = 3 independent experiments.

(D) Representative images of γH2AX, 53BP1, and Hoechst 33258 staining in KU60019-treated neurons (upper panels). Scale bar, 10 μm. Lower panels show the percentage of cells with γH2AX$^+$, 53BP1$^-$ CCFs. n = 3 independent experiments.

(E) Representative images of H3K27me3, H3K9ac, and Hoechst 33258 staining in KU60019-treated neurons (upper panels). Scale bar, 10 µm. Lower panels show the percentage of cells with $H3K27me3^{+}$, $H3K9ac^{-}$ CCFs. n = 3 independent experiments.

Data are shown as mean ± SEM. *P* values, mean differences (Δmean), and effect sizes (Hedges' g) are indicated. *P* values were calculated using Welch's t-test.

Figure S3.

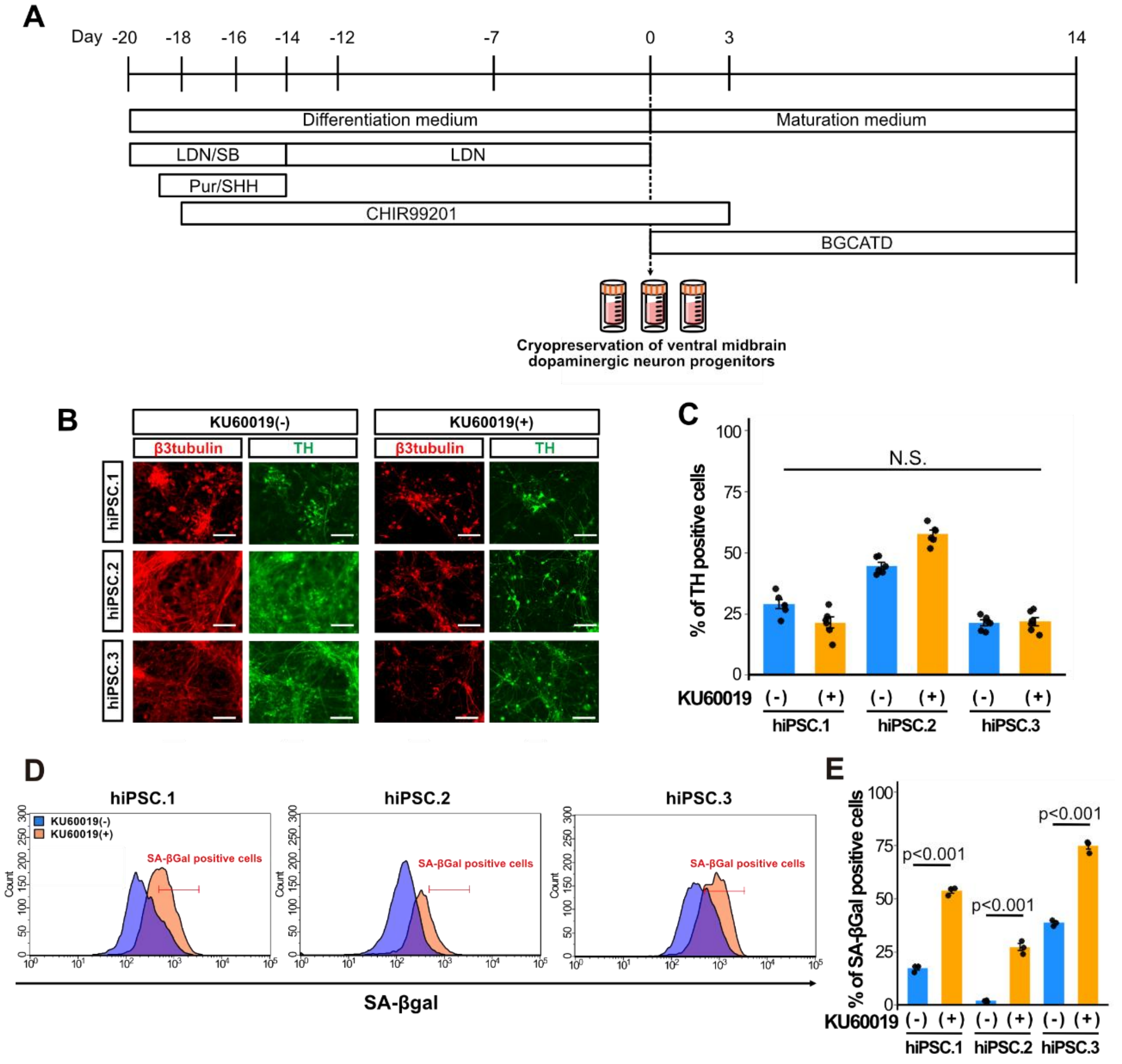

Figure S3. KU60019 induces senescence-associated phenotypes in dopaminergic neurons generated using the floor plate method, related to Figure 2.

(A) Schematic overview of the differentiation schedule for the floor plate method. LDN, LDN-193189; SB, SB431542; Pur, purmorphamine; SHH, sonic hedgehog; B, BDNF; G, GDNF; C, dibutyryl cAMP; A, ascorbic acid; T, TGF-β3; D, DAPT.

(B) Representative images of β3-tubulin and TH immunostaining in untreated and KU60019-treated hiPSC-derived dopaminergic neurons after 14 days of differentiation. Scale bar, 100 μm.

(C) Percentage of TH^+ dopaminergic neurons. n = 6 independent experiments.

(D) Representative flow cytometry histograms of SA-βGal^+ cells in untreated and KU60019-treated dopaminergic neurons after 14 days of differentiation.

(E) Percentage of SA-βGal^+ cells. n = 3 independent experiments.

Data are shown as mean ± SEM. *P* values were calculated using two-way ANOVA (condition × cell line), followed by Holm-adjusted post hoc comparisons between untreated and KU60019-treated groups within each cell line where applicable. N.S., not significant.

Figure S4.

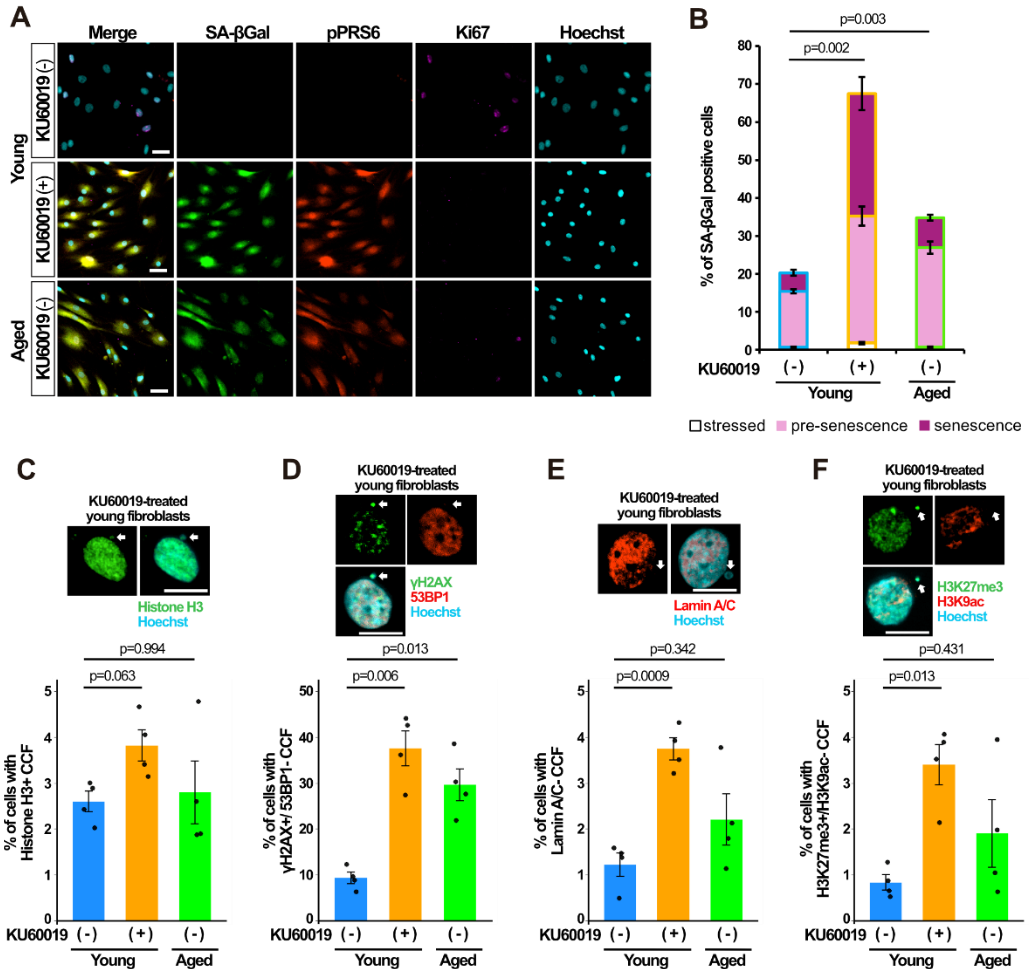

Figure S4. KU60019-treated fibroblasts exhibit senescence-associated phenotypes, related to Figure 3.

(A) Representative images of SA-βGal staining and immunostaining for pRPS6 and Ki67 in untreated young, KU60019-treated young, and aged fibroblasts. Scale bar, 100 μm.

(B) Percentage of senescent (SA-βGal$^+$, pRPS6$^+$, Ki67$^-$), pre-senescent (SA-βGal$^+$, pRPS6$^-$), and stressed (SA-βGal$^+$, pRPS6$^+$, Ki67$^+$) cells. n = 4 independent experiments.

(C) Representative images of histone H3 and Hoechst 33258 staining in KU60019-treated young fibroblasts (upper panels). Scale bar, 10 μm. Lower panel shows the percentage of cells with histone H3$^+$ cytoplasmic chromatin fragments (CCFs). CCFs were defined as Hoechst$^+$ extranuclear DNA structures. n = 3 independent experiments.

(D) Representative images of γH2AX, 53BP1, and Hoechst 33258 staining in KU60019-treated young fibroblasts (upper panels). Scale bar, 10 μm. Lower panel shows the percentage of cells with γH2AX$^+$, 53BP1$^-$ CCFs. n = 3 independent experiments.

(E) Representative images of lamin A/C and Hoechst 33258 staining in KU60019-treated young fibroblasts (upper panels). Scale bar, 10 μm. Lower panel shows the percentage of cells with lamin A/C^{-} CCFs. n = 3 independent experiments.

(F) Representative images of H3K27me3, H3K9ac, and Hoechst 33258 staining in KU60019-treated young fibroblasts (upper panels). Scale bar, 10 μm. Lower panel shows the percentage of cells with H3K27me3^{+}, H3K9ac^{-} CCFs. n = 3 independent experiments.

Data are shown as mean ± SEM. *P* values were calculated using Welch's ANOVA followed by Holm-adjusted Games–Howell post hoc comparisons.

Figure S5.

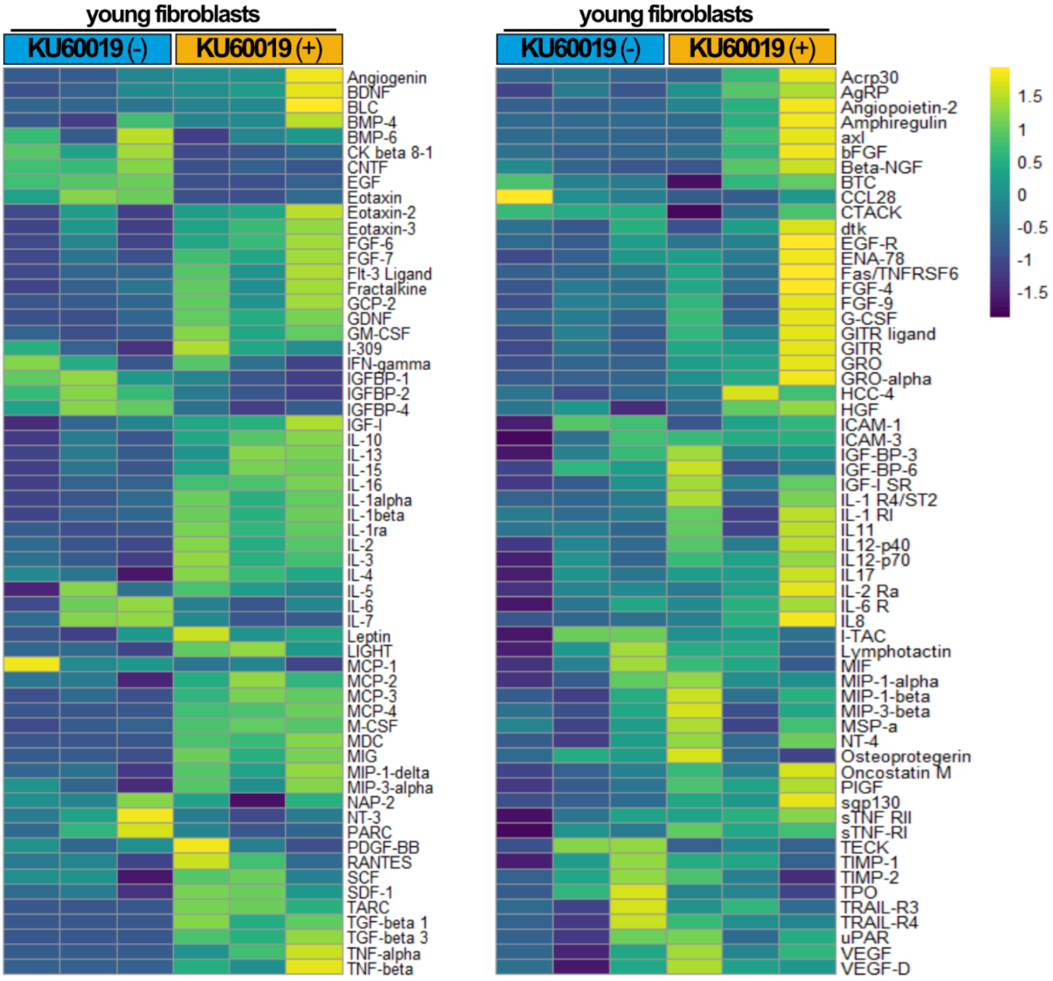

Figure S5. KU60019 treatment induces SASP factor secretion in young fibroblasts, related to Figure 3.

Heatmap of senescence-associated secretory phenotype (SASP) factors detected using a cytokine array in conditioned media from untreated and KU60019-treated young fibroblasts. Each column represents an independent experiment. n = 3 independent experiments per condition. Values represent normalized signal intensities for each factor relative to the untreated condition.

Figure S6.

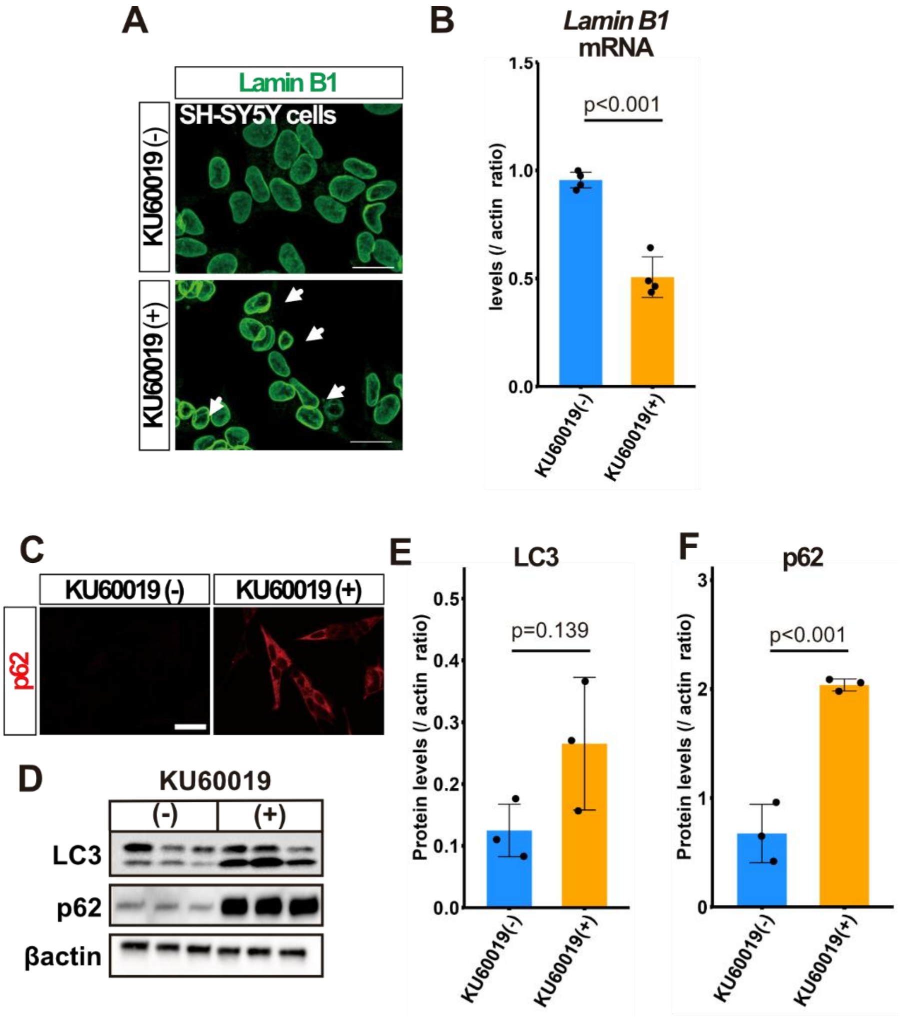

Figure S6. KU60019-treated SH-SY5Y cells exhibit senescence-associated phenotypes, related to Figure 3.

(**A**) Representative images of lamin B1 immunostaining in untreated and KU60019-treated SH-SY5Y cells. Scale bar, 10 μm.

(**B**) Lamin B1 mRNA expression levels. n = 4 independent experiments.

(**C**) Representative images of p62 immunostaining in untreated and KU60019-treated SH-SY5Y cells. Scale bar, 10 μm.

(**D**) Immunoblot analysis of LC3 and p62 protein levels in untreated and KU60019-treated SH-SY5Y cells.

(**E, F**) Quantification of the immunoblot results shown in (**D**). n = 3 independent experiments. Data are shown as mean ± SEM. *P* values were calculated using Welch's t-test.

Figure S7.

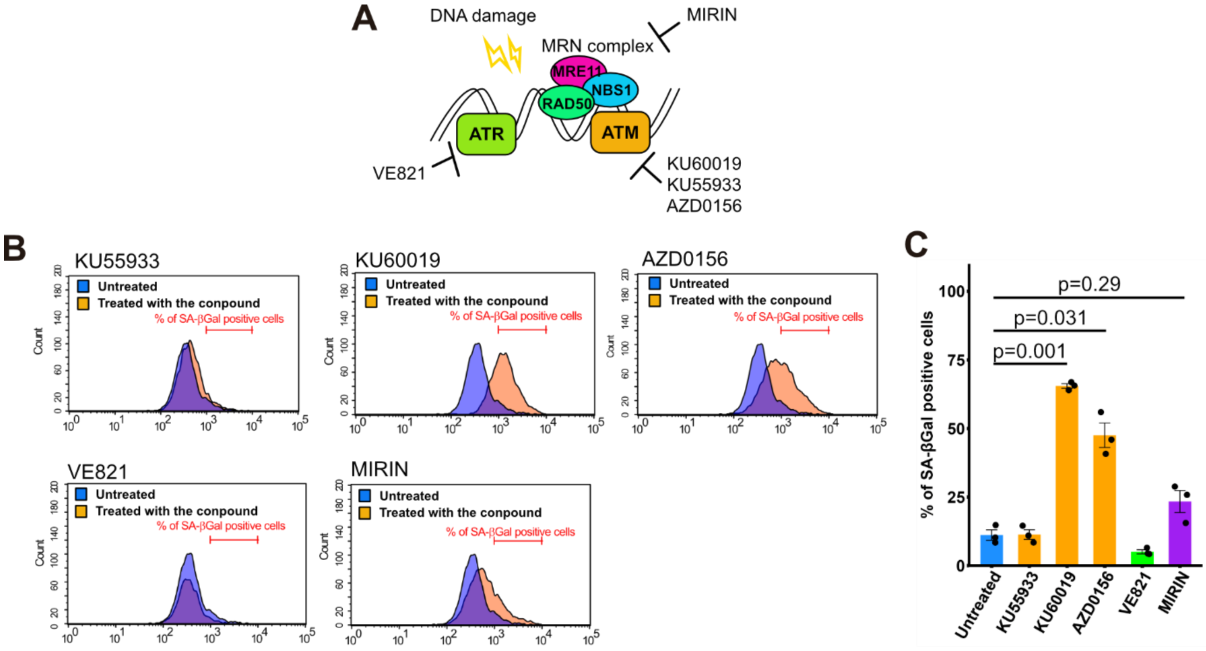

Figure S7. Screening of DNA repair inhibitors for their ability to accelerate senescence-associated phenotypes, related to Figure 3.

(A) Schematic illustration of inhibitors targeting factors involved in ATM-mediated DNA repair.

(B) Histograms showing the distribution of SA-βGal staining intensity in young fibroblasts treated with the indicated inhibitors. Blue indicates untreated cells, and orange indicates inhibitor-treated cells.

(C) Percentage of SA-βGal^{+} cells. Blue bars represent untreated cells, orange bars represent ATM inhibitor–treated cells, green bars represent ATR inhibitor–treated cells, and purple bars represent MRN complex inhibitor–treated cells. n = 3 independent experiments.

Data are shown as mean ± SEM. *P* values were calculated using Welch's ANOVA followed by Holm-adjusted Games–Howell post hoc comparisons.

Figure S8.

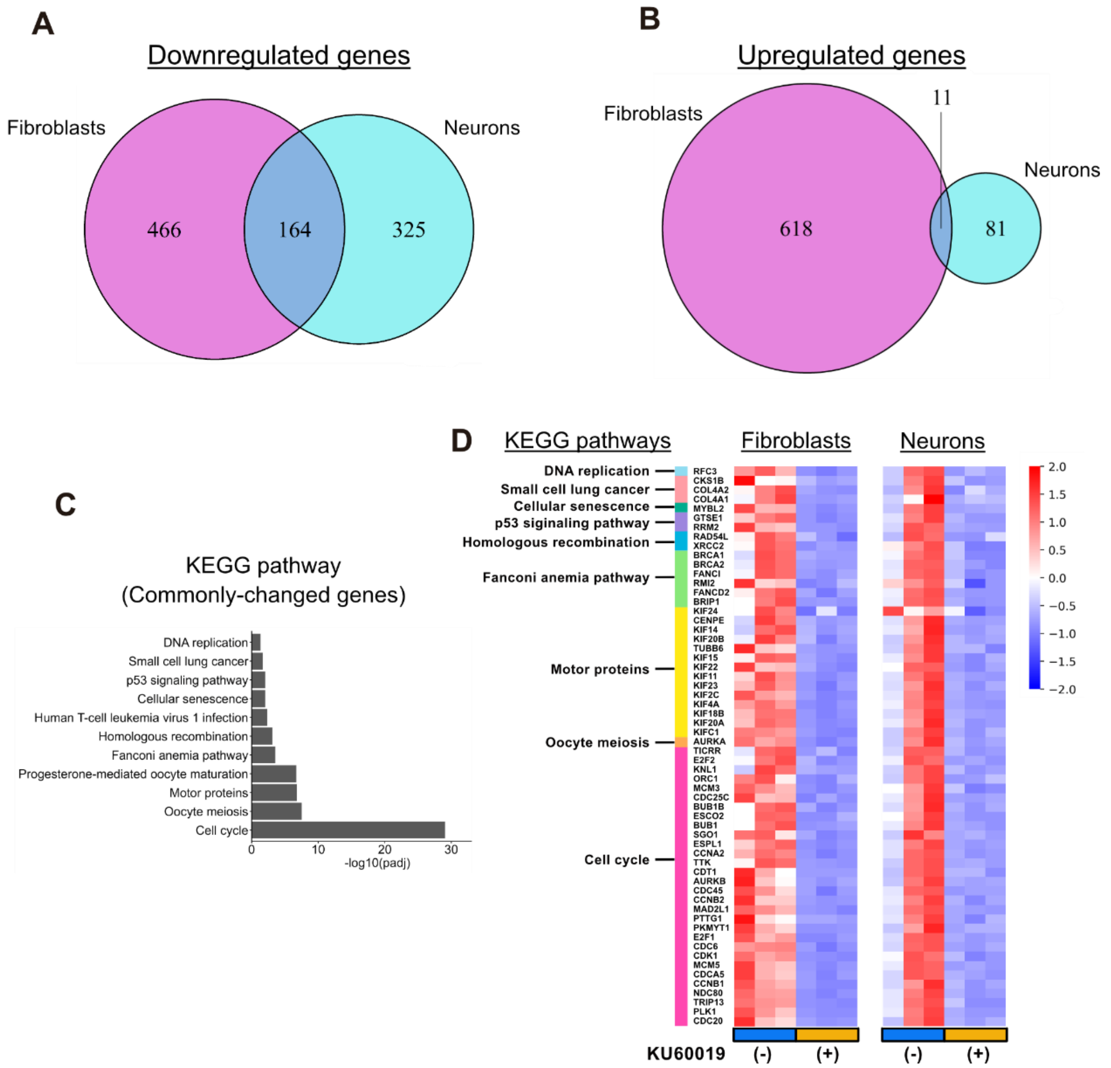

Figure S8. Transcriptomic analysis identifies genes commonly altered by KU60019 in neurons and fibroblasts, related to Figure 4.

(A, B) Venn diagrams showing genes commonly regulated by KU60019 treatment in hiPSC-derived neurons and young fibroblasts. A total of 164 genes were commonly downregulated (A), and 9 genes were commonly upregulated (B).

(C) KEGG pathway enrichment analysis of genes commonly downregulated following KU60019 treatment in hiPSC-derived dopaminergic neurons and young fibroblasts.

(D) Heatmaps showing the expression of genes associated with pathways enriched in the KEGG analysis in hiPSC-derived dopaminergic neurons and young fibroblasts.

Figure S9.

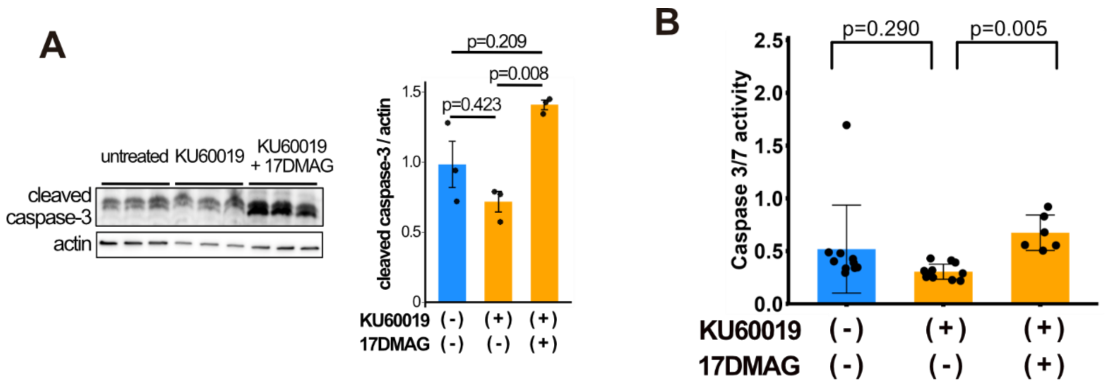

Figure S9. 17DMAG induces apoptosis in KU60019-treated cells, related to Figure 5.

(A) Immunoblot analysis of cleaved caspase-3 protein levels in untreated, KU60019-treated, and KU60019 + 17DMAG–treated neurons. n = 3 independent experiments.

(B) Caspase-3/7 activity in untreated, KU60019-treated, and KU60019 + 17DMAG–treated young fibroblasts. n = 6–12 independent experiments.

Data are shown as mean ± SEM. *P* values were calculated using Welch's ANOVA followed by Holm-adjusted Games–Howell post hoc comparisons.

Figure S10.

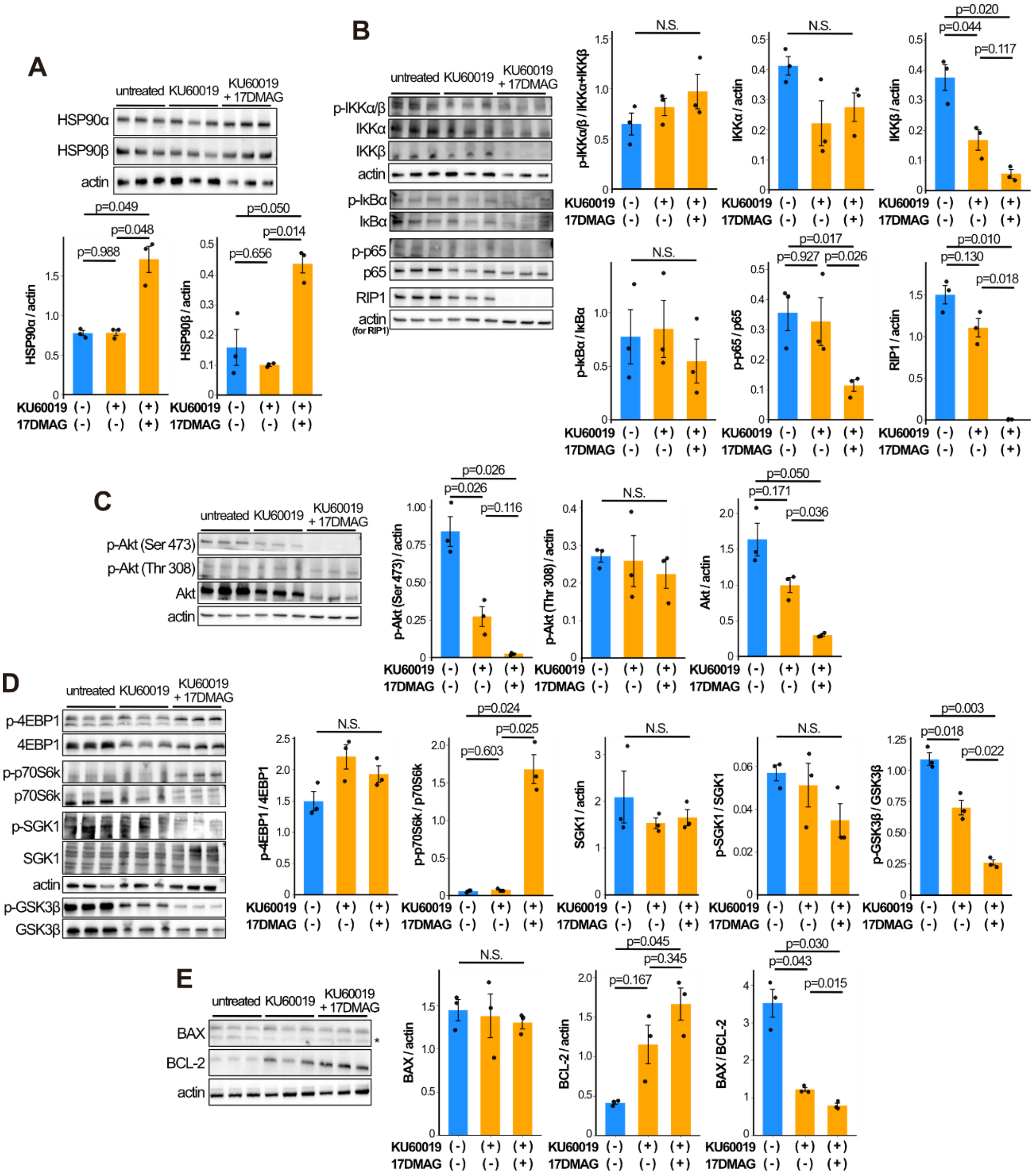

Figure S10. Reproducibility of KU60019-induced signaling alterations across independent hiPSC-derived neuronal lines, related to Figure 6.

(A) Immunoblot analysis of HSP90 protein levels in untreated, KU60019-treated, and KU60019 + 17DMAG–treated hiPSC-derived neurons from an independent neuronal line. n = 3 independent experiments.

(B) Immunoblot analysis of NF-κB signaling components and their phosphorylated forms in untreated, KU60019-treated, and KU60019 + 17DMAG–treated neurons. n = 3 independent experiments.
(C) Immunoblot analysis of Akt and phosphorylated Akt protein levels in untreated, KU60019-treated, and KU60019 + 17DMAG–treated neurons. n = 3 independent experiments.
(D) Immunoblot analysis of Akt-mTOR pathway components and their phosphorylated forms in untreated, KU60019-treated, and KU60019 + 17DMAG–treated neurons. n = 3 independent experiments.
(E) Immunoblot analysis of BAX and BCL-2 protein levels in untreated, KU60019-treated, and KU60019 + 17DMAG–treated neurons. n = 3 independent experiments.
The same actin blot is shown for RIP1 in panel (B) and for the proteins shown in panel (C) because these proteins were detected from the same membrane using the same set of lysates. Data are shown as mean ± SEM. *P* values were calculated using Welch's ANOVA followed by Holm-adjusted Games–Howell post hoc comparisons. N.S., not significant.

Figure S11.

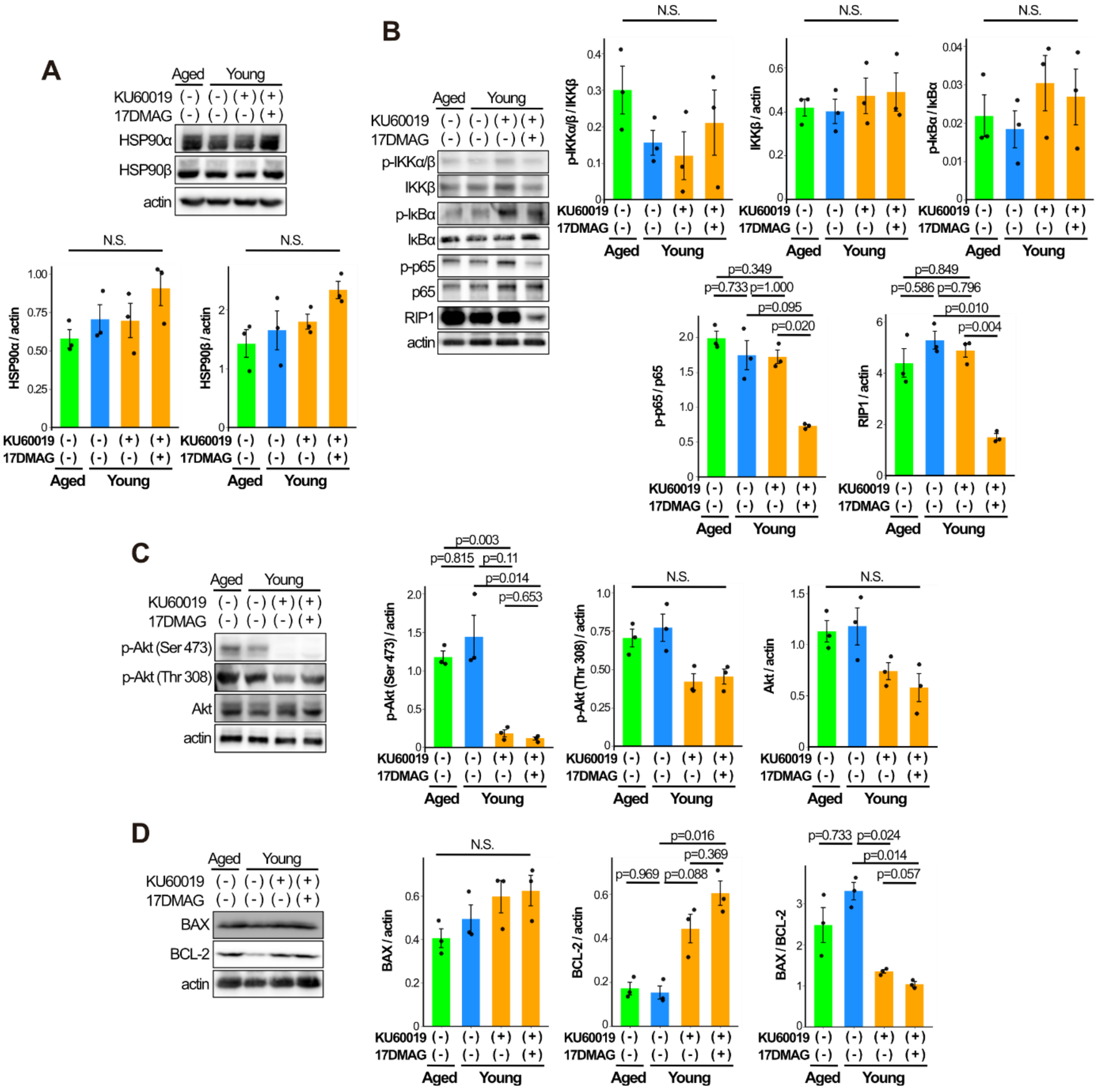

Figure S11. Reproducibility of KU60019-induced signaling alterations in fibroblasts, related to Figure 6.

(A) Immunoblot analysis of HSP90 protein levels in aged fibroblasts and in untreated, KU60019-treated, and KU60019 + 17DMAG–treated young fibroblasts. n = 3 independent experiments.

(B) Immunoblot analysis of NF-κB signaling components and their phosphorylated forms in aged fibroblasts and in untreated, KU60019-treated, and KU60019 + 17DMAG–treated young fibroblasts. n = 3 independent experiments.

(C) Immunoblot analysis of Akt and phosphorylated Akt in aged fibroblasts and in untreated, KU60019-treated, and KU60019 + 17DMAG–treated young fibroblasts. n = 3 independent experiments.

(D) Immunoblot analysis of BAX and BCL-2 protein levels in aged fibroblasts and in untreated, KU60019-treated, and KU60019 + 17DMAG–treated young fibroblasts. n = 3 independent experiments.

Data are shown as mean ± SEM. *P* values were calculated using Welch's ANOVA followed by Holm-adjusted Games–Howell post hoc comparisons. N.S., not significant.

Table S1. List of compounds and corresponding synapsin-GFP fluorescence intensity values from the compound screening, related to Figure 1. (Excel file)

Table S2. List of differentially expressed genes in hiPSC-derived dopaminergic neurons following KU60019 treatment, related to Figure 4 and Figure S8. (Excel file)

Table S3. RNA-seq–based comparison of gene expression in untreated young fibroblasts, KU60019-treated young fibroblasts, and aged fibroblasts, related to Figure 4 and Figure S8. (Excel file)
