# Supplementary material for: Suppression of ATM kinase signaling accelerates cellular senescence
Source: Stem Cell Reports. 2026 Jun 11;21(7):102956. doi: 10.1016/j.stemcr.2026.102956 (PMC13385434; doi:10.1016/j.stemcr.2026.102956)
Supplement: Document S2. Article plus supplemental information [file mmc5.pdf]

STEM CELL REPORTS
Article
OPEN ACCESS

Suppression of ATM kinase signaling accelerates cellular senescence

Kei-ichi Ishikawa,[1,2,*] Takahiro Shiga,[1] Takumi Hirose,[1] Naoko Kuzumaki,[3,7] Sakura Miyoshi,[3] Akihiro Yamaguchi,[1,2] Hidetaka Tamune,[4,8] Avijite Kumer Sarkar,[5] Kento Nakai,[5] Kazuyoshi Baba,[5] Shigeo Okabe,[4] Nobutaka Hattori,[1,2,6] Hideyuki Okano,[3] and Wado Akamatsu[1,9,*]
[1]Center for Genomic and Regenerative Medicine, Juntendo University School of Medicine, Tokyo, Japan
[2]Department of Neurology, Juntendo University School of Medicine, Tokyo, Japan
[3]Department of Physiology, Keio University School of Medicine, Tokyo, Japan
[4]Department of Cellular Neurobiology, Graduate School of Medicine and Faculty of Medicine, The University of Tokyo, Tokyo, Japan
[5]Department of Prosthodontics, School of Dentistry, Showa University, Tokyo, Japan
[6]Neurodegenerative Disorders Collaborative Laboratory, RIKEN Center for Brain Science, Wako, Saitama, Japan
[7]Present address: Department of Pharmacology, Hoshi University School of Pharmacy and Pharmaceutical Sciences, Tokyo, Japan
[8]Present address: Department of Psychiatry and Behavioral Science, Juntendo University Graduate School of Medicine, Tokyo, Japan
[9]Lead contact
*Correspondence: kishikaw@juntendo.ac.jp (K.-i.I.), awado@juntendo.ac.jp (W.A.)
https://doi.org/10.1016/j.stemcr.2026.102956

SUMMARY

Cells derived from rejuvenated human induced pluripotent stem cells (hiPSCs) require extended culture periods to achieve functional maturation, and it remains difficult to recapitulate cellular senescence in these cells *in vitro*. This limitation hinders the accurate and efficient modeling of age-related neurodegenerative diseases. Here, we aimed to establish a simple approach to promote neuronal maturation and improve the efficiency of hiPSC-based disease modeling. Using a small-molecule inhibitor library, we identified an ATM kinase inhibitor, KU60019, that promotes both maturation-associated features and senescence-associated phenotypes in hiPSC-derived neurons and fibroblasts. KU60019 treatment promoted the manifestation of disease-relevant phenotypes in hiPSC models of age-related neurodegenerative diseases. Furthermore, senolytic analyses suggested that KU60019-induced senescent cells depend on pro-survival pathways, including HSP90-associated signaling. These findings suggest that KU60019 provides a simple and useful tool for accelerating phenotypic recapitulation in hiPSC models of age-related neurodegenerative diseases.

INTRODUCTION

Human induced pluripotent stem cell (hiPSC)-based disease models are particularly valuable in neurological disease research because they recapitulate human nervous system pathology *in vitro*. iPSCs, reprogrammed from somatic cells into pluripotent stem cells, are similar to embryonic stem cells. However, most neurodegenerative diseases, such as Alzheimer's disease (AD) and Parkinson's disease (PD), typically develop after the age of 50. Although pathological changes begin at the cellular level in the prodromal stage, long-term culture is required to reproduce pathological phenotypes in hiPSC models (Okano and Morimoto, 2022). Moreover, although maturation of hiPSC-derived neurons progresses during the culture period, it takes more than 7 weeks to achieve spontaneous activity characteristic of mature neurons in adherent cultures, and this activity declines after 30 weeks (Odawara et al., 2016). Cortical organoids cultured for over 40 weeks recapitulate aspects of *in vivo* neuron maturation but resemble premature infant brains (24–38 weeks postmenstrual age) (Trujillo et al., 2019). Thus, reproducing aging in hiPSC-derived neurons through long-term culture remains challenging, and novel strategies to induce senescence are required to more accurately model age-related neurodegenerative diseases (de Luzy et al., 2024; Jothi and Kulka, 2024).

Several strategies have been proposed to induce artificial senescence by regulating aging-related genes, including the introduction of progerin (Miller et al., 2013), knockdown of SATB1 (a nuclear matrix protein) (Riessland et al., 2019; Russo et al., 2024), and knockdown of RANBP17 (a nuclear transport receptor) (Mertens et al., 2015). Although these approaches can induce senescence-like phenotypes in hiPSC-derived neurons, they are technically complex and difficult to standardize in terms of throughput, efficiency, and uniformity. Dopaminergic neurons derived from telomerase inhibitor-treated iPSCs exhibited aging-associated phenotypes. However, although long-term culture reduced the number of tyrosine hydroxylase (TH)-positive neurons in PD-derived cells, a similar effect was observed in control neurons (Vera et al., 2016). Drug screening using neonatal fibroblasts identified an inhibitor cocktail comprising a ULK1 inhibitor (SBI-0206965), which suppresses autophagy, and a DNA glycosylase inhibitor (O151), which impairs DNA repair. This combination has been reported to promote senescence-like and disease-associated phenotypes in hiPSC-derived neurons from patients with amyotrophic lateral sclerosis (Fathi et al., 2022). Recently, a genome-wide CRISPR screen using iPSC-derived neurons carrying familial AD mutation, with cell death as a readout, identified neddylation-related genes. Treatment with a neddylation inhibitor, MLN4924, has been shown

Published by Elsevier Inc. on behalf of International Society for Stem Cell Research.
This is an open access article under the CC BY license (http://creativecommons.org/licenses/by/4.0/).

to promote aging-associated and disease-relevant phenotypes of neurodegenerative diseases in iPSC-derived neurons (Saurat et al., 2024).

In this study, we aimed to identify compounds that promote the maturation of hiPSC-derived neurons and improve the efficiency of modeling late-onset neurodegenerative diseases. We identified an ATM (ataxia telangiectasia mutated) kinase inhibitor, KU60019, that promotes neuronal maturation. ATM is a central regulator of the DNA damage response, and its inhibition accelerates the manifestation of disease-relevant phenotypes by inducing an aging-like state in hiPSC-derived neurons.

RESULTS

Screening of compounds that promote the maturation of hiPSC-derived neurons

To identify compounds that accelerate the maturation of hiPSC-derived neurons, we screened an inhibitor library containing 111 compounds (Sigma-Aldrich, BMINH02MARUN). An overview of the screening process is presented in Figure 1A. Neurons were generated from healthy human iPSCs (201B7) using neurosphere-based differentiation. Neural progenitors derived from neurospheres were transduced with a lentiviral vector encoding a GFP reporter driven by the synapsin promoter (*Synapsin*-GFP) on day 0. The synapsin promoter was used as a readout of neuronal maturation, as synapsin expression increases during synapse formation and functional maturation. Compound treatment was initiated at this stage, when cells remained immature neuronal progenitors, to capture the effects of compounds on the transition toward more mature neuronal phenotypes. After 24 h, the medium was replaced with fresh medium containing 10 μM of each compound, and neuronal maturation was evaluated on day 17 based on the fluorescence intensity of *Synapsin*-GFP (Figure 1A; Table S1). The highest fluorescence intensity relative to astrocyte-conditioned medium (ACM), used as a positive control, was observed in neurons treated with KU60019, an ATM kinase inhibitor (Figure 1B).

To investigate the effects and safety of KU60019 on hiPSC-derived neurons, hiPSC-derived midbrain dopaminergic neurons were treated with this inhibitor. Because treatment with 10 μM KU60019 was neurotoxic, the treatment concentration was set to 5 μM (Figure S1). KU60019 treatment (5 μM) did not significantly affect the percentages of cells positive for the neuronal marker β3-tubulin, the dopaminergic neuron marker TH, and the midbrain dopaminergic neuron progenitor cell marker FOXA2 (Figures 1C–1F). These results indicate that KU60019 does not alter neuronal identity or lineage specification during differentiation of hiPSCs into dopaminergic neurons. Furthermore, we examined the effect of KU60019 on neuronal maturation by measuring neurite length. Dopaminergic neurons treated with KU60019 exhibited significantly longer neurites than untreated neurons after 5 and 7 days of treatment (Figures 1G and 1H). Functional maturation was subsequently assessed using patch-clamp recordings. Dopaminergic neurons treated with KU60019 exhibited higher frequencies of action potential firing in response to current injection than untreated neurons at day 35 (Figure 1I). Collectively, these results suggest that KU60019 promotes the maturation of hiPSC-derived dopaminergic neurons without affecting their differentiation efficiency.

KU60019 induces senescence-associated phenotypes in hiPSC-derived dopaminergic neurons

KU60019 is an ATM kinase inhibitor, and the ATM pathway has been implicated in cellular senescence (Dong et al., 2022). Given that KU60019 was identified as a maturation-promoting compound in our screen, we hypothesized that it may influence senescence-related processes in hiPSC-derived dopaminergic neurons. To test this possibility, we performed staining for senescence-associated β-galactosidase (SA-βGal), a marker of senescent cells. KU60019 treatment significantly increased the number of SA-βGal-positive dopaminergic neurons (Figures 2A and 2B). Because SA-βGal staining can also be induced by cellular stress, additional markers were analyzed to further characterize SA-βGal-positive cells (Alessio et al., 2021; Yang and Hu, 2005). Most SA-βGal-positive cells were classified as senescent (pRPS6$^+$, Ki67$^-$) or pre-senescent (pRPS6$^-$) cells. KU60019 treatment predominantly increased the proportion of senescent cells, whereas stressed (pRPS6$^+$, Ki67$^+$) cells were rarely detected (Figures S2A and S2B). This trend was consistently observed across the two hiPSC-derived neuronal lines examined.

To further assess KU60019-induced senescence in hiPSC-derived dopaminergic neurons, we evaluated multiple senescence-associated phenotypes, including increased DNA damage, abnormal nuclear morphology, altered lamin B1 and B2 expression, proteolytic dysfunction, altered NAD metabolism, and the formation of cytoplasmic chromatin fragments (CCFs). In the immunostaining-based analyses, quantification was performed in MAP2-positive cells. KU60019 treatment significantly increased the number of neurons with three or more γH2AX foci in the nucleus, indicating DNA damage accumulation (Figures 2C and 2D). KU60019 treatment for more than 7 days also increased the number of cells with abnormal nuclear membrane structures, as indicated by reduced lamin B1 fluorescence (Figures 2E and 2F). Moreover, lamin B1 and B2 expression levels showed a decreasing trend (Figures 2G and 2H). Furthermore, KU60019 treatment

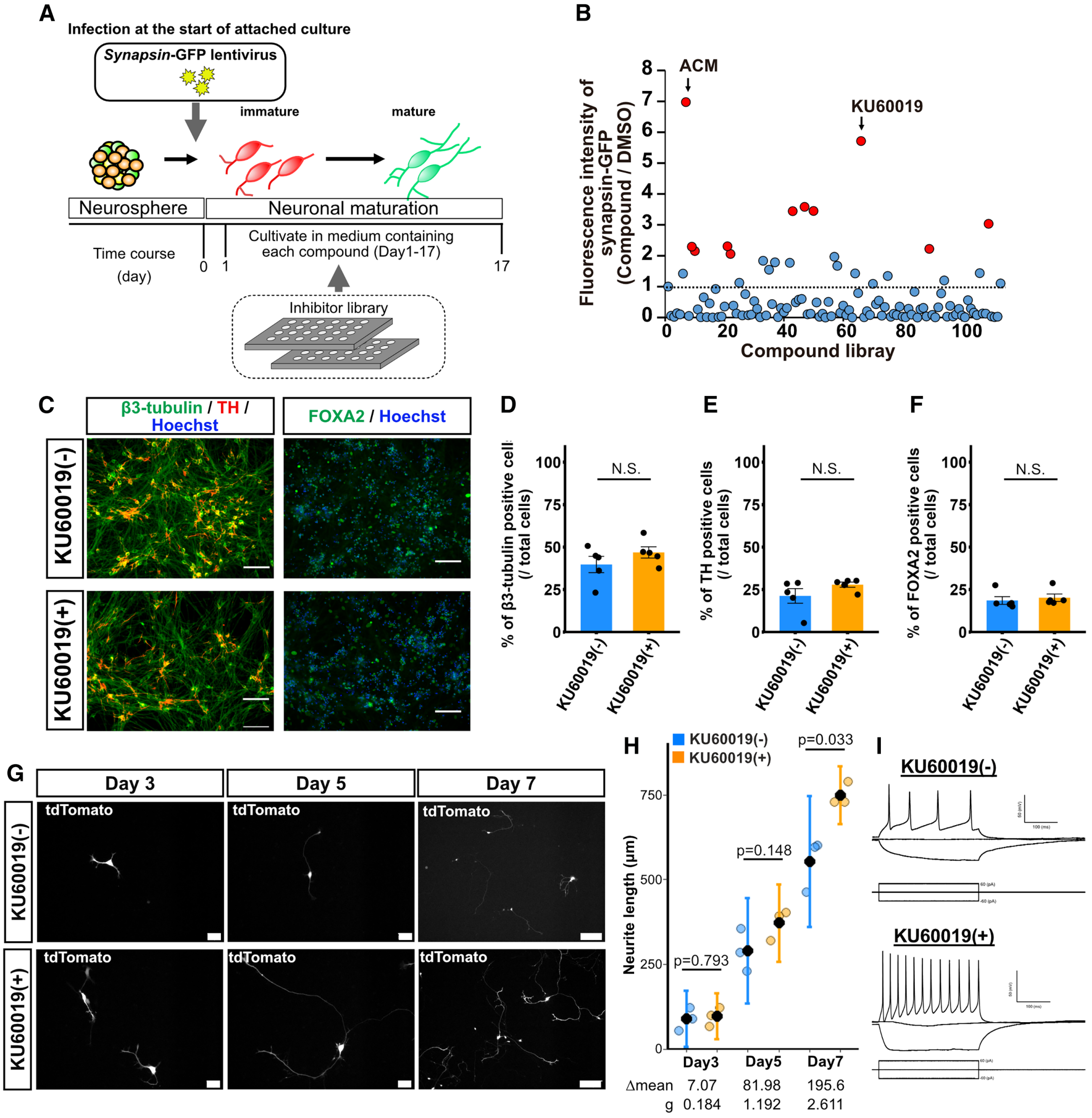

Figure 1. Screening identifies compounds that promote the maturation of hiPSC-derived neurons

(A) Schematic overview of the compound screening strategy using Synapsin-GFP as a reporter of neuronal maturation in hiPSC-derived neurons.

(B) Screening results based on Synapsin-GFP fluorescence intensity.

(C) Representative images of immunostaining for the neuronal marker β3-tubulin, the dopaminergic marker TH, and the midbrain progenitor marker FOXA2 in untreated and KU60019-treated dopaminergic neurons after 14 days of differentiation.

(D–F) Quantification of β3-tubulin-, TH-, and FOXA2-positive cells. Scale bars, 100 μm. $n = 5$ independent experiments.

(G) Representative images of untreated and KU60019-treated dopaminergic neurons labeled with AAV1-tdTomato for neurite length analysis. Scale bars: 50 μm (days 3 and 5) and 200 μm (day 7).

(H) Dot plot showing neurite length. Data were obtained from $n = 3$ independent experiments (11–46 cells analyzed per experiment). Black dots indicate means, and error bars represent 95% confidence intervals. Mean differences (Δmean) and effect sizes (Hedges' g) are indicated.

(I) Representative membrane potential traces during hyperpolarizing and depolarizing current injections (−60, 0, and 60 pA for 300 ms) recorded on day 35. Repetitive action potentials were evoked by injection of a 60-pA current. Unless otherwise indicated, data are shown as mean ± SEM. p values were calculated using Welch's t test. N.S., not significant. See also Figure S1 and Table S1.

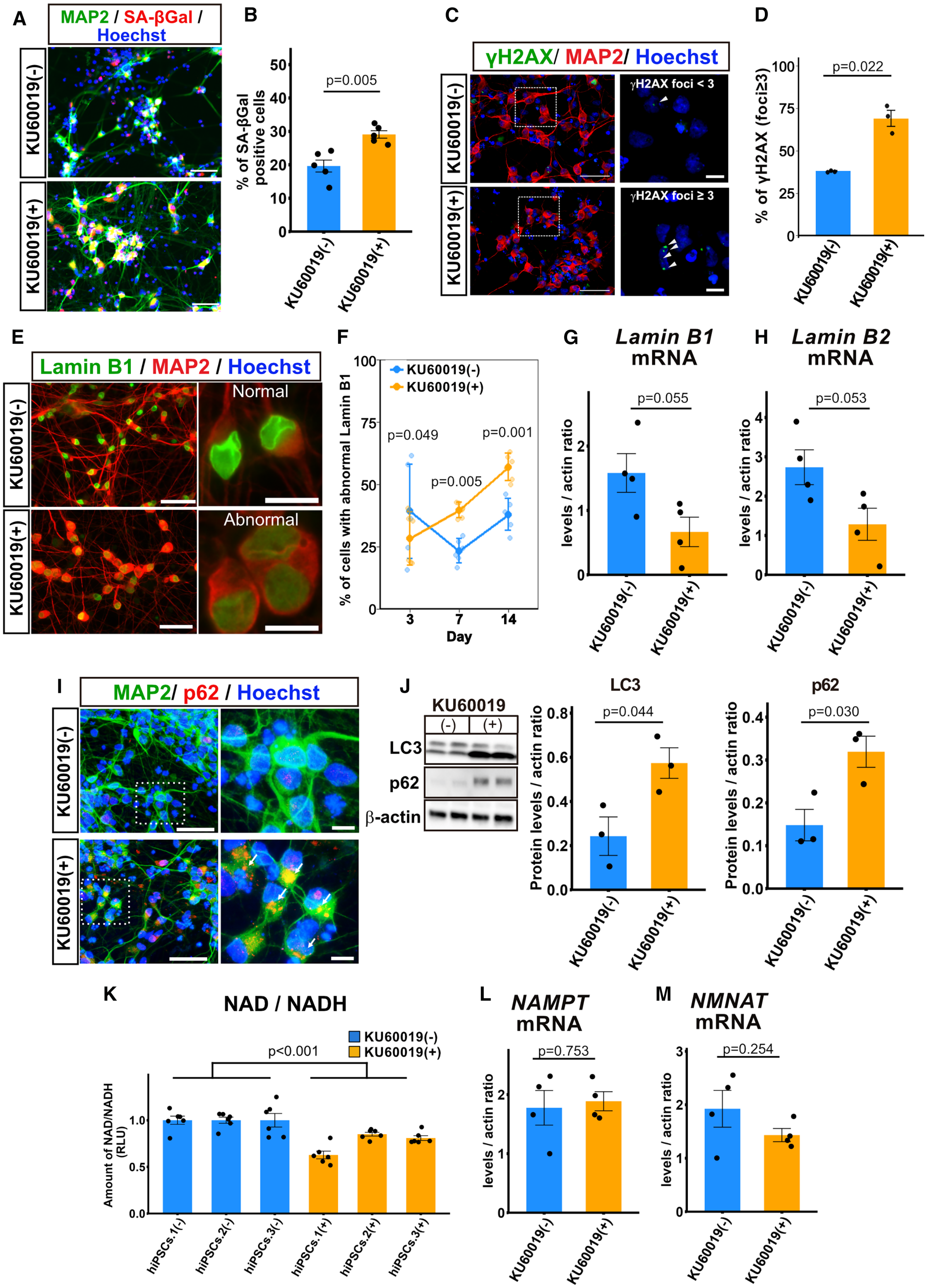

Figure 2. KU60019 induces senescence-associated phenotypes in hiPSC-derived dopaminergic neurons

(A) Representative images of SA-βGal staining and MAP2 immunostaining in untreated and KU60019-treated hiPSC-derived dopaminergic neurons after 14 days of differentiation. Scale bars, 100 μm.

(legend continued on next page)

increased the intracellular accumulation of the autophagy substrate p62 and upregulated the expression of the autophagosome marker LC3-II in dopaminergic neurons, suggesting autophagic dysfunction (Figures 2I and 2J). Compared with untreated cells, KU60019 treatment decreased the NAD/NADH ratio in dopaminergic neurons; however, the expression levels of NAMPT and NMNAT, key enzymes in NAD biosynthesis, were unaffected (Figures 2K–2M). Although KU60019 treatment did not significantly increase CCF formation when assessed using multiple markers (Ivanov et al., 2013), most effect sizes were moderate to large, suggesting a potential increase across the two hiPSC-derived neuronal lines (Figures S2C–S2E). Overall, these results are consistent with previous findings in physiologically senescent cells (López-Otín et al., 2023) and suggest that KU60019 induces senescence-like phenotypes in hiPSC-derived neurons, a property that was not the primary focus of the maturation-based screen.

To confirm that the effects of KU60019 were independent of the applied differentiation method, the floor plate method was used to obtain dopaminergic neurons (Gantner et al., 2020). Consistent with the results obtained using neurosphere-based differentiation, KU60019 treatment induced cellular senescence-associated phenotypes in dopaminergic neurons generated by the floor plate method without affecting differentiation (Figure S3).

KU60019 induces aging-associated phenotypes in young fibroblasts resembling physiological aging

To evaluate the similarity between KU60019-induced and physiologically aged senescence, we treated young fibroblasts from a healthy 17-year-old donor with KU60019 and compared their phenotypes with those of aged fibroblasts from a healthy 66-year-old donor. The percentage of SA-βGal-positive cells was low in young fibroblasts (13 ± 2.77%), but high in aged fibroblasts (40 ± 2.16%). However, treatment of young fibroblasts with KU60019 increased the percentage of SA-βGal-positive cells to 81 ± 3.54% (Figures 3A and 3B). Further analysis confirmed that this increase was mainly due to an expansion of pre-senescent and senescent cell populations, whereas stressed cells increased only minimally (Figures S4A and S4B). γH2AX and 53BP1 foci are markers of double-strand DNA breaks (DSBs), which decrease with aging in quiescent cells, including mature neurons, but increase with aging in proliferating cells, such as fibroblasts (Zorin et al., 2019). KU60019 treatment reduced γH2AX and 53BP1 foci in young fibroblasts, suggesting impaired DNA damage responses (Figures 3C–3E). Consistent with results in aged fibroblasts, KU60019 treatment reduced lamin B1 localization at the nuclear rim in young fibroblasts (Figures 3F and 3G). Changes in autophagy were assessed based on p62 and LC3 levels. Aged fibroblasts exhibited higher levels of p62 and LC3-II than young fibroblasts; similarly, KU60019 treatment increased p62 and LC3-II levels in young fibroblasts (Figures 3H and 3I). Analysis of CCF formation using multiple markers showed that KU60019 treatment significantly increased CCF formation for most markers examined (Figures S4C–S4F). To further examine SASP following KU60019 treatment, we performed a cytokine array analysis of conditioned media. A coordinated trend toward increased secretion of multiple cytokines, including IL-1α, IL-1β, IL-8, TGF-β, and TNF-α, was observed (Figure S5). Additionally, KU60019 treatment induced similar changes in senescence-associated markers, such as lamin B1 and p62, in SH-SY5Y cells (Figure S6). These results indicate that KU60019 induces

(B) Percentage of SA-βGal^{+} cells. $n = 5$ independent experiments.
(C) Representative images of γH2AX and MAP2 immunostaining in untreated and KU60019-treated neurons on day 14. Magnified views are shown on the right. Scale bars: 100 μm (left) and 10 μm (right).
(D) Percentage of γH2AX-positive cells. Nuclei containing ≥3 γH2AX foci were defined positive. Data were obtained from $n = 3$ independent experiments (77–192 cells analyzed per experiment).
(E) Representative images of lamin B1 and MAP2 immunostaining in untreated and KU60019-treated neurons on day 14. Magnified views are shown on the right. Scale bars: 100 μm (left) and 10 μm (right).
(F) Percentage of cells with abnormal nuclear morphology at days 3, 7, and 14. Data are shown as mean ±95% CI from $n = 6$ independent experiments. Statistical significance was assessed using two-way ANOVA (treatment × time), followed by Holm-adjusted post hoc comparisons at each time point.
(G and H) Gene expression levels of lamin B1 (G) and lamin B2 (H) in untreated and KU60019-treated neurons measured on day 23. $n = 4$ independent experiments.
(I) Representative images of MAP2 and p62 immunostaining in untreated and KU60019-treated neurons on day 14. Magnified views are shown on the right. Scale bars: 100 μm (left) and 20 μm (right).
(J) Immunoblot analysis of LC3 and p62 expression in untreated and KU60019-treated neurons on day 14. $n = 3$ independent experiments.
(K) NAD/NADH ratio in untreated and KU60019-treated neurons on day 14. $n = 6$ independent experiments.
(L and M) Gene expression levels of the NAD biosynthesis enzymes NAMPT (L) and NMNAT (M) in untreated and KU60019-treated neurons on day 14. $n = 4$ independent experiments. Unless otherwise indicated, data are shown as mean ± SEM. p values were calculated using Welch's t test, except for (F). See also Figures S2 and S3.

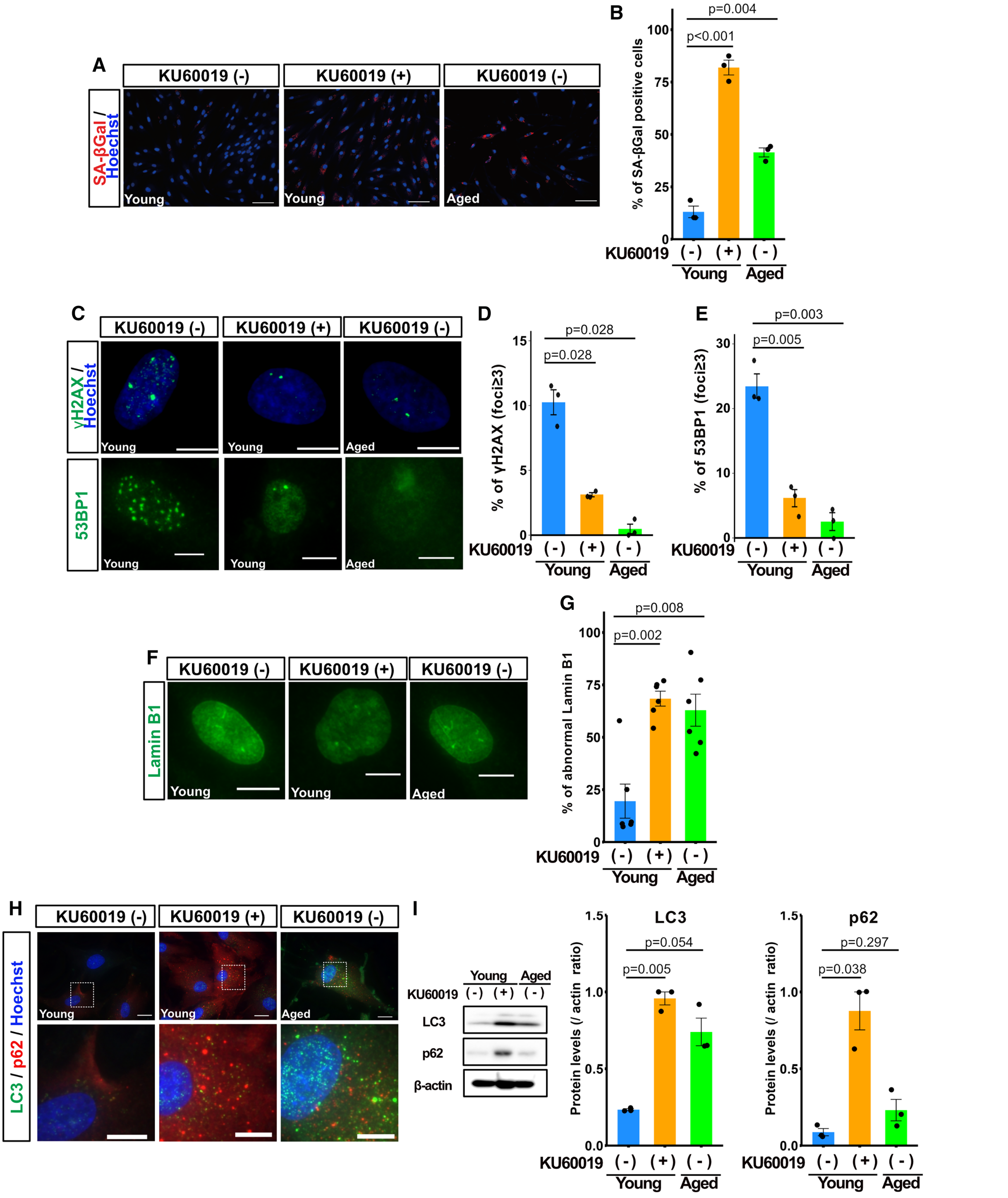

Figure 3. KU60019 induces aging-associated phenotypes in young fibroblasts resembling physiological aging

(A) Representative images of SA-βGal staining in untreated young, KU60019-treated young, and aged fibroblasts. Scale bars, 100 μm.

(B) Percentage of SA-βGal^{+} cells. n = 3 independent experiments.

(C) Representative images of γH2AX (upper images) and 53BP1 (lower images) immunostaining. Scale bars, 10 μm.

(D and E) Percentage of nuclei containing ≥3 γH2AX foci (D) or ≥3 53BP1 foci (E). n = 3 independent experiments.

(F) Representative images of lamin B1 immunostaining. Scale bars, 10 μm.

(legend continued on next page)

cellular senescence-associated phenotypes across multiple cell types.

ATM, ATR (ataxia-telangiectasia and Rad3-related), and the MRN complex (MRE11, RAD50, and NBS1) are central components of the DNA damage response (DDR) to DSBs and coordinate DNA repair and cell-cycle progression (Figure S7A). To determine whether inhibition of other DDR-related components similarly promotes senescence-associated phenotypes, we examined the effects of additional ATM inhibitors (KU55933 and AZD0156), an ATR inhibitor (VE821), and an MRN complex inhibitor (MIRIN). KU60019, AZD0156, and MIRIN significantly increased the proportion of SA-βGal-positive cells in young fibroblasts, with KU60019 showing the strongest effect (Figures S7B and S7C).

KU60019 alters senescence-associated gene expression

To evaluate global gene expression changes associated with KU60019-induced senescence, we performed RNA-sequencing of hiPSC-derived dopaminergic neurons treated with or without KU60019 (Table S2). Principal component analysis (PCA) showed that KU60019 treatment significantly altered the gene expression profiles of these neurons compared with untreated controls. The third principal component (PC3) primarily reflected the effect of KU60019 treatment (Figure 4A). Notably, KU60019 treatment resulted in the upregulation of 92 genes and downregulation of 489 genes in dopaminergic neurons (Figure 4B). Kyoto Encyclopedia of Genes and Genomes (KEGG) pathway analysis revealed that genes downregulated by KU60019 treatment were enriched in pathways related to the cell cycle, p53 signaling, cellular senescence, DNA replication, homologous recombination (HR), and the Fanconi anemia pathway (Figure 4C). These transcriptional changes are consistent with stable cell-cycle arrest and suppression of proliferation-associated DNA replication and repair programs, which are characteristic features of senescent cells. Although the “cellular senescence” pathway was also identified among the downregulated pathways, this likely reflects suppression of proliferation- and DNA repair-related components included in the pathway rather than an absence of senescence. Together with the observed senescence-associated phenotypes, these results indicate that KU60019 induces a senescence-like transcriptional program in hiPSC-derived dopaminergic neurons.

Furthermore, we performed RNA-sequencing to elucidate the mechanism by which KU60019 promotes senescence-associated changes in fibroblasts (Table S3). KU60019 treatment upregulated 629 genes and downregulated 630 genes in young fibroblasts (Figure 4D). Compared with young fibroblasts, 713 and 715 genes were upregulated and downregulated, respectively, in aged fibroblasts (Figure 4E). PCA showed that KU60019 significantly altered the gene expression profile of young fibroblasts compared with untreated young fibroblasts (Figure 4F). PC1 primarily reflected donor differences between young and aged cells. In contrast, PC2 and PC3 showed that the overall gene expression of KU60019-treated young fibroblasts shifted toward that of aged fibroblasts, separated from untreated young cells. Clustering analyses by dendrograms and heatmaps revealed that young fibroblasts treated with KU60019 had a gene expression pattern similar to physiologically aged fibroblasts (Figures 4G and 4H). These results indicate that KU60019 induces senescence-associated transcriptional changes in young fibroblasts, similar to that observed in physiologically aged cells. Consistent with results in hiPSC-derived neurons, KEGG analysis showed that genes downregulated in KU60019-treated young fibroblasts were enriched in pathways related to the cell cycle, DNA replication, the Fanconi anemia pathway, protein digestion and absorption, HR, base excision repair, and mismatch repair (Figure 4I). These transcriptional changes are consistent with stable cell-cycle arrest and suppression of proliferation-associated DNA replication and DNA repair programs, which are characteristic features of senescent cells. Additionally, upregulated genes were enriched in the lysosomal pathway (Figure 4J), consistent with previous findings that lysosomal protein expression, such as v-ATPase, is increased following ATM inhibition (Kang et al., 2017). Across hiPSC-derived dopaminergic neurons and young fibroblasts treated with KU60019, 190 genes were commonly altered (Figures S8A and S8B). Most of the genes were downregulated by KU60019 treatment (Figure S8A), and the top enriched KEGG pathways were related to the cell cycle, the Fanconi anemia pathway, cellular senescence, p53 signaling, HR, and DNA replication (Figures S8C and S8D). These shared transcriptional changes likely reflect coordinated suppression of proliferation- and replication-associated components within these pathways rather than the absence of senescence. Together, these results indicate that KU60019 induces conserved senescence-associated transcriptional programs across multiple cell types.

Analysis of survival pathways in KU60019-induced senescence reveals HSP90 dependence

To further investigate the mechanisms underlying KU60019-induced senescence, we examined the effects of

(G) Percentage of cells with abnormal nuclear morphology. $n = 6$ independent experiments.

(H) Representative images of LC3 and p62 immunostaining.

(I) Immunoblot analysis of LC3 and p62 expression. $n = 3$ independent experiments. Data are shown as mean ± SEM. p values were calculated using Welch’s ANOVA followed by Holm-adjusted Games-Howell post hoc comparisons. See also Figures S4–S7.

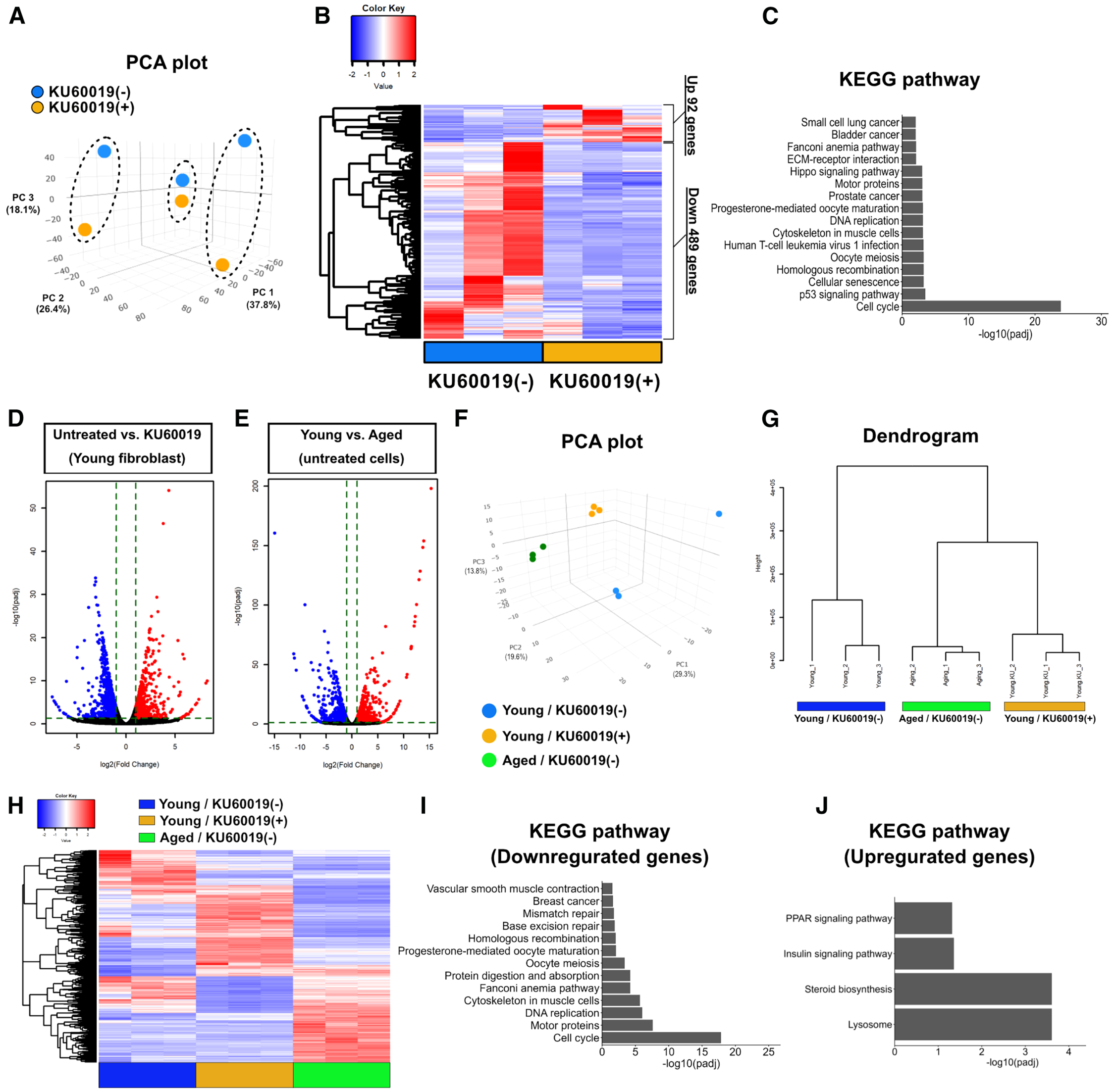

Figure 4. KU60019 induces senescence-associated and DNA damage response-related transcriptional changes

(A) Three-dimensional PCA plots of normalized transcriptome data from three hiPSC-derived dopaminergic neuronal lines (blue) and KU60019-treated neurons (orange). Dashed circles indicate samples derived from the same hiPSC line.

(B) Heatmap of significantly altered genes (adjusted $p < 0.05$) in neurons following KU60019 treatment.

(C) KEGG pathway enrichment analysis of genes significantly downregulated following KU60019 treatment in neurons. Bars represent fold enrichment.

(D and E) Volcano plots of untreated versus KU60019-treated young fibroblasts (D) and young versus aged fibroblasts (E). Differentially expressed genes (adjusted $p < 0.05$) are highlighted in blue (downregulated) and red (upregulated).

(F) Three-dimensional PCA plots of normalized transcriptome data from untreated young (blue), KU60019-treated young (orange), and aged (green) fibroblasts.

(G) Hierarchical clustering dendrograms of transcriptome data from young, KU60019-treated young, and aged fibroblasts.

(H) Heatmap of differentially expressed genes in young, KU60019-treated young, and aged fibroblasts.

(I and J) KEGG pathway enrichment analysis of genes significantly downregulated (I) and upregulated (J) in young fibroblasts following KU60019 treatment. Bars represent fold enrichment. Adjusted p values were calculated using likelihood ratio tests (LRTs) implemented in edgeR. See also Figure S8 and Tables S2 and S3.

compounds reported to exhibit senolytic activity, which selectively induce the death of senescent cells, in combination with KU60019. We assessed the viability of KU60019-treated young fibroblasts co-treated with seven such compounds, including rapamycin (Laberge et al., 2015), metformin (Moiseeva et al., 2013), ABT-737 (Yosef et al.,

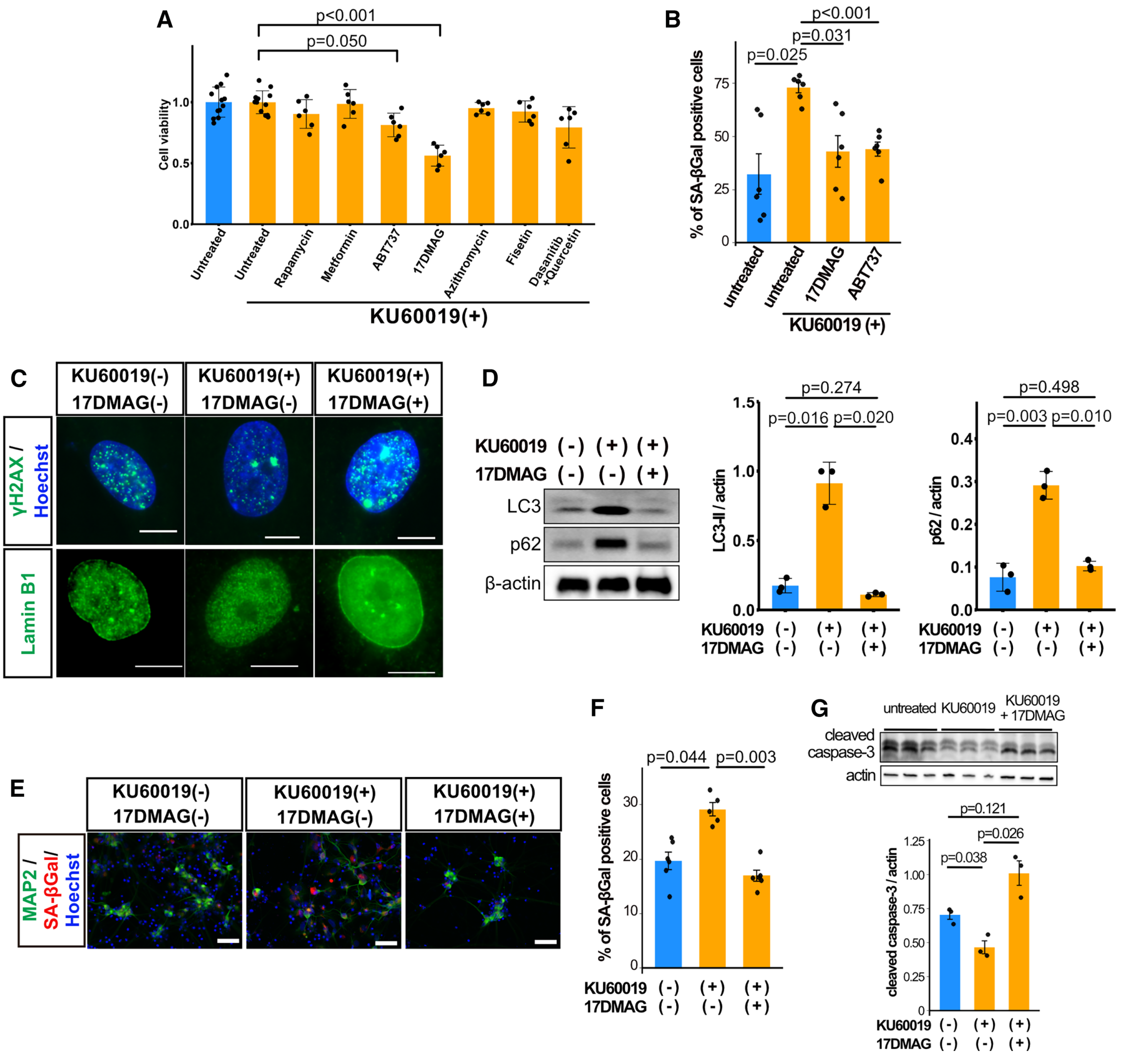

Figure 5. HSP90 and BCL-2 inhibition induce cell death in KU60019-treated cells and suppress senescence-associated phenotypes
(A) Cell viability of young fibroblasts treated with the indicated compounds for 24 h in the absence or presence of KU60019. Blue bars represent untreated young fibroblasts, and orange bars represent KU60019-treated young fibroblasts co-treated with the indicated compounds. $n = 6$ independent experiments.
(B) Percentage of SA-βGal^{+} cells under the indicated conditions (untreated, KU60019, KU60019 + 17DMAG, and KU60019 + ABT-737) in young fibroblasts. $n = 6$ independent experiments.
(C) Representative images of γH2AX (upper images) and lamin B1 (lower images) immunostaining in young fibroblasts under the indicated conditions. Scale bars, 10 μm.
(D) Immunoblot analysis of LC3 and p62 protein levels in young fibroblasts under the indicated conditions. $n = 3$ independent experiments.
(E) Representative images of SA-βGal and MAP2 staining in untreated, KU60019-treated, and KU60019 + 17DMAG-treated hiPSC-derived neurons after 14 days of differentiation. Scale bars, 100 μm.
(F) Percentage of SA-βGal^{+} neurons shown in (E). $n = 5–6$ independent experiments.
(G) Immunoblot analysis of cleaved caspase-3 protein levels in untreated, KU60019-treated, and KU60019 + 17DMAG-treated hiPSC-derived neurons. $n = 3$ independent experiments. Data are shown as mean ± SEM. p values were calculated using Welch's ANOVA followed by Holm-adjusted Games-Howell post hoc comparisons. See also Figure S9.

2016), 17DMAG (Fuhrmann-Stroissnigg et al., 2017), azithromycin (Ozsvari et al., 2018), fisetin (Yousefzadeh et al., 2018), and a dasatinib-quercetin combination (Zhu et al., 2015). Among these, ABT-737, a BCL-2 family inhibitor, and 17DMAG, an HSP90 inhibitor, reduced the viability of KU60019-treated fibroblasts, with 17DMAG showing the strongest effect (Figure 5A). The senolytic activity of both compounds was further supported by their

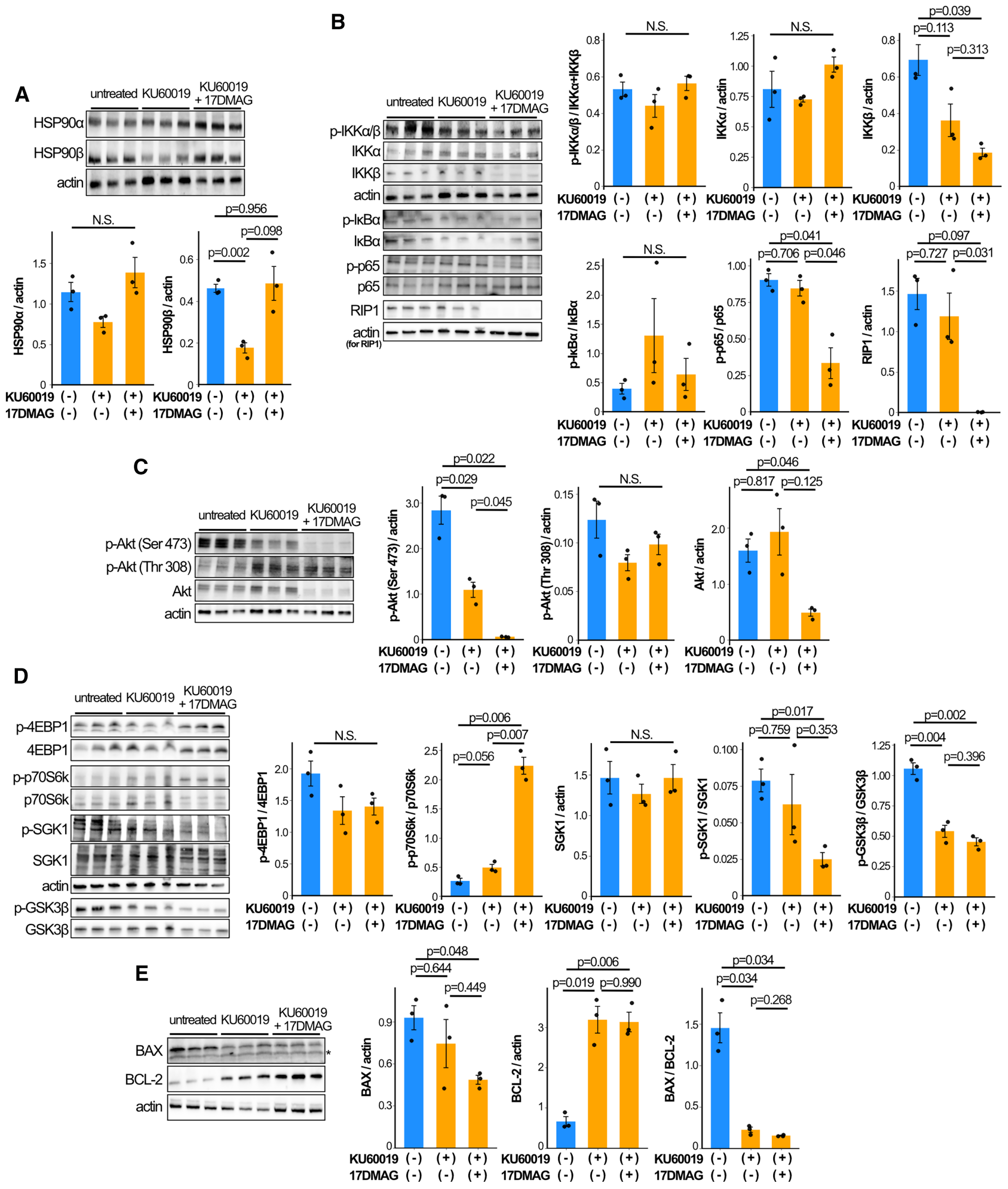

Figure 6. KU60019-induced senescence-associated changes are linked to alterations in NF-κB, Akt, and BCL-2 signaling in neurons

(A) Immunoblot analysis of HSP90 protein levels in untreated, KU60019-treated, and KU60019 + 17DMAG-treated hiPSC-derived neurons. n = 3 independent experiments.

(B) Immunoblot analysis of NF-κB signaling components and their phosphorylated forms in untreated, KU60019-treated, and KU60019 + 17DMAG-treated neurons. n = 3 independent experiments.

(C) Immunoblot analysis of Akt and phosphorylated Akt protein levels in untreated, KU60019-treated, and KU60019 + 17DMAG-treated neurons. n = 3 independent experiments.

(legend continued on next page)

ability to reduce the number of KU60019-induced SA-βGal-positive fibroblasts (Figure 5B). These results indicate that both compounds exert senolytic effects on KU60019-induced senescent cells.

We next focused on the effects of 17DMAG on KU60019-induced senescent cells. 17DMAG reversed KU60019-induced senescence-associated phenotypes in fibroblasts, including increased γH2AX foci, abnormal lamin B1 distribution, and autophagic dysfunction (Figures 5C and 5D). In hiPSC-derived neurons, 17DMAG abolished the KU60019-induced increase in SA-βGal-positive cells (Figures 5E and 5F). In addition, 17DMAG restored caspase-3/7 activity in KU60019-treated fibroblasts (Figure S9B) and increased cleaved caspase-3 levels in KU60019-treated neurons derived from two independent hiPSC lines (Figures 5G and S9A), indicating the induction of apoptosis.

To further investigate HSP90-related pathways, we examined HSP90 isoform expression in neurons derived from two independent hiPSC lines and in fibroblasts. KU60019 treatment did not alter HSP90α expression, whereas HSP90β expression was either unchanged or modestly decreased, depending on the cell type (Figures 6A, S10A, and S11A). Upon co-treatment with 17DMAG, the expression of both HSP90 isoforms tended to increase, consistent with a compensatory response to reduced HSP90 activity (Kim et al., 2017). This trend was observed in neurons derived from two independent hiPSC lines (Figures 6A and S10A) as well as in fibroblasts (Figure S11A), although statistical significance varied between conditions.

Given that HSP90 stabilizes numerous client proteins and that its inhibition has been reported to suppress pro-survival signaling pathways, including NF-κB and AKT, we next examined the NF-κB pathway. In hiPSC-derived neurons, KU60019 treatment did not alter the levels of key NF-κB signaling components, including phosphorylated p65 (p-p65), total p65, IKKα, IKKβ, and IκBα (Figure 6B). The expression of RIP1, which functions upstream of the IKK complex as a scaffold protein required for NF-κB activation, was also unchanged (Figure 6B). In contrast, co-treatment with 17DMAG markedly reduced RIP1 and IKKβ levels and decreased p65 phosphorylation, while total p65 levels remained unchanged, indicating suppression of NF-κB signaling (Figure 6B). Similar results were observed in an additional hiPSC-derived neuronal line (Figure S10B) and in fibroblasts (Figure S11B).

We next examined the Akt pathway. Total Akt levels and phosphorylation at Thr308 remained unchanged following KU60019 treatment, whereas phosphorylation at Ser473 was reduced (Figure 6C). Because phosphorylation at Ser473 is required for full Akt activation, these results indicate partial attenuation of Akt signaling. Consistent with this, phosphorylation of GSK3β, a downstream substrate negatively regulated by Akt, was also decreased (Figure 6D). To determine whether this reduction in Akt activity affected mTORC1 signaling, we analyzed phosphorylation of the canonical mTORC1 substrates 4EBP1 and p70S6K (Thr389). Phosphorylation of these substrates remained largely unchanged following KU60019 treatment (Figure 6D). In addition, phosphorylation of SGK1 was not altered (Figure 6D). Together, these findings suggest that partial attenuation of Akt signaling did not substantially affect mTORC1 or SGK1 signaling. Upon co-treatment with 17DMAG, total Akt levels were markedly reduced, and phosphorylation at Ser473 was further diminished (Figure 6C). Notably, phosphorylation of p70S6K at Thr389 was increased compared with KU60019 treatment alone, whereas phosphorylation of 4EBP1 remained largely unchanged, and SGK1 phosphorylation was also not significantly altered (Figure 6D). Together, these results suggest that, while NF-κB and Akt signaling pathways are suppressed under combined ATM and HSP90 inhibition, canonical mTORC1 outputs exhibit differential responses. Similar results were observed in an additional hiPSC-derived neuronal line (Figures S10C and S10D). In contrast, in fibroblasts, KU60019 treatment reduced phosphorylation of Akt at Ser473, but no additional effect was observed upon co-treatment with 17DMAG (Figure S11C). These findings suggest that KU60019 selectively impairs Akt signaling without broadly suppressing downstream growth signaling pathways and reveal cell type-specific differences in Akt regulation downstream of HSP90 inhibition.

Finally, we examined the BCL-2 pathway implicated by ABT-737. Senescent cells are known to upregulate anti-apoptotic members of the BCL-2 family, thereby suppressing apoptosis and conferring resistance to cell death (Basu, 2022). Consistent with this, KU60019 treatment increased BCL-2 expression and reduced the BAX/BCL-2 ratio in hiPSC-derived neurons (Figure 6E). Similar changes were observed in an additional hiPSC-derived neuronal line (Figure S10E) and in fibroblasts (Figure S11D), suggesting that enhanced anti-apoptotic signaling may contribute to the persistence of KU60019-induced senescent cells.

Taken together, these findings indicate that KU60019-induced senescence is maintained by both HSP90-dependent

(D) Immunoblot analysis of Akt-mTOR pathway components and their phosphorylated forms in untreated, KU60019-treated, and KU60019 + 17DMAG-treated neurons. $n = 3$ independent experiments.
(E) Immunoblot analysis of BAX and BCL-2 protein levels in untreated, KU60019-treated, and KU60019 + 17DMAG-treated neurons. $n = 3$ independent experiments. Data are shown as mean ± SEM. p values were calculated using Welch's ANOVA followed by Holm-adjusted Games-Howell post hoc comparisons. See also Figures S10 and S11.

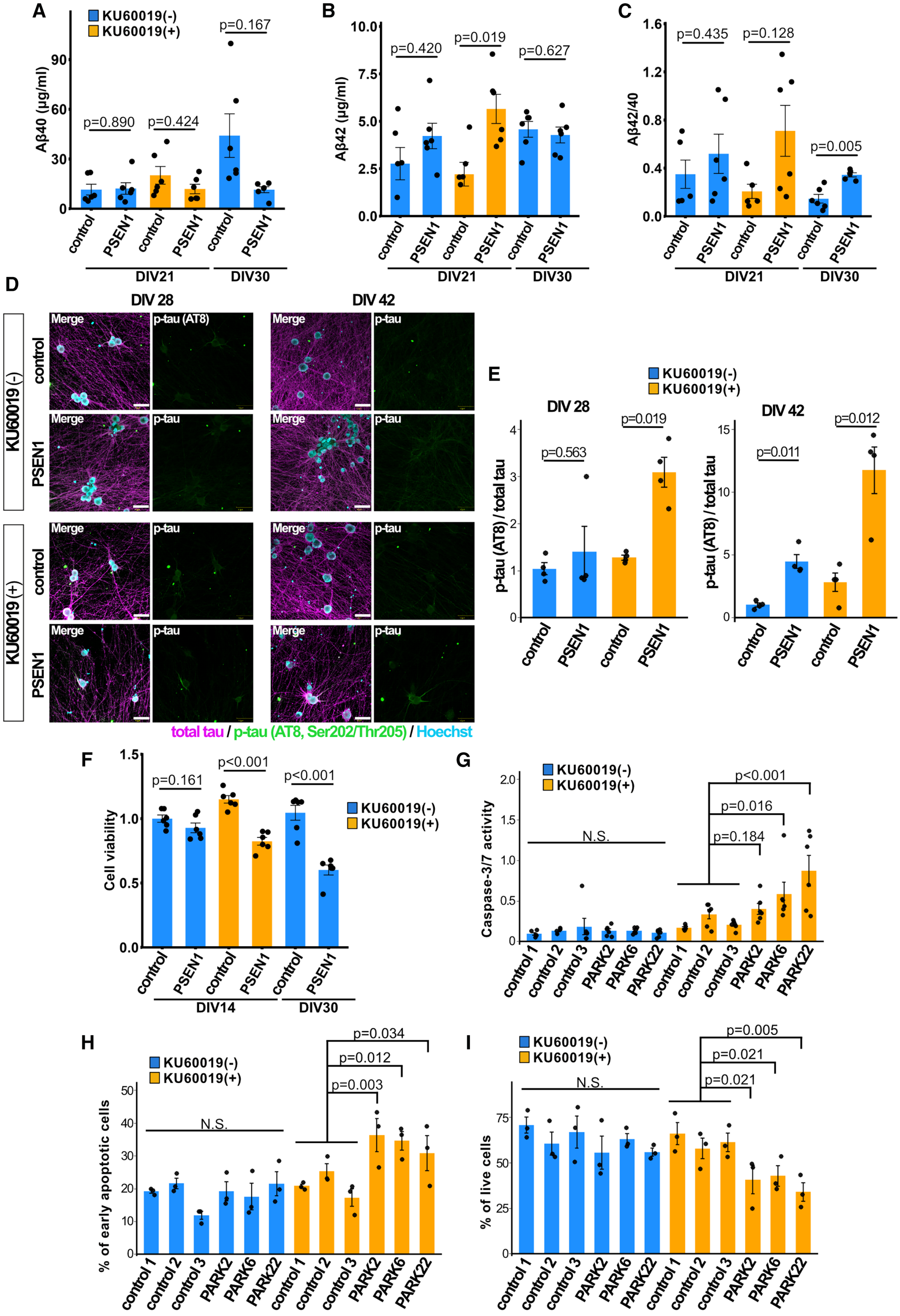

(legend on next page)

pro-survival signaling and BCL-2-mediated anti-apoptotic pathways.

KU60019 treatment accelerates phenotypic recapitulation in hiPSC models of neurodegenerative diseases

Given the pro-aging effects of KU60019, we hypothesized that KU60019 may accelerate the phenotypic manifestation of neurodegenerative diseases by inducing senescence-associated phenotypic changes in hiPSC-derived neurons. AD, the most common late-onset neurodegenerative disorder, is caused in part by variants in *PSEN1*. To assess whether KU60019 promotes amyloid-related phenotypes in *PSEN1* variant-carrying neurons, we measured Aβ40 and Aβ42 levels and calculated the Aβ42/Aβ40 ratio at DIV21 and DIV30. At DIV21, no significant genotype-dependent differences were observed without KU60019 treatment. In contrast, under KU60019 treatment, PSEN1-variant neurons showed significantly higher Aβ42 levels than control neurons, whereas Aβ40 levels remained unchanged. The Aβ42/Aβ40 ratio also increased in the same direction, although this difference did not reach statistical significance after multiple-comparison correction (Figures 7A–7C). Previous studies have reported increased p-tau/total tau ratios and enhanced neuronal vulnerability in AD models (Bassil et al., 2021). We next examined tau-related phenotypes in *PSEN1* variant-carrying and control hiPSC-derived neurons. At DIV28, the p-Ser202/Thr205 tau/total tau ratio did not differ between PSEN1-variant and control neurons without KU60019 treatment, whereas KU60019 treatment resulted in a significantly higher ratio in PSEN1-variant neurons (Figures 7D and 7E). At DIV42, PSEN1-variant neurons exhibited increased p-tau/total tau ratios both with and without KU60019 treatment, with a more pronounced difference observed following KU60019 treatment (Figures 7D and 7E). In addition, without KU60019 treatment, no significant difference in cell viability was observed between PSEN1-variant and control neurons at day 14, whereas a significant reduction in viability was detected in PSEN1-variant neurons at day 30. In contrast, KU60019 treatment led to a significant decrease in the viability of PSEN1-variant neurons as early as day 14 (Figure 7F).

We next examined the effects of KU60019 in hiPSC-derived neurons from patients with PD, another major neurodegenerative disorder characterized by the progressive loss of dopaminergic neurons in the ventral midbrain. Among inherited forms of PD, PARK2, PARK6, and PARK22 typically present after adolescence but earlier than sporadic PD, with disease severity worsening with age. Previous studies have reported increased caspase-3 activation in dopaminergic neurons derived from patients with these forms of PD (Ikeda et al., 2019; Yamaguchi et al., 2020). In this hiPSC-derived neuronal model, no significant difference in caspase-3/7 activity was observed between control and PD-derived dopaminergic neurons at 14 days after the onset of terminal differentiation (Figure 7G). However, KU60019 treatment significantly increased caspase-3/7 activity in PD-derived neurons compared with controls (Figure 7G). Consistently, in the absence of KU60019 treatment, no significant difference in early apoptotic cells (Annexin V-positive and 7-AAD-negative) was observed between control and PD groups at day 14 (Figure 7H). In contrast, KU60019 treatment significantly increased the proportion of early apoptotic cells in PD-derived dopaminergic neurons (Figure 7H). Moreover, in the absence of KU60019 treatment, the percentage of viable cells (Annexin V-negative and 7-AAD-negative) did not differ between control and PD groups at day 14. In contrast, KU60019 treatment significantly reduced the proportion of viable cells in PD-derived neurons (Figure 7I).

Taken together, these results indicate that KU60019 treatment accelerates phenotypic recapitulation in hiPSC-derived models of late-onset neurodegenerative diseases.

DISCUSSION

In this study, we identified KU60019, an ATM kinase inhibitor, as a compound that promotes the maturation of

Figure 7. KU60019 accelerates the recapitulation of late-onset neurodegenerative disease-associated phenotypes in hiPSC-derived neurons

(A–C) Aβ40 levels (A), Aβ42 levels (B), and Aβ42/Aβ40 ratios (C) in control and PSEN1-variant hiPSC-derived neurons at DIV21 and DIV30, measured by ELISA. $n = 5$–6 independent experiments.

(D) Representative images of total tau and phosphorylated tau (p-tau; AT8, Ser202/Thr205) immunostaining in untreated (upper images) and KU60019-treated (lower images) control and PSEN1-variant hiPSC-derived neurons. Scale bars, 50 μm.

(E) Quantification of p-tau/total tau fluorescence intensity ratios at DIV28 and DIV42. $n = 4$ independent experiments.

(F) Cell viability of control and PSEN1-variant hiPSC-derived neurons. $n = 6$ independent experiments.

(G–I) Caspase-3/7 activity (G), early apoptosis (Annexin V^+/7-AAD^- cells) (H), and cell viability (Annexin V^-/7-AAD^- cells) (I) in three independent control hiPSC-derived neuronal lines and PD hiPSC-derived neurons (PARK2, PARK6, and PARK22). $n = 3$ independent experiments. Data are shown as mean ± SEM. p values were calculated using planned Welch's t tests with Holm correction for multiple comparisons (A–C, E, and F). For (G–I), planned contrasts were used to compare each disease line with the mean of control lines within each set (Welch/linear model-based contrasts with Holm correction).

hiPSC-derived neurons while simultaneously inducing diverse senescence-associated phenotypes. Importantly, KU60019 treatment facilitated the manifestation of disease-relevant phenotypes in hiPSC-derived models of AD and PD within a relatively short culture period. These findings suggest that KU60019 creates a cellular state in which maturation-associated neuronal features and aging-associated phenotypes emerge in parallel, thereby enhancing the utility of hiPSC-based models of late-onset neurodegenerative diseases.

Premature aging syndromes are frequently caused by defects in genes responsible for maintaining genomic stability (Schumacher et al., 2021). Ataxia-telangiectasia, caused by mutations in ATM, is a representative disorder characterized by genomic instability and premature aging phenotypes (Barlow et al., 1996; Shiloh and Lederman, 2017). In addition, ATM deficiency has been associated with multiple senescence-related abnormalities, including increased DNA damage, altered nuclear morphology, decreased NAD levels, and neurodegeneration (Barlow et al., 1996; Dong et al., 2022; Fang et al., 2016). Accordingly, patient-derived ATM-mutant brain organoids and mature neurons have been reported to show senescence-associated changes, including activation of senescence-related transcriptional programs, increased SA-βGal staining, and increased p16/p21 expression (Leeson et al., 2024). Consistent with these observations, KU60019-treated cells exhibited similar features, suggesting that ATM inhibition contributes to the induction of an aging-like cellular state.

Aging hallmarks are highly interconnected, and DNA damage plays a central role in their regulation (Hou et al., 2019; López-Otín et al., 2023). ATM is a key regulator of the DDR, and pharmacological perturbation of DNA repair pathways has been shown to accelerate aging-associated phenotypes in hiPSC-derived neurons (Fathi et al., 2022; Saurat et al., 2024). However, not all DNA damage-inducing approaches are equally suitable for modeling cellular senescence. Some compounds, such as MLN4924 or O151, require additional conditions or exhibit broad and non-specific effects. In contrast, KU60019 induced coordinated alterations across multiple pathways, including cell-cycle regulation, p53 signaling, and diverse DNA repair pathways. This broad yet structured perturbation likely reflects the central role of ATM in integrating DNA damage signaling, replication stress responses, and cell-cycle control. Therefore, KU60019-mediated senescence is likely driven by the combined impairment of these interconnected mechanisms. Consistent with this notion, KU60019-treated cells exhibited a broad spectrum of senescence-associated phenotypes, including increased SA-βGal activity, autophagic abnormalities, and alterations in protein digestion and absorption pathways, some of which are not directly attributable to canonical ATM signaling. Taken together, these findings suggest that KU60019 can recapitulate multifaceted senescence-like phenotypes.

An important conceptual point is that neuronal maturation and cellular senescence are distinct biological processes. Cellular senescence is generally defined as a stress-associated state characterized by cell-cycle arrest and distinct phenotypic changes (Gorgoulis et al., 2019; López-Otín et al., 2023), whereas neuronal maturation reflects developmental progression toward functional competence. Nevertheless, in our experimental system, these processes coexisted. KU60019 was initially identified as a maturation-promoting compound, as evidenced by increased synapsin expression, enhanced axonal elongation, and the emergence of spontaneous neuronal activity within a relatively short time frame. At the same time, it robustly induced senescence-associated phenotypes. These observations suggest that KU60019 does not equate maturation with senescence but rather induces a senescence-associated cellular context in which maturation-associated neuronal features can emerge more rapidly.

This interpretation is consistent with previous reports linking senescence-related programs to developmental processes. Cellular senescence has been described as a programmed process during embryonic development, contributing to tissue patterning and morphogenesis (Di Micco et al., 2021). In addition, DNA damage-induced senescence in neural stem cells has been shown to promote differentiation while reducing stemness (Schneider et al., 2013). Thus, ATM signaling may intersect with differentiation programs in the nervous system. In this context, KU60019 treatment during neuronal differentiation may accelerate the transition from progenitor states to functionally mature neurons.

Our senolytic screening further suggested that KU60019-induced senescent cells depend on HSP90-associated survival pathways. HSP90 stabilizes multiple client proteins involved in DDR and pro-survival signaling, including ATM, ATR, and AKT (Karayazi Atici et al., 2018; Pennisi et al., 2015). Consistent with previous studies, HSP90 inhibition by 17DMAG induced cell death in KU60019-treated cells. However, inhibition of the PI3K-AKT-mTOR pathway alone by quercetin and fisetin, or by rapamycin, failed to reproduce this effect, suggesting that the cell death-inducing (senolytic) activity of HSP90 inhibition cannot be explained solely by suppression of this pathway.

Importantly, ATM inhibition itself appeared to induce a BCL-2-dependent survival state. KU60019 increased BCL-2 expression and reduced the BAX/BCL-2 ratio, and this phenotype was functionally supported by sensitivity to ABT-737. Such anti-apoptotic signaling is a well-recognized feature of senescent cells (Yosef et al., 2016). In this context, HSP90 inhibition likely eliminates these cells by simultaneously disrupting multiple survival pathways,

including NF-κB and AKT signaling. Notably, apoptosis was induced despite sustained BCL-2 expression, indicating that mitochondrial protection alone is insufficient when upstream survival signaling is compromised. Taken together, these findings suggest that KU60019 recapitulates key survival features of senescent cells, whereas HSP90 inhibition exploits these vulnerabilities.

Several limitations should be considered. First, the relationship between KU60019-induced phenotypes and physiological aging *in vitro* remains unclear. Our results demonstrate the induction of an aging-like cellular state under specific experimental conditions rather than establishing a direct equivalence with neural aging. Second, although the effects of KU60019 were observed across multiple cell types, their generalizability remains to be determined. Third, the effects of KU60019 appear to be context-dependent. In contrast to our findings, Kang et al. reported that KU60019 alleviated senescence phenotypes in late-passage human neonatal dermal fibroblasts, suggesting that differences in cellular state, treatment duration, and drug concentration may critically influence the outcome of ATM inhibition (Kang et al., 2017). Finally, we did not directly assess cell-cycle dynamics, and the mechanistic relationship between senescence and neuronal maturation remains unresolved.

In conclusion, KU60019 induces a senescence-associated cellular state while accelerating maturation-associated neuronal features in hiPSC-derived neurons, thereby enabling more rapid manifestation of disease-relevant phenotypes. By promoting both aging-related and functional neuronal characteristics, KU60019 provides a simple, chemically defined, and practical approach to enhance phenotypic recapitulation in hiPSC models of late-onset neurodegenerative diseases.

RESOURCE AVAILABILITY

Lead contact

Further information and requests for resources and reagents should be directed to and will be fulfilled by Wado Akamatsu (awado@juntendo.ac.jp).

Materials availability

This study did not generate new unique reagents. Information on the hiPSC and fibroblast lines used in this study is provided in the key resources table.

Data and code availability

The data supporting the findings of this study are available within the article and its supplemental information. The RNA-seq data have been deposited in the DDBJ Sequence Read Archive (accession number: PRJDB17588). This study did not generate new code.

ACKNOWLEDGMENTS

This work was supported by the Strategic Research Platform Formation Project for Private Universities from the Ministry of Education, Culture, Sports, Science and Technology of Japan (MEXT; S1411007); the Practical Research Project for Rare/Intractable Diseases from the Japan Agency for Medical Research and Development (AMED; JP17ek0109244 to K.I., N.H., and W.A.); the Acceleration Program of R&D and Implementation for Regenerative Medicine and Cell and Gene Therapy from AMED (JP25bm1423015 to K.I. and W.A.); the Basic Research Program for Drug Discovery Promotion (GAPFREE) from AMED (JP22ak0101112 to K.I., N.H., and W.A., and JP25ak0101236 to N.H. and W.A.); AMED-CREST from AMED (JP24gm1310003 to S.O. and W.A.); JSPS KAKENHI (20K07873 to K.I., 19K16930 to T.S., and 17H05706 to W.A.); and a Grant-in-Aid for Special Research in Subsidies for ordinary expenses of private schools from the Promotion and Mutual Aid Corporation for Private Schools of Japan. We thank Editage (www.editage.com) for English language editing.

AUTHOR CONTRIBUTIONS

Conceptualization, T.S., K.I., and W.A.; methodology, K.I., T.S., N.K., H.T., A.K.S., K.N., K.B., S.O., N.H., H.O., and W.A.; investigation, K.I., T.S., T.H., N.K., S.M., H.T., A.K.S., and K.N.; writing – original draft, K.I., T.S., and W.A.; writing – review and editing, K.I., T.S., T.H., N.K., S.M., A.Y., H.T., A.K.S., K.N., K.B., S.O., N.H., H.O., and W.A.; visualization, K.I., T.S., T.H., N.K., A.Y., H.T., and A.K.S.; supervision, K.I. and W.A.; project administration, T.S., K.I., and W.A.; funding acquisition, K.I., T.S., S.O., N.H., H.O., and W.A.

DECLARATION OF INTERESTS

The authors declare no competing interests.

STAR★METHODS

Detailed methods are provided in the online version of this paper and include the following:

- KEY RESOURCES TABLE
- EXPERIMENTAL MODEL AND STUDY PARTICIPANT DETAILS
  - Culture of hiPSCs
  - Culture of human dermal fibroblasts
  - Culture of SH-SY5Y cells
  - Human hiPSC-derived neurons for PSEN1 assays
- METHOD DETAILS
  - Differentiation of hiPSCs into dopaminergic neurons
  - Compound screening
  - Senolytic drug treatment
  - Immunostaining and quantification
  - SA-βGal staining and quantification
  - Sparse labeling and neurite length measurement
  - Whole-cell patch-clamp recordings
  - Western blotting
  - Measurement of total NAD/NADH
  - Cytokine array
  - Quantitative reverse transcription PCR and RNA-seq analysis
  - PSEN1-variant hiPSC-derived neurons
  - Measurement of Aβ40 and Aβ42 by ELISA

- Cell viability and caspase-3/7 assays
- Annexin V/7-AAD apoptosis assay
- QUANTIFICATION AND STATISTICAL ANALYSIS
  - Statistical analysis

SUPPLEMENTAL INFORMATION

Supplemental information can be found online at https://doi.org/10.1016/j.stemcr.2026.102956.

Received: September 9, 2024
Revised: May 12, 2026
Accepted: May 13, 2026
Published: June 11, 2026

REFERENCES

Alessio, N., Aprile, D., Cappabianca, S., Peluso, G., Di Bernardo, G., and Galderisi, U. (2021). Different Stages of Quiescence, Senescence, and Cell Stress Identified by Molecular Algorithm Based on the Expression of Ki67, RPS6, and Beta-Galactosidase Activity. Int. J. Mol. Sci. *22*, 3102. https://doi.org/10.3390/ijms22063102.

Barlow, C., Hirotsune, S., Paylor, R., Liyanage, M., Eckhaus, M., Collins, F., Shiloh, Y., Crawley, J.N., Ried, T., Tagle, D., and Wynshaw-Boris, A. (1996). Atm-deficient mice: a paradigm of ataxia telangiectasia. Cell *86*, 159–171. https://doi.org/10.1016/s0092-8674(00)80086-0.

Bassil, R., Shields, K., Granger, K., Zein, I., Ng, S., and Chih, B. (2021). Improved modeling of human AD with an automated culturing platform for iPSC neurons, astrocytes and microglia. Nat. Commun. *12*, 5220. https://doi.org/10.1038/s41467-021-25344-6.

Basu, A. (2022). The interplay between apoptosis and cellular senescence: Bcl-2 family proteins as targets for cancer therapy. Pharmacol. Ther. *230*, 107943. https://doi.org/10.1016/j.pharmthera.2021.107943.

de Luzy, I.R., Lee, M.K., Mobley, W.C., and Studer, L. (2024). Lessons from inducible pluripotent stem cell models on neuronal senescence in aging and neurodegeneration. Nat. Aging *4*, 309–318. https://doi.org/10.1038/s43587-024-00586-3.

Di Micco, R., Krizhanovsky, V., Baker, D., and d'Adda di Fagagna, F. (2021). Cellular senescence in ageing: from mechanisms to therapeutic opportunities. Nat. Rev. Mol. Cell Biol. *22*, 75–95. https://doi.org/10.1038/s41580-020-00314-w.

Dong, C., Wang, X., Sun, L., Zhu, L., Yang, D., Gao, S., Zhang, W., Ling, B., Liang, A., Gao, Z., and Xu, J. (2022). ATM modulates subventricular zone neural stem cell maintenance and senescence through Notch signaling pathway. Stem Cell Res. *58*, 102618. https://doi.org/10.1016/j.scr.2021.102618.

Fang, E.F., Kassahun, H., Croteau, D.L., Scheibye-Knudsen, M., Marosi, K., Lu, H., Shamanna, R.A., Kalyanasundaram, S., Bollineni, R.C., Wilson, M.A., et al. (2016). NAD(+) Replenishment Improves Lifespan and Healthspan in Ataxia Telangiectasia Models via Mitophagy and DNA Repair. Cell Metab. *24*, 566–581. https://doi.org/10.1016/j.cmet.2016.09.004.

Fathi, A., Mathivanan, S., Kong, L., Petersen, A.J., Harder, C.R.K., Block, J., Miller, J.M., Bhattacharyya, A., Wang, D., and Zhang, S.C. (2022). Chemically induced senescence in human stem cell-derived neurons promotes phenotypic presentation of neurodegeneration. Aging Cell *21*, e13541. https://doi.org/10.1111/acel.13541.

Freund, A., Laberge, R.M., Demaria, M., and Campisi, J. (2012). Lamin B1 loss is a senescence-associated biomarker. Mol. Biol. Cell *23*, 2066–2075. https://doi.org/10.1091/mbc.E11-10-0884.

Fuhrmann-Stroissnigg, H., Ling, Y.Y., Zhao, J., McGowan, S.J., Zhu, Y., Brooks, R.W., Grassi, D., Gregg, S.Q., Stripay, J.L., Dorronsoro, A., et al. (2017). Identification of HSP90 inhibitors as a novel class of senolytics. Nat. Commun. *8*, 422. https://doi.org/10.1038/s41467-017-00314-z.

Gantner, C.W., Cota-Coronado, A., Thompson, L.H., and Parish, C.L. (2020). An Optimized Protocol for the Generation of Midbrain Dopamine Neurons under Defined Conditions. STAR Protoc. *1*, 100065. https://doi.org/10.1016/j.xpro.2020.100065.

Gorgoulis, V., Adams, P.D., Alimonti, A., Bennett, D.C., Bischof, O., Bishop, C., Campisi, J., Collado, M., Evangelou, K., Ferbeyre, G., et al. (2019). Cellular Senescence: Defining a Path Forward. Cell *179*, 813–827. https://doi.org/10.1016/j.cell.2019.10.005.

Hoashi, Y., Okamoto, S., Abe, Y., Matsumoto, T., Tanaka, J., Yoshida, Y., Imaizumi, K., Mishima, K., Akamatsu, W., Okano, H., and Baba, K. (2017). Generation of neural cells using iPSCs from sleep bruxism patients with 5-HT2A polymorphism. J. Prosthodont. Res. *61*, 242–250. https://doi.org/10.1016/j.jpor.2016.11.003.

Hou, Y., Dan, X., Babbar, M., Wei, Y., Hasselbalch, S.G., Croteau, D.L., and Bohr, V.A. (2019). Ageing as a risk factor for neurodegenerative disease. Nat. Rev. Neurol. *15*, 565–581. https://doi.org/10.1038/s41582-019-0244-7.

Ikeda, A., Nishioka, K., Meng, H., Takanashi, M., Hasegawa, I., Inoshita, T., Shiba-Fukushima, K., Li, Y., Yoshino, H., Mori, A., et al. (2019). Mutations in CHCHD2 cause α-synuclein aggregation. Hum. Mol. Genet. *28*, 3895–3911. https://doi.org/10.1093/hmg/ddz241.

Imaizumi, Y., Okada, Y., Akamatsu, W., Koike, M., Kuzumaki, N., Hayakawa, H., Nihira, T., Kobayashi, T., Ohyama, M., Sato, S., et al. (2012). Mitochondrial dysfunction associated with increased oxidative stress and α-synuclein accumulation in PARK2 iPSC-derived neurons and postmortem brain tissue. Mol. Brain *5*, 35. https://doi.org/10.1186/1756-6606-5-35.

Ishikawa, K.I., Okuzumi, A., Yoshino, H., Hattori, N., and Akamatsu, W. (2024a). Generation of hiPSCs (JUCGRMi003-A) from a patient with Parkinson's disease with PARK2 mutation. Stem Cell Res. *76*, 103323. https://doi.org/10.1016/j.scr.2024.103323.

Ishikawa, K.I., Shiga, T., Funayama, M., Hattori, N., and Akamatsu, W. (2024b). Generation of a control iPS cell line (JUCGRMi005-A) with no abnormalities in Parkinson's disease-related genes. Stem Cell Res. *74*, 103271. https://doi.org/10.1016/j.scr.2023.103271.

Ishikawa, K.I., Shiga, T., Funayama, M., Hattori, N., and Akamatsu, W. (2024c). Generation of a control iPS cell line (JUCGRMi006-A) with no abnormalities in Parkinson's disease-related genes. Stem Cell Res. *74*, 103270. https://doi.org/10.1016/j.scr.2023.103270.

Ivanov, A., Pawlikowski, J., Manoharan, I., van Tuyn, J., Nelson, D.M., Rai, T.S., Shah, P.P., Hewitt, G., Korolchuk, V.I., Passos, J.F., et al. (2013). Lysosome-mediated processing of chromatin in

senescence. J. Cell Biol. *202*, 129–143. https://doi.org/10.1083/jcb.201212110.

Jothi, D., and Kulka, L.A.M. (2024). Strategies for modeling aging and age-related diseases. NPJ Aging *10*, 32. https://doi.org/10.1038/s41514-024-00161-5.

Kang, H.T., Park, J.T., Choi, K., Kim, Y., Choi, H.J.C., Jung, C.W., Lee, Y.S., and Park, S.C. (2017). Chemical screening identifies ATM as a target for alleviating senescence. Nat. Chem. Biol. *13*, 616–623. https://doi.org/10.1038/nchembio.2342.

Karayazi Atici, Ö., Urbanska, A., Gopinathan, S.G., Boutillon, F., Goffin, V., and Shemanko, C.S. (2018). ATM Is Required for the Prolactin-Induced HSP90-Mediated Increase in Cellular Viability and Clonogenic Growth After DNA Damage. Endocrinology *159*, 907–930. https://doi.org/10.1210/en.2017-00652.

Kim, D., Langmead, B., and Salzberg, S.L. (2015). HISAT: a fast spliced aligner with low memory requirements. Nat. Methods *12*, 357–360. https://doi.org/10.1038/nmeth.3317.

Kim, J.G., Lee, S.C., Kim, O.H., Kim, K.H., Song, K.Y., Lee, S.K., Choi, B.J., Jeong, W., and Kim, S.J. (2017). HSP90 inhibitor 17-DMAG exerts anticancer effects against gastric cancer cells principally by altering oxidant-antioxidant balance. Oncotarget *8*, 56473–56489. https://doi.org/10.18632/oncotarget.17007.

Kondo, T., Imamura, K., Funayama, M., Tsukita, K., Miyake, M., Ohta, A., Woltjen, K., Nakagawa, M., Asada, T., Arai, T., et al. (2017). iPSC-Based Compound Screening and In Vitro Trials Identify a Synergistic Anti-amyloid beta Combination for Alzheimer's Disease. Cell Rep. *21*, 2304–2312. https://doi.org/10.1016/j.celrep.2017.10.109.

Laberge, R.M., Sun, Y., Orjalo, A.V., Patil, C.K., Freund, A., Zhou, L., Curran, S.C., Davalos, A.R., Wilson-Edell, K.A., Liu, S., et al. (2015). MTOR regulates the pro-tumorigenic senescence-associated secretory phenotype by promoting IL1A translation. Nat. Cell Biol. *17*, 1049–1061. https://doi.org/10.1038/ncb3195.

Leeson, H.C., Aguado, J., Gómez-Inclán, C., Chaggar, H.K., Fard, A.T., Hunter, Z., Lavin, M.F., Mackay-Sim, A., and Wolvetang, E.J. (2024). Ataxia Telangiectasia patient-derived neuronal and brain organoid models reveal mitochondrial dysfunction and oxidative stress. Neurobiol. Dis. *199*, 106562. https://doi.org/10.1016/j.nbd.2024.106562.

Li, H., Handsaker, B., Wysoker, A., Fennell, T., Ruan, J., Homer, N., Marth, G., Abecasis, G., and Durbin, R.; 1000 Genome Project Data Processing Subgroup (2009). The Sequence Alignment/Map format and SAMtools. Bioinformatics *25*, 2078–2079. https://doi.org/10.1093/bioinformatics/btp352.

Liao, Y., Smyth, G.K., and Shi, W. (2014). featureCounts: an efficient general purpose program for assigning sequence reads to genomic features. Bioinformatics *30*, 923–930. https://doi.org/10.1093/bioinformatics/btt656.

López-Otín, C., Blasco, M.A., Partridge, L., Serrano, M., and Kroemer, G. (2023). Hallmarks of aging: An expanding universe. Cell *186*, 243–278. https://doi.org/10.1016/j.cell.2022.11.001.

Matsumoto, T., Fujimori, K., Andoh-Noda, T., Ando, T., Kuzumaki, N., Toyoshima, M., Tada, H., Imaizumi, K., Ishikawa, M., Yamaguchi, R., et al. (2016). Functional Neurons Generated from T Cell-Derived Induced Pluripotent Stem Cells for Neurological Disease Modeling. Stem Cell Rep. *6*, 422–435. https://doi.org/10.1016/j.stemcr.2016.01.010.

Mertens, J., Paquola, A.C.M., Ku, M., Hatch, E., Böhnke, L., Ladjevardi, S., McGrath, S., Campbell, B., Lee, H., Herdy, J.R., et al. (2015). Directly Reprogrammed Human Neurons Retain Aging-Associated Transcriptomic Signatures and Reveal Age-Related Nucleocytoplasmic Defects. Cell Stem Cell *17*, 705–718. https://doi.org/10.1016/j.stem.2015.09.001.

Miller, J.D., Ganat, Y.M., Kishinevsky, S., Bowman, R.L., Liu, B., Tu, E.Y., Mandal, P.K., Vera, E., Shim, J.W., Kriks, S., et al. (2013). Human iPSC-based modeling of late-onset disease via progerin-induced aging. Cell Stem Cell *13*, 691–705. https://doi.org/10.1016/j.stem.2013.11.006.

Moiseeva, O., Deschênes-Simard, X., St-Germain, E., Igelmann, S., Huot, G., Cadar, A.E., Bourdeau, V., Pollak, M.N., and Ferbeyre, G. (2013). Metformin inhibits the senescence-associated secretory phenotype by interfering with IKK/NF-κB activation. Aging Cell *12*, 489–498. https://doi.org/10.1111/acel.12075.

Nakamura, R., Nonaka, R., Oyama, G., Jo, T., Kamo, H., Nuermaimaiti, M., Akamatsu, W., Ishikawa, K.I., and Hattori, N. (2023). A defined method for differentiating human iPSCs into midbrain dopaminergic progenitors that safely restore motor deficits in Parkinson's disease. Front. Neurosci. *17*, 1202027. https://doi.org/10.3389/fnins.2023.1202027.

Odawara, A., Katoh, H., Matsuda, N., and Suzuki, I. (2016). Physiological maturation and drug responses of human induced pluripotent stem cell-derived cortical neuronal networks in long-term culture. Sci. Rep. *6*, 26181. https://doi.org/10.1038/srep26181.

Okano, H., and Morimoto, S. (2022). iPSC-based disease modeling and drug discovery in cardinal neurodegenerative disorders. Cell Stem Cell *29*, 189–208. https://doi.org/10.1016/j.stem.2022.01.007.

Osabe, T., Shimizu, K., and Kadota, K. (2019). Accurate Classification of Differential Expression Patterns in a Bayesian Framework With Robust Normalization for Multi-Group RNA-Seq Count Data. Bioinform. Biol. Insights *13*, 1177932219860817. https://doi.org/10.1177/1177932219860817.

Ozsvari, B., Nuttall, J.R., Sotgia, F., and Lisanti, M.P. (2018). Azithromycin and Roxithromycin define a new family of "senolytic" drugs that target senescent human fibroblasts. Aging (Albany NY) *10*, 3294–3307. https://doi.org/10.18632/aging.101633.

Pennisi, R., Ascenzi, P., and di Masi, A. (2015). Hsp90: A New Player in DNA Repair? Biomolecules *5*, 2589–2618. https://doi.org/10.3390/biom5042589.

Riessland, M., Kolisnyk, B., Kim, T.W., Cheng, J., Ni, J., Pearson, J.A., Park, E.J., Dam, K., Acehan, D., Ramos-Espiritu, L.S., et al. (2019). Loss of SATB1 Induces p21-Dependent Cellular Senescence in Post-mitotic Dopaminergic Neurons. Cell Stem Cell *25*, 514–530.e8. https://doi.org/10.1016/j.stem.2019.08.013.

Russo, T., Kolisnyk, B., Aswathy, B.S., Plessis-Belair, J., Kim, T.W., Martin, J., Ni, J., Pearson, J.A., Park, E.J., Sher, R.B., et al. (2024). The SATB1-MIR22-GBA axis mediates glucocerebroside accumulation inducing a cellular senescence-like phenotype in dopaminergic neurons. Aging Cell *23*, e14077. https://doi.org/10.1111/acel.14077.

Saurat, N., Minotti, A.P., Rahman, M.T., Sikder, T., Zhang, C., Cornacchia, D., Jungverdorben, J., Ciceri, G., Betel, D., and Studer, L. (2024). Genome-wide CRISPR screen identifies neddylation as a regulator of neuronal aging and AD neurodegeneration. Cell Stem Cell *31*, 1162–1174.e9. https://doi.org/10.1016/j.stem.2024.06.001.

Schneider, L., Pellegatta, S., Favaro, R., Pisati, F., Roncaglia, P., Testa, G., Nicolis, S.K., Finocchiaro, G., and d'Adda di Fagagna, F. (2013). DNA damage in mammalian neural stem cells leads to astrocytic differentiation mediated by BMP2 signaling through JAK-STAT. Stem Cell Rep. *1*, 123–138. https://doi.org/10.1016/j.stemcr.2013.06.004.

Schumacher, B., Pothof, J., Vijg, J., and Hoeijmakers, J.H.J. (2021). The central role of DNA damage in the ageing process. Nature *592*, 695–703. https://doi.org/10.1038/s41586-021-03307-7.

Shiba-Fukushima, K., Ishikawa, K.I., Inoshita, T., Izawa, N., Takanashi, M., Sato, S., Onodera, O., Akamatsu, W., Okano, H., Imai, Y., and Hattori, N. (2017). Evidence that phosphorylated ubiquitin signaling is involved in the etiology of Parkinson's disease. Hum. Mol. Genet. *26*, 3172–3185. https://doi.org/10.1093/hmg/ddx201.

Shiloh, Y., and Lederman, H.M. (2017). Ataxia-telangiectasia (A-T): An emerging dimension of premature ageing. Ageing Res. Rev. *33*, 76–88. https://doi.org/10.1016/j.arr.2016.05.002.

Sun, J., Nishiyama, T., Shimizu, K., and Kadota, K. (2013). TCC: an R package for comparing tag count data with robust normalization strategies. BMC Bioinf. *14*, 219. https://doi.org/10.1186/1471-2105-14-219.

Takahashi, K., Tanabe, K., Ohnuki, M., Narita, M., Ichisaka, T., Tomoda, K., and Yamanaka, S. (2007). Induction of pluripotent stem cells from adult human fibroblasts by defined factors. Cell *131*, 861–872. https://doi.org/10.1016/j.cell.2007.11.019.

Trujillo, C.A., Gao, R., Negraes, P.D., Gu, J., Buchanan, J., Preissl, S., Wang, A., Wu, W., Haddad, G.G., Chaim, I.A., et al. (2019). Complex Oscillatory Waves Emerging from Cortical Organoids Model Early Human Brain Network Development. Cell Stem Cell *25*, 558–569.e7. https://doi.org/10.1016/j.stem.2019.08.002.

Vera, E., Bosco, N., and Studer, L. (2016). Generating Late-Onset Human iPSC-Based Disease Models by Inducing Neuronal Age-Related Phenotypes through Telomerase Manipulation. Cell Rep. *17*, 1184–1192. https://doi.org/10.1016/j.celrep.2016.09.062.

Wu, T., Hu, E., Xu, S., Chen, M., Guo, P., Dai, Z., Feng, T., Zhou, L., Tang, W., Zhan, L., et al. (2021). clusterProfiler 4.0: A universal enrichment tool for interpreting omics data. Innovation *2*, 100141. https://doi.org/10.1016/j.xinn.2021.100141.

Yamaguchi, A., Ishikawa, K.I., Inoshita, T., Shiba-Fukushima, K., Saiki, S., Hatano, T., Mori, A., Oji, Y., Okuzumi, A., Li, Y., et al. (2020). Identifying Therapeutic Agents for Amelioration of Mitochondrial Clearance Disorder in Neurons of Familial Parkinson Disease. Stem Cell Rep. *14*, 1060–1075. https://doi.org/10.1016/j.stemcr.2020.04.011.

Yang, N.C., and Hu, M.L. (2005). The limitations and validities of senescence associated-beta-galactosidase activity as an aging marker for human foreskin fibroblast Hs68 cells. Exp. Gerontol. *40*, 813–819. https://doi.org/10.1016/j.exger.2005.07.011.

Yosef, R., Pilpel, N., Tokarsky-Amiel, R., Biran, A., Ovadya, Y., Cohen, S., Vadai, E., Dassa, L., Shahar, E., Condiotti, R., et al. (2016). Directed elimination of senescent cells by inhibition of BCL-W and BCL-XL. Nat. Commun. *7*, 11190. https://doi.org/10.1038/ncomms11190.

Yousefzadeh, M.J., Zhu, Y., McGowan, S.J., Angelini, L., Fuhrmann-Stroissnigg, H., Xu, M., Ling, Y.Y., Melos, K.I., Pirtskhalava, T., Inman, C.L., et al. (2018). Fisetin is a senotherapeutic that extends health and lifespan. EBioMedicine *36*, 18–28. https://doi.org/10.1016/j.ebiom.2018.09.015.

Zhu, Y., Tchkonia, T., Pirtskhalava, T., Gower, A.C., Ding, H., Giorgadze, N., Palmer, A.K., Ikeno, Y., Hubbard, G.B., Lenburg, M., et al. (2015). The Achilles' heel of senescent cells: from transcriptome to senolytic drugs. Aging Cell *14*, 644–658. https://doi.org/10.1111/acel.12344.

Zorin, V., Grekhova, A., Pustovalova, M., Zorina, A., Smetanina, N., Vorobyeva, N., Kopnin, P., Gilmutdinova, I., Moskalev, A., Osipov, A.N., and Leonov, S. (2019). Spontaneous γH2AX foci in human dermal fibroblasts in relation to proliferation activity and aging. Aging (Albany NY) *11*, 4536–4546. https://doi.org/10.18632/aging.102067.

STAR★METHODS

KEY RESOURCES TABLE

REAGENT or RESOURCE	SOURCE	IDENTIFIER
**Antibodies**		
4EBP1	Cell Signaling Technology	Cat#9644; RRID:AB_2097841
53BP1	Cell Signaling Technology	Cat#4937; RRID:AB_10694558
AKT (total)	Cell Signaling Technology	Cat#9272; RRID:AB_329827
BAX	Cell Signaling Technology	Cat#2772; RRID:AB_10695870
BCL-2	Santa Cruz Biotechnology	Cat#sc-7382; RRID:AB_626736
β-actin	Abcam	Cat#ab8227; RRID:AB_2305186
β3-tubulin	Sigma-Aldrich	Cat#T8660; RRID:AB_477590
Cleaved caspase-3	Cell Signaling Technology	Cat#9661; RRID:AB_2341188
FOXA2	R&D Systems	Cat#AF2400; RRID:AB_2294104
GSK3β	Cell Signaling Technology	Cat#12456; RRID:AB_2636978
H3K27me3	Active Motif	Cat#39536; RRID:AB_2793247
H3K9ac	Active Motif	Cat#39585; RRID:AB_2793268
Histone H3	Cell Signaling Technology	Cat#4499; RRID:AB_10544537
HSP90α	Proteintech	Cat#60318-1-Ig; RRID:AB_2881429
HSP90β	Cell Signaling Technology	Cat#5087; RRID:AB_10548761
IκBα	Cell Signaling Technology	Cat#4814; RRID:AB_390781
IKKα	Cell Signaling Technology	Cat#11930; RRID:AB_2687618
IKKβ	Cell Signaling Technology	Cat#8943; RRID:AB_11024092
Ki67	Santa Cruz Biotechnology	Cat#sc-7846; RRID:AB_2142374
Lamin A/C	Abcam	Cat#ab40567; RRID:AB_775967
Lamin B1	Abcam	Cat#ab133741; RRID:AB_2616597
LC3	Cell Signaling Technology	Cat#3868; RRID:AB_2137707
MAP2	Sigma-Aldrich	Cat#M4403; RRID:AB_477193
NF-κB p65	Cell Signaling Technology	Cat#8242; RRID:AB_10859369
p62	Cell Signaling Technology	Cat#88588; RRID:AB_2800125
p70S6K	Cell Signaling Technology	Cat#2708; RRID:AB_390722
Phospho-4EBP1 (Thr37/46)	Cell Signaling Technology	Cat#2855; RRID:AB_560835
Phospho-AKT (Ser473)	Cell Signaling Technology	Cat#9271; RRID:AB_329825
Phospho-AKT (Thr308)	Cell Signaling Technology	Cat#9275; RRID:AB_329828
Phospho-GSK3β (Ser9)	Cell Signaling Technology	Cat#9336; RRID:AB_331405
Phospho-IκBα	Cell Signaling Technology	Cat#2859; RRID:AB_561111

(*Continued on next page*)

Continued

REAGENT or RESOURCE	SOURCE	IDENTIFIER
Phospho-IKKα/β	Cell Signaling Technology	Cat#2697; RRID:AB_2079382
Phospho-NF-κB p65 (Ser536)	Cell Signaling Technology	Cat#3303; RRID:AB_331284
Phospho-p70S6K (Thr389)	Cell Signaling Technology	Cat#9234; RRID:AB_2269803
Phospho-SGK1 (Ser78)	Cell Signaling Technology	Cat#5599; RRID:AB_10698593
pRPS6	Cell Signaling Technology	Cat#4858; RRID:AB_916156
RIP1	Cell Signaling Technology	Cat#3493; RRID:AB_2305314
SGK1	Cell Signaling Technology	Cat#12103; RRID:AB_2687476
TH	Millipore	Cat#AB152; RRID:AB_390204
total tau (EP2456Y)	Abcam	Cat#ab76128; RRID:AB_1524475
γH2AX	Cell Signaling Technology	Cat#9718; RRID:AB_2118009
γH2AX	Millipore	Cat#05–636; RRID:AB_309864
p-tau (AT8, Ser202/Thr205)	Thermo Fisher Scientific	Cat#MN1020; RRID:AB_223647
Alexa Fluor 488 goat anti-mouse IgG (H + L)	Thermo Fisher Scientific	Cat#A11001; RRID:AB_2534069
Alexa Fluor 488 goat anti-rabbit IgG (H + L)	Thermo Fisher Scientific	Cat#A11008; RRID:AB_143165
Alexa Fluor 555 goat anti-mouse IgG (H + L)	Thermo Fisher Scientific	Cat#A28180; RRID:AB_2536164
Alexa Fluor 594 goat anti-mouse IgG (H + L)	Thermo Fisher Scientific	Cat#A11032; RRID:AB_2534091
Alexa Fluor 594 goat anti-rabbit IgG (H + L)	Thermo Fisher Scientific	Cat#A11012; RRID:AB_2534079
Alexa Fluor 647 goat anti-mouse IgG (H + L)	Thermo Fisher Scientific	Cat#A21236; RRID:AB_2535805
Alexa Fluor 647 goat anti-rabbit IgG (H + L)	Thermo Fisher Scientific	Cat#A21245; RRID:AB_2535813
Peroxidase-AffiniPure goat anti-mouse IgG (H + L)	Jackson ImmunoResearch	Cat#115-035-146; RRID:AB_2307392
Peroxidase-AffiniPure goat anti-rabbit IgG (H + L)	Jackson ImmunoResearch	Cat#111-035-144; RRID:AB_2307391
**Recombinant DNA**		
AAV1-CAG-tdTomato	Addgene	Addgene #59462
Synapsin promoter-driven GFP reporter lentiviral vector	Kind gift from Dr. Hiroyuki Miyoshi, Keio University	N/A
**Chemicals, peptides, and recombinant proteins**		
17-DMAG	Selleck Chemicals	Cat#S1142
2-mercaptoethanol	Sigma-Aldrich	Cat#M3148
ABT-737	Selleck Chemicals	Cat#S1002
Accutase	Nacalai Tesque	Cat#12679-54
Ascorbic acid	Sigma-Aldrich	Cat#A5960
AZD0156	Selleck Chemicals	Cat#S8375
Azithromycin	Selleck Chemicals	Cat#S1835
B27 Plus Supplement	Thermo Fisher Scientific	Cat#A3582801

(*Continued on next page*)

Continued

REAGENT or RESOURCE	SOURCE	IDENTIFIER
B27 Supplement	Thermo Fisher Scientific	Cat#12587-010
Bafilomycin A1	Selleck Chemicals	Cat#S1413
Brain-derived neurotrophic factor (BDNF)	BioLegend	Cat#788904
CHIR99021	Nacalai Tesque	Cat#18764-44
CultureOne	Thermo Fisher Scientific	Cat#A3320201
Dasatinib	Selleck Chemicals	Cat#S1021
DAPT	Sigma-Aldrich	Cat#D5942
Dibutyryl-cAMP	Nacalai Tesque	Cat#11540-61
Dissociation solution for human ES/iPS cells	REPROCELL	Cat#RCHETP002
DMEM	Nacalai Tesque	Cat#08458-16
DMEM/F12	Sigma-Aldrich	Cat#D6421
Doxycycline	Selleck Chemicals	Cat#S4163
ECL Prime Western Blotting Detection Reagent	Cytiva	Cat#RPN2236
Fibroblast growth factor 2 (FGF2)	PeproTech	Cat#100-18B
Fibronectin	Corning	Cat#356008
Fisetin	Selleck Chemicals	Cat#S2298
G418	Roche	Cat#G418-RO
Glial cell line-derived neurotrophic factor (GDNF)	PeproTech	Cat#450-10
Human leukemia inhibitory factor (hLIF)	Nacalai Tesque	Cat#NU0013-1
Inhibitor library	Sigma-Aldrich	Cat#BMINH02MARUN
iMatrix-511	Nippi	Cat#892012
KnockOut Serum Replacement	Thermo Fisher Scientific	Cat#10828-028
KU55933	Selleck Chemicals	Cat#S1092
KU60019	Sigma-Aldrich	Cat#SML1416
KU60019	Selleck Chemicals	Cat#S1570
KBM neural stem cell medium	Kohjin-Bio	Cat#16050100
Laminin	Thermo Fisher Scientific	Cat#23017015
LDN-193189	Selleck Chemicals	Cat#S2618
L-glutamine	Sigma-Aldrich	Cat#G7513
Metformin	Selleck Chemicals	Cat#S5958
MIRIN	Cayman Chemical	Cat#13208
NEBNext Ultra RNA Library Prep Kit for Illumina	New England Biolabs	Cat#E7770
NuPAGE LDS Sample Buffer	Thermo Fisher Scientific	Cat#NP0007

(*Continued on next page*)

Continued

REAGENT or RESOURCE	SOURCE	IDENTIFIER
Penicillin-Streptomycin	Invitrogen	Cat#15140122
PhosSTOP	Roche	Cat#04906845001
Poly-L-lysine	Sigma-Aldrich	Cat#P4707
Poly-L-ornithine	Sigma-Aldrich	Cat#P3655
Protease inhibitor cocktail	Roche	Cat#04693132001
Puromycin	Nacalai Tesque	Cat#14861-71
Quercetin	Sigma-Aldrich	Cat#S2391
Rapamycin	Selleck Chemicals	Cat#S1039
RIPA Lysis and Extraction Buffer	Thermo Fisher Scientific	Cat#89900
RNeasy Plus Mini Kit	QIAGEN	Cat#74134
SB431542	Nacalai Tesque	Cat#18176-54
StemFit AK02N	Takara Bio	Cat#AK02N
STEM-CELLBANKER	Takara Bio	Cat#11924/CB045
SYBR Premix Ex Taq II	Takara Bio	Cat#RR820
Transforming growth factor β3 (TGF-β3)	BioLegend	Cat#585802
TrypLE Select	Thermo Fisher Scientific	Cat#12563-029
VE821	Sigma-Aldrich	Cat#SML1415
XAV939	Selleck Chemicals	Cat#S1180
Y-27632	FUJIFILM Wako	Cat#257-00614
**Critical commercial assays**		
7-Aminoactinomycin D (7-AAD)	Nacalai Tesque	Cat#19175-34
Annexin V-FITC Apoptosis Detection Kit	Nacalai Tesque	Cat#15342-54
Caspase-Glo 3/7 Assay System	Promega	Cat#G8090
CellTiter-Blue Cell Viability Assay	Promega	Cat#G8080
CellTiter-Glo 2.0 Cell Viability Assay	Promega	Cat#G9241
Human β-amyloid (1–40) ELISA kit	FUJIFILM Wako	Cat#292-62301
Human β-amyloid (1–42) ELISA kit	FUJIFILM Wako	Cat#294-62501
Human Cytokine Array C1000	RayBiotech	Cat#AAH-CYT-1000-8
NAD/NADH-Glo Assay	Promega	Cat#G9071
SPiDER-βGal	Dojindo Molecular Technologies	Cat#SG03
**Deposited data**		
RNA-seq data from this study	This study	DRA: PRJDB17588
**Experimental models: Cell lines**		
Human iPSC line: 201B7: control; F, 36 years; dermal fibroblasts; retrovirus; on-feeder	(Takahashi et al., 2007)	201B7

(*Continued on next page*)

Continued

REAGENT or RESOURCE	SOURCE	IDENTIFIER
Human iPSC line: WD39: control; F, 16 years; dermal fibroblasts; retrovirus; feeder-free	(Imaizumi et al., 2012)	WD39
Human iPSC line: C1: control; F, 24 years; PBMC; Sendai virus; on-feeder	(Hoashi et al., 2017)	C1
Human iPSC line: C2: control; M, 30 years; PBMC; Sendai virus; on-feeder	(Hoashi et al., 2017)	C2
Human iPSC line: TKA4: control; M, 40 years; PBMC; Sendai virus; on-feeder	(Matsumoto et al., 2016)	TKA4
Human iPSC line: JA5: control; M, 47 years; PBMC; Sendai virus; feeder-free	(Ishikawa et al., 2024b)	JUCGRMi005-A (JA5)
Human iPSC line: JB6: control; F, 55 years; PBMC; Sendai virus; feeder-free	(Ishikawa et al., 2024c)	JUCGRMi006-A (JB6)
Human iPSC line: JC9: control; M, 70 years; PBMC; Sendai virus; feeder-free	This paper	JC9
Human iPSC line: PS1: PSEN1 c.737C>A (p.Ala246Glu); F, 31 years; PBMC; Sendai virus; feeder-free	Coriell Institute	AG25367
Human iPSC line: PH7: PARK2 exon 3 homozygous deletion; F, 25 years; PBMC; Sendai virus; feeder-free	(Ishikawa et al., 2024a)	JUCGRMi003-A (PH7)
Human iPSC line: PKB4: PARK6 c.1162T>C (p.Cys388Arg); F, 61 years; dermal fibroblasts; retrovirus; feeder-free	(Shiba-Fukushima et al., 2017)	PKB4
Human iPSC line: CHA11: PARK22 c.182C>T (p.Thr61Ile); M, 45 years; dermal fibroblasts; episomal vector; feeder-free	(Ikeda et al., 2019)	CHA11
Human dermal fibroblasts: young donor; F, 17 years	(Imaizumi et al., 2012)	WD (young fibroblasts)
Human dermal fibroblasts: aged donor; M, 66 years	This paper	Aged fibroblasts
Human neuroblastoma cell line: SH-SY5Y	ATCC	Cat#CRL-2266
Human hiPSC-derived neurons: ReproNeuro AD-mutation; PSEN1 p.P117L	REPROCELL	Cat#RCDN002N
Human hiPSC-derived neurons: ReproNeuro wild-type control	REPROCELL	Cat#RCDN001N
**Oligonucleotides**		
Primer: NAMPT Forward: TGTTCCAGCAGCAGAACACA	This paper	N/A
Primer: NAMPT Reverse: GCTGACCACAGATACAGGCA	This paper	N/A
Primer: NMNAT Forward: GCTGGCCAAGGACTACATGA	This paper	N/A
Primer: NMNAT Reverse: AGTTCTGCCATGATGACCCG	This paper	N/A

(*Continued on next page*)

Continued

REAGENT or RESOURCE	SOURCE	IDENTIFIER
Primer: LMNB1 Forward: CCTTCTTCCCGTGTGACAGT	This paper	N/A
Primer: LMNB1 Reverse: AGGCGGAATGAGAGATGCTA	This paper	N/A
Primer: LMNB2 Forward: CATCTCCGTCATCTCCTGCT	This paper	N/A
Primer: LMNB2 Reverse: TGGAGTCCCTCAGCTACCAG	This paper	N/A
Primer: ACTB (β-actin) Forward: TGAAGTGTGACGTGGACATC	This paper	N/A
Primer: ACTB (β-actin) Reverse: GGAGGAGCAATGATCTTGAT	This paper	N/A
**Software and algorithms**		
IN Cell Developer Toolbox v1.9	GE Healthcare	N/A
CellPathfinder	Yokogawa	N/A
ImageJ	NIH	https://imagej.nih.gov/
pCLAMP 10.7	Molecular Devices	N/A
FastQC	Babraham Bioinformatics	https://www.bioinformatics.babraham.ac.uk/projects/fastqc/
Trim Galore v0.6.6	Babraham Bioinformatics	https://www.bioinformatics.babraham.ac.uk/projects/trim_galore/
HISAT2 v2.2.1	(Kim et al., 2015)	https://daehwankimlab.github.io/hisat2/
SAMtools v1.11	(Li et al., 2009)	https://www.htslib.org/
featureCounts v1.6.4	(Liao et al., 2014)	https://subread.sourceforge.net/featureCounts.html
edgeR	Bioconductor	https://www.bioconductor.org/packages/edgeR
clusterProfiler	(Wu et al., 2021)	https://bioconductor.org/packages/release/bioc/html/clusterProfiler.html
TCC	(Sun et al., 2013)	N/A
baySeq	(Osabe et al., 2019)	N/A
plotly	Plotly	https://plotly.com/
FlowJo v10.8.1	BD Biosciences	N/A
guavaSoft 3.3	Millipore	N/A
R v4.4.2	R Foundation for Statistical Computing	https://www.r-project.org/

EXPERIMENTAL MODEL AND STUDY PARTICIPANT DETAILS

Culture of hiPSCs

The hiPSC lines used in this study are listed in the key resources table. Their basic characterization, including authentication, pluripotency, genomic characterization, and genetic mutations, has been described in previous reports, and the cells were cryopreserved using STEM-CELLBANKER. All hiPSCs were used at passages 20–50 and were routinely tested

for mycoplasma contamination and sterility. For on-feeder culture, hiPSCs were maintained on mitomycin C-treated SNL murine fibroblast feeder cells in DMEM/F12 supplemented with 20% KnockOut Serum Replacement, 2 mM L-glutamine, 0.1 mM nonessential amino acids, 0.1 mM 2-mercaptoethanol, 0.5% penicillin/streptomycin, and 4 ng/mL fibroblast growth factor 2 (FGF2) under a 3% CO_2 atmosphere. Cells were passaged every 7 days using a dissociation solution at a 1:5 split ratio. For feeder-free culture, hiPSCs were maintained on iMatrix-511-coated plates in StemFit AK02N medium under a 5% CO_2 atmosphere and passaged every 7 days using TrypLE Select at a split density of 1.5×10^3 cells/cm^2. All experimental procedures involving hiPSCs were approved by the Juntendo University School of Medicine Ethics Committee (M08-0449) and the Keio University School of Medicine Ethics Committee (20080016).

Culture of human dermal fibroblasts

The human dermal fibroblasts used in this study are listed in the key resources table. Cells were maintained in high-glucose DMEM supplemented with 10% fetal bovine serum (FBS) and 100 U/mL penicillin-streptomycin at 37°C under a 5% CO_2 atmosphere. Cells at passages 4–7 were used for all experiments. Unless otherwise specified, cells were treated with 10 μM KU60019. All experimental procedures involving fibroblasts were approved by the Keio University School of Medicine Ethics Committee (20-16-18).

Culture of SH-SY5Y cells

SH-SY5Y cells were maintained in high-glucose DMEM supplemented with 10% FBS and 100 U/mL penicillin-streptomycin at 37°C under a 5% CO_2 atmosphere. Unless otherwise specified, cells were treated with 10 μM KU60019 for 72 h prior to analysis.

Human hiPSC-derived neurons for PSEN1 assays

For cell viability and Aβ ELISA assays, hiPSC-derived neurons carrying the PSEN1 p.P117L mutation (ReproNeuro AD-mutation, RCDN002N) and wild-type control neurons (ReproNeuro, RCDN001N) were used according to the manufacturer's instructions. Cells were cultured in ReproNeuro culture medium for 14–30 days in the presence or absence of 5 μM KU60019 prior to analysis. Sex information for the commercially obtained ReproNeuro cells was not available from the provider.

METHOD DETAILS

Differentiation of hiPSCs into dopaminergic neurons

Dopaminergic neuron progenitor cells were generated from hiPSCs using two previously established methods: neurosphere-based differentiation (Nakamura et al., 2023) and floor plate-based differentiation from feeder-free hiPSCs (Gantner et al., 2020). Progenitor cells obtained by both methods were plated onto poly-L-ornithine- and fibronectin-coated plates for terminal differentiation. Cells were cultured in KBM neural stem cell medium supplemented with B27 Plus, CultureOne, 20 ng/mL brain-derived neurotrophic factor (BDNF), 20 ng/mL glial cell line-derived neurotrophic factor (GDNF), 0.2 mM ascorbic acid, 0.5 mM dibutyryl cyclic AMP (dbcAMP), 1 ng/mL transforming growth factor β3 (TGF-β3), 10 μM DAPT, 3 μM CHIR99021, and 10 μM Y-27632. Half of the medium was replaced every 3–4 days with fresh medium lacking CHIR99021 and Y-27632. KU60019 (5 μM) was added at the time of seeding of neural progenitor cells and maintained throughout the differentiation period, typically 14 days, until analysis.

Compound screening

For compound screening, 201B7 hiPSCs were dissociated and seeded at a density of 10 cells/μL in KBM neural stem cell medium supplemented with B27, 20 ng/mL FGF2, 10 ng/mL hLIF, and 10 μM Y-27632 under a 3% CO_2 atmosphere to form floating neurospheres. Neurospheres were dissociated using TrypLE Select and passaged up to six times. Dissociated neurospheres were then plated onto poly-L-ornithine- and fibronectin-coated plates and cultured in KBM neural stem cell medium supplemented with B27, 20 ng/mL BDNF, 20 ng/mL GDNF, 200 μM ascorbic acid, 0.5 mM dbcAMP, and 1 ng/mL TGF-β3. Cells were infected with a lentiviral vector encoding a synapsin promoter-driven GFP reporter. After 24 h, the medium was replaced with an inhibitor library at a final concentration of 10 μM. The medium was subsequently replaced every 2–3 days. After 17 days of differentiation, neuronal maturation was quantified based on the mean GFP fluorescence intensity in each well using an IN Cell Analyzer 1000.

Senolytic drug treatment

Young fibroblasts were cultured with 10 μM KU60019 for 2 days. The medium was then replaced with fresh medium containing 10 μM KU60019 and 10 μM senolytic drugs, followed by incubation for 24 h. The following senolytic drugs were used: rapamycin, metformin, ABT-737, 17DMAG, azithromycin, fisetin, dasatinib, and quercetin. For hiPSC-derived neurons, 2.5 μM 17DMAG was added during the final 3 days of a 14-day differentiation period in the presence of 5 μM KU60019.

Immunostaining and quantification

Cells were fixed in 4% paraformaldehyde for 30 min and blocked with 5% fetal bovine serum and 0.1% Triton X-100 for 30 min at room temperature (23°C–27°C). Cells were then incubated with primary antibodies diluted in blocking solution at 4°C for 12–18 h. After washing with phosphate-buffered saline, cells were incubated with appropriate secondary antibodies and Hoechst 33258 (0.5 μg/mL) for 1 h at room temperature. Images were acquired using BZ-X800, IN Cell Analyzer 2200, or a CQ1 confocal image cytometer.

For quantification, images were randomly acquired from 16 fields per well using the IN Cell Analyzer 2200 and analyzed with IN Cell Developer Toolbox v1.9. When co-stained with MAP2, analyses were restricted to MAP2-positive cells. For assessment of Lamin B1-associated nuclear abnormalities, cells with abnormal nuclear morphology were defined as those exhibiting a luminance value <1500 and a perinuclear length of 0.5–0.8, as previously described (Freund et al., 2012). For DNA damage analysis, cells containing three or more nuclear γH2AX or 53BP1 foci were classified as positive (Zorin et al., 2019). For analysis of CCF and SA-βGal-positive cell populations, as well as p-tau and total tau detection, images were acquired using a CQ1 confocal image cytometer and analyzed with CellPathfinder. The pTau/Tau ratio was calculated as the ratio of integrated fluorescence intensities (area × mean intensity) and averaged per well.

SA-βGal staining and quantification

SA-βGal staining was performed using the Cellular Senescence Detection Kit - SPiDER-βGal according to the manufacturer's instructions. Prior to staining, the medium was replaced with medium supplemented with 1 μM bafilomycin A1, and cells were incubated at 37°C for 1 h. Cells were then stained with 20 μM SPiDER-βGal solution at 37°C for 1 h and fixed in 4% paraformaldehyde for 30 min. Following SA-βGal staining, immunostaining was performed as described above. Neurons were identified by MAP2 staining where applicable. Images were acquired using BZ-X800, IN Cell Analyzer 2200, or a CQ1 confocal image cytometer, and quantified using the same criteria as described above. For flow cytometric analysis, SA-βGal-positive cells were quantified using a Guava easyCyte System.

Sparse labeling and neurite length measurement

hiPSC-derived secondary neurospheres containing dopaminergic neuron progenitors were dissociated using Accutase and cryopreserved in STEM-CELLBANKER. Cells were thawed, infected with AAV1-CAG-tdTomato for 15 min, plated on poly-L-lysine-coated 35-mm plastic dishes at a density of 2 × 10 ^4 cells/dish, and co-cultured with unlabeled cells at a density of 48 × 10 ^4 cells/dish. The culture medium was replaced with KBM neural stem cell medium supplemented with 2% B27, 20 ng/mL BDNF, 20 ng/mL GDNF, 0.2 mM ascorbic acid, 0.5 mM dbcAMP, 1 ng/mL TGF-β3, and 10 μM DAPT, with or without 5 μM KU60019. tdTomato-labeled cells were imaged at 3, 5, and 7 days after plating using an iX73 microscope with ×4 and ×10 dry objectives. The length of the longest neurite per cell was measured using ImageJ.

Whole-cell patch-clamp recordings

Coverslips containing hiPSC-derived neurons treated with or without KU60019 were transferred to a recording chamber mounted on the stage of an Olympus BX51WI upright microscope equipped with an infrared differential interference contrast imaging system. For KU60019-treated samples, treatment was maintained until the time of recording. Recordings were performed in a standard extracellular solution maintained at 23°C–27°C, containing 130 mM NaCl, 26 mM NaHCO3, 10 mM glucose, 3 mM KCl, 2 mM CaCl2, 2 mM MgCl2, and 1.25 mM NaH2PO4. The solution was continuously perfused at 1–2 mL/min and oxygenated with 95% O2 and 5% CO2 to maintain pH 7.4. Neurons with a compact soma and at least two extended processes were selected for recording. Patch pipettes were fabricated from borosilicate glass capillaries (GD-1.5; Narishige) using a P-97 puller (Sutter Instrument). The internal solution (pH 7.25, 285–300 mOsm) contained 10 mM KCl, 130 mM K-gluconate, 10 mM HEPES, 0.4 mM EGTA, 2 mM MgCl2, 0.3 mM Na2-GTP, and 2 mM Mg-ATP. Pipette resistance ranged from 2.5 to 5.0 MΩ. Electrophysiological signals were recorded using a MultiClamp 700B amplifier, filtered at 10 kHz, and digitized at 20 kHz using a Digidata 1440A. Access resistance was continuously monitored throughout the recordings. Data were analyzed using pCLAMP 10.7. Voltage responses

were recorded in current-clamp mode by applying 300 ms current steps from −60 pA to +150 pA in 10 pA increments at a holding potential of −60 mV, delivered at 0.5 Hz.

Western blotting

Cells were lysed in RIPA buffer supplemented with a protease inhibitor cocktail and PhosSTOP, followed by centrifugation at 15,300 × g for 15 min at 4°C. The supernatant was mixed with NuPAGE LDS sample buffer, and proteins were separated by SDS-PAGE. Proteins were transferred onto PVDF membranes, which were then blocked with PVDF Blocking Reagent for Can Get Signal and incubated with primary antibodies at 4°C for 12–18 h. After washing, membranes were incubated with appropriate secondary antibodies for 1 h, and immunoreactive signals were detected by chemiluminescence using ECL Prime reagent and a Fusion Solo 7S imaging system.

Measurement of total NAD/NADH

Intracellular NAD/NADH levels were measured using a NAD/NADH-Glo Assay. Neurons were cultured in 96-well plates for 14 days, and an equal volume of the reaction solution was added to each well according to the manufacturer's instructions with gentle agitation. Luminescence was measured using a Mithras LB943 multimode plate reader.

Cytokine array

Young fibroblasts were cultured in serum-free medium with or without KU60019 for 3 days. Cytokines in the conditioned medium were detected using a Human Cytokine Array C1000 according to the manufacturer's instructions. Membranes were first incubated with blocking buffer for 30 min at room temperature, followed by incubation with samples at 4°C for 12–18 h. After washing with the provided wash buffer, membranes were incubated with a biotinylated antibody cocktail at 4°C for 12–18 h. Membranes were then washed and incubated with HRP-conjugated streptavidin for 2 h at room temperature. After additional washing, signals were developed using the detection buffer. Images were acquired using a Fusion Solo 7S imaging system and quantified using ImageJ. Signal intensities were normalized to total cellular protein content.

Quantitative reverse transcription PCR and RNA-seq analysis

Total RNA was extracted from cells using the RNeasy Plus Mini Kit and reverse-transcribed to generate cDNA. Quantitative RT-PCR was performed using a QuantStudio 6 Real-Time PCR System with SYBR Premix Ex Taq II and gene-specific primers. Primer sequences are provided in the key resources table.

For RNA-seq analysis, libraries were prepared using the NEBNext Ultra RNA Library Prep Kit for Illumina with poly(A) selection according to the manufacturer's instructions. Libraries were sequenced on a NovaSeq 6000 with a read depth of >20 million reads per sample. The RNA-seq data have been deposited in the DDBJ Sequence Read Archive (accession number: PRJDB17588). Raw sequence data were quality-checked using FastQC and processed with TrimGalore v0.6.6. Reads were mapped to the human reference genome (GRCh38) using HISAT2 v2.2.1 (Kim et al., 2015). BAM files were generated using SAMtools v1.11 (Li et al., 2009), and gene-level read counts were obtained using featureCounts v1.6.4 (Liao et al., 2014). Differential expression analysis was performed using the edgeR package based on a generalized linear model framework (Sun et al., 2013). Genes with $|\log 2 \text{ fold change}| \geq 1$ and a false discovery rate <0.05 were defined as differentially expressed genes. Functional enrichment analysis was performed using the enrichKEGG function in the clusterProfiler package (Wu et al., 2021). For multi-group comparisons, normalized count data obtained using TCC normalization were analyzed using the baySeq algorithm with an empirical Bayesian approach (Osabe et al., 2019). Principal component analysis was performed on log10(x + 1)-transformed data and visualized in 3D using the plotly package.

PSEN1-variant hiPSC-derived neurons

For p-tau/total tau analysis, hiPSCs were differentiated into cortical excitatory neurons based on a previously reported protocol combining NGN2 programming with developmental patterning (Kondo et al., 2017), with minor modifications. To induce neuronal differentiation, hiPSCs carrying a piggyBac-integrated, doxycycline-inducible NGN2 cassette were seeded onto iMatrix-511-coated plates in StemFit AK02N medium supplemented with Y-27632. On day 1, the medium was replaced with KBM neural stem cell medium supplemented with B27 Plus, doxycycline, SB431542 (10 μM), LDN-193189 (100 nM), and XAV939 (2 μM) for 2 days. On day 3, inhibitors were removed and puromycin/G418 selection was applied. On day 4, cells were dissociated using Accutase and replated onto poly-L-ornithine/laminin-coated plates, defined as DIV0. Neurons were maintained in maturation medium consisting of KBM neural stem cell medium supplemented with B27 Plus, 200 μM ascorbic acid, 500 μM dbcAMP, 20 ng/mL BDNF, and 20 ng/mL GDNF, with half-volume

medium changes twice per week. KU60019 (5 μM) was added to the maturation medium from DIV0 to DIV14. Cells were subsequently maintained without KU60019 and analyzed at DIV28 or DIV42.

Measurement of Aβ40 and Aβ42 by ELISA

For Aβ ELISA assays, neurons were seeded in 96-well plates at a density of 1 × 10 ˆ5 cells/well. The following day, the medium was replaced with ReproNeuro culture medium supplemented with 5 μM KU60019. Culture supernatants were collected on days 21 and 30 to measure Aβ40 and Aβ42 levels using human β-amyloid (1–40) and human β-amyloid (1–42) ELISA kits, respectively. The Aβ42/40 ratio was calculated using culture supernatants collected from the same well.

Cell viability and caspase-3/7 assays

The viability of neurons or fibroblasts cultured in 96-well plates was measured using the CellTiter-Glo 2.0 Cell Viability Assay according to the manufacturer's instructions. Luminescence was measured using a Mithras LB943 multimode plate reader. For caspase-3/7 activity measurements, the viability of hiPSC-derived neurons was first assessed in the same wells using CellTiter-Blue reagent, and fluorescence intensity was used as an internal control. Caspase-3/7 activity was then measured using the Caspase-Glo 3/7 Assay System following incubation for 1 h. Luminescence was measured using the same plate reader, and caspase-3/7 activity was normalized to the corresponding CellTiter-Blue fluorescence intensity.

Annexin V/7-AAD apoptosis assay

Neurons were dissociated using Accutase and stained with an Annexin V-FITC Apoptosis Detection Kit and 7-aminoactinomycin D according to the manufacturer's instructions. Data were acquired using a Guava easyCyte System and analyzed using FlowJo v10.8.1, R v4.4.2, and guavaSoft 3.3. Apoptotic cells were identified based on Annexin V and 7-AAD staining.

QUANTIFICATION AND STATISTICAL ANALYSIS

Statistical analysis

Data are presented as mean ± standard error of the mean (SEM). Statistical significance was defined as $p \leq 0.05$. Statistical analyses were performed using R v4.4.2. Exact statistical tests, sample sizes, and the definition of n for each experiment are provided in the figure legends.

Stem Cell Reports, Volume *21*

Supplemental Information

Suppression of ATM kinase signaling accelerates cellular senescence

Kei-ichi Ishikawa, Takahiro Shiga, Takumi Hirose, Naoko Kuzumaki, Sakura Miyoshi, Akihiro Yamaguchi, Hidetaka Tamune, Avijite Kumer Sarkar, Kento Nakai, Kazuyoshi Baba, Shigeo Okabe, Nobutaka Hattori, Hideyuki Okano, and Wado Akamatsu

Figure S1.

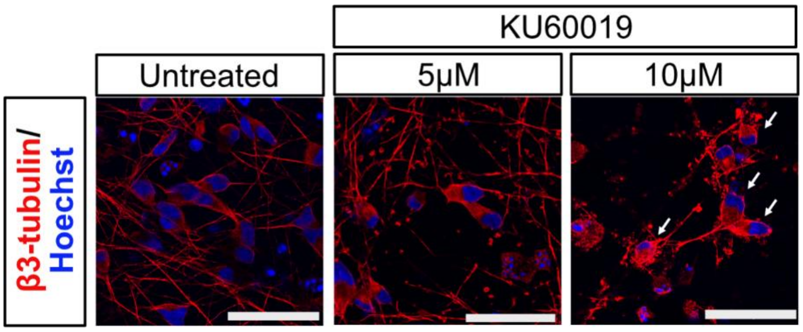

Figure S1. High-dose KU60019 treatment induces neurotoxic morphological changes, related to Figure 1.

Representative images of β3-tubulin immunostaining in 201B7 hiPSC-derived dopaminergic neurons treated with or without KU60019. Neurons treated with 10 μM KU60019 showed axonal fragmentation at day 10 (arrows). Scale bar, 50 μm.

Figure S2.

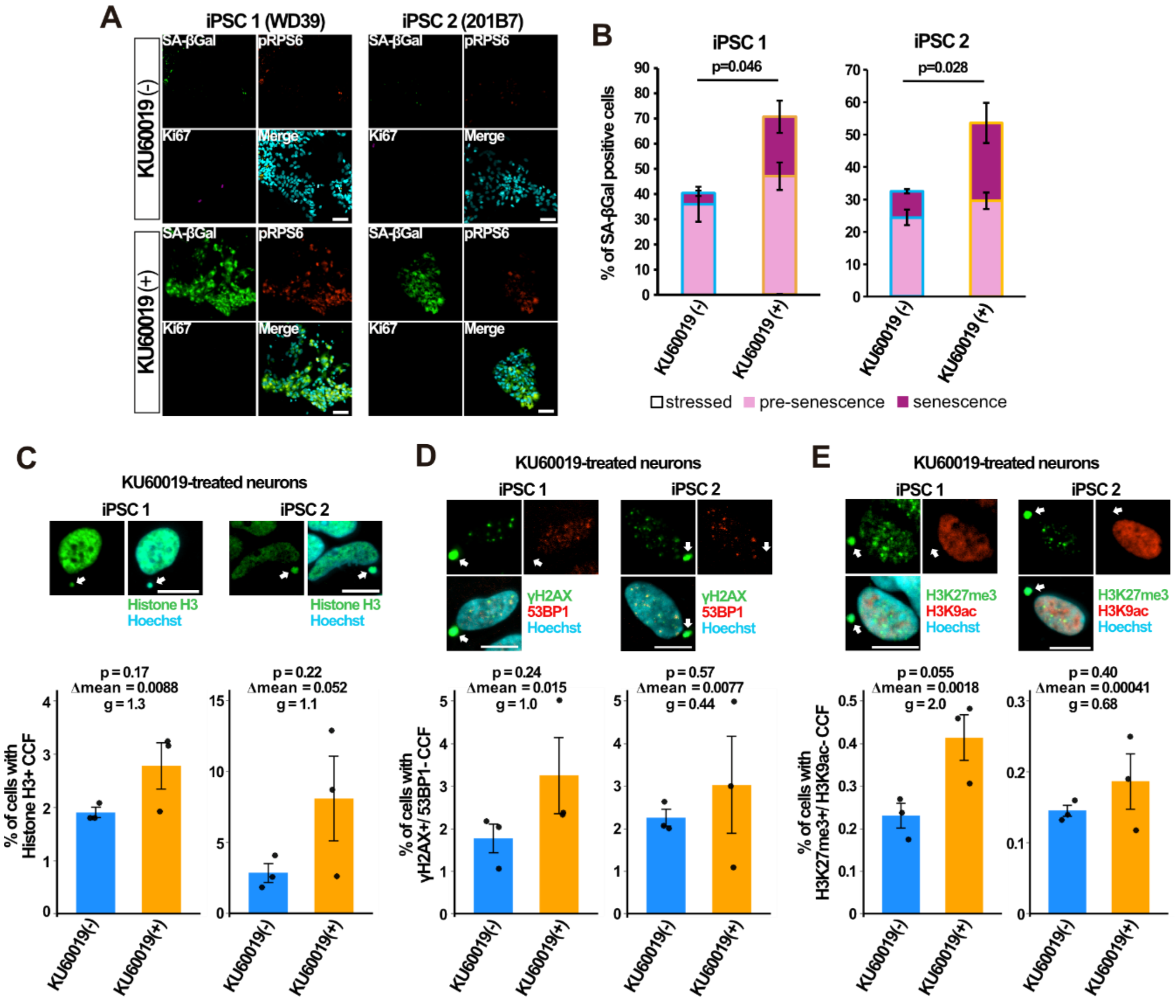

Figure S2. KU60019-treated hiPSC-derived neurons exhibit senescence-associated phenotypes, related to Figure 2.

(A) Representative images of SA-βGal, pRPS6, and Ki67 staining in untreated and KU60019-treated neurons after 14 days of differentiation. Scale bar, 100 μm.

(B) Percentage of senescent (SA-βGal^{+}, pRPS6^{+}, Ki67^{-}), pre-senescent (SA-βGal^{+}, pRPS6^{-}), and stressed (SA-βGal^{+}, pRPS6^{+}, Ki67^{+}) cells. n = 4 independent experiments.

(C) Representative images of Histone H3 and Hoechst 33258 staining in KU60019-treated neurons after 14 days of differentiation (upper panels). Scale bar, 10 μm. Lower panels show the percentage of cells with histone H3^{+} cytoplasmic chromatin fragments (CCFs). CCFs were defined as Hoechst^{+} extranuclear DNA structures. n = 3 independent experiments.

(D) Representative images of γH2AX, 53BP1, and Hoechst 33258 staining in KU60019-treated neurons (upper panels). Scale bar, 10 μm. Lower panels show the percentage of cells with γH2AX^{+}, 53BP1^{-} CCFs. n = 3 independent experiments.

(E) Representative images of H3K27me3, H3K9ac, and Hoechst 33258 staining in KU60019-treated neurons (upper panels). Scale bar, 10 μm. Lower panels show the percentage of cells with H3K27me3^{+}, H3K9ac^{-} CCFs. n = 3 independent experiments.

Data are shown as mean ± SEM. *P* values, mean differences (Δmean), and effect sizes (Hedges' g) are indicated. *P* values were calculated using Welch's t-test.

Figure S3.

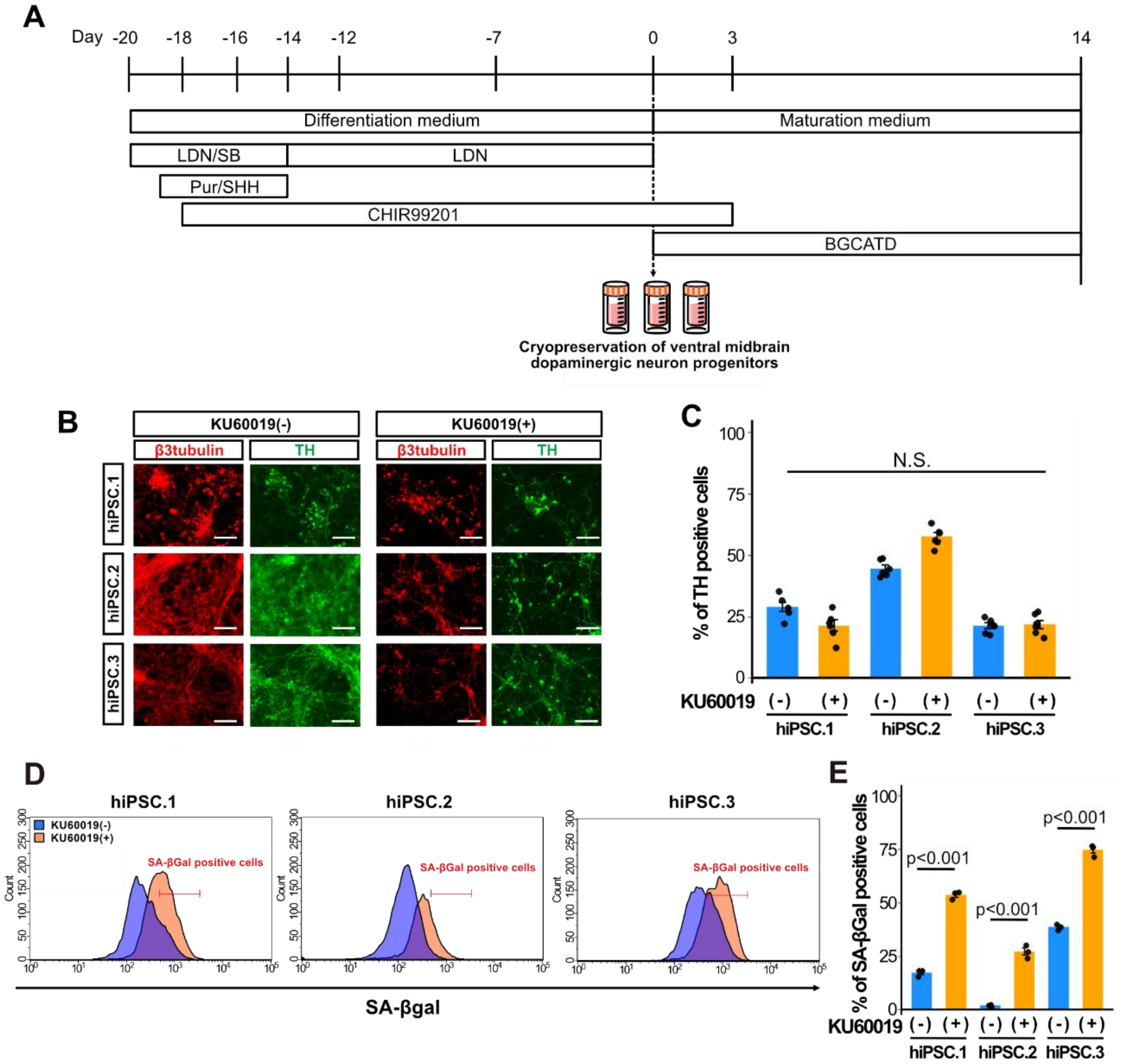

Figure S3. KU60019 induces senescence-associated phenotypes in dopaminergic neurons generated using the floor plate method, related to Figure 2.

(A) Schematic overview of the differentiation schedule for the floor plate method. LDN, LDN-193189; SB, SB431542; Pur, purmorphamine; SHH, sonic hedgehog; B, BDNF; G, GDNF; C, dibutyryl cAMP; A, ascorbic acid; T, TGF-β3; D, DAPT.

(B) Representative images of β3-tubulin and TH immunostaining in untreated and KU60019-treated hiPSC-derived dopaminergic neurons after 14 days of differentiation. Scale bar, 100 µm.

(C) Percentage of TH^+ dopaminergic neurons. n = 6 independent experiments.

(D) Representative flow cytometry histograms of SA-βGal^+ cells in untreated and KU60019-treated dopaminergic neurons after 14 days of differentiation.

(E) Percentage of SA-βGal^+ cells. n = 3 independent experiments.

Data are shown as mean ± SEM. *P* values were calculated using two-way ANOVA (condition × cell line), followed by Holm-adjusted post hoc comparisons between untreated and KU60019-treated groups within each cell line where applicable. N.S., not significant.

Figure S4.

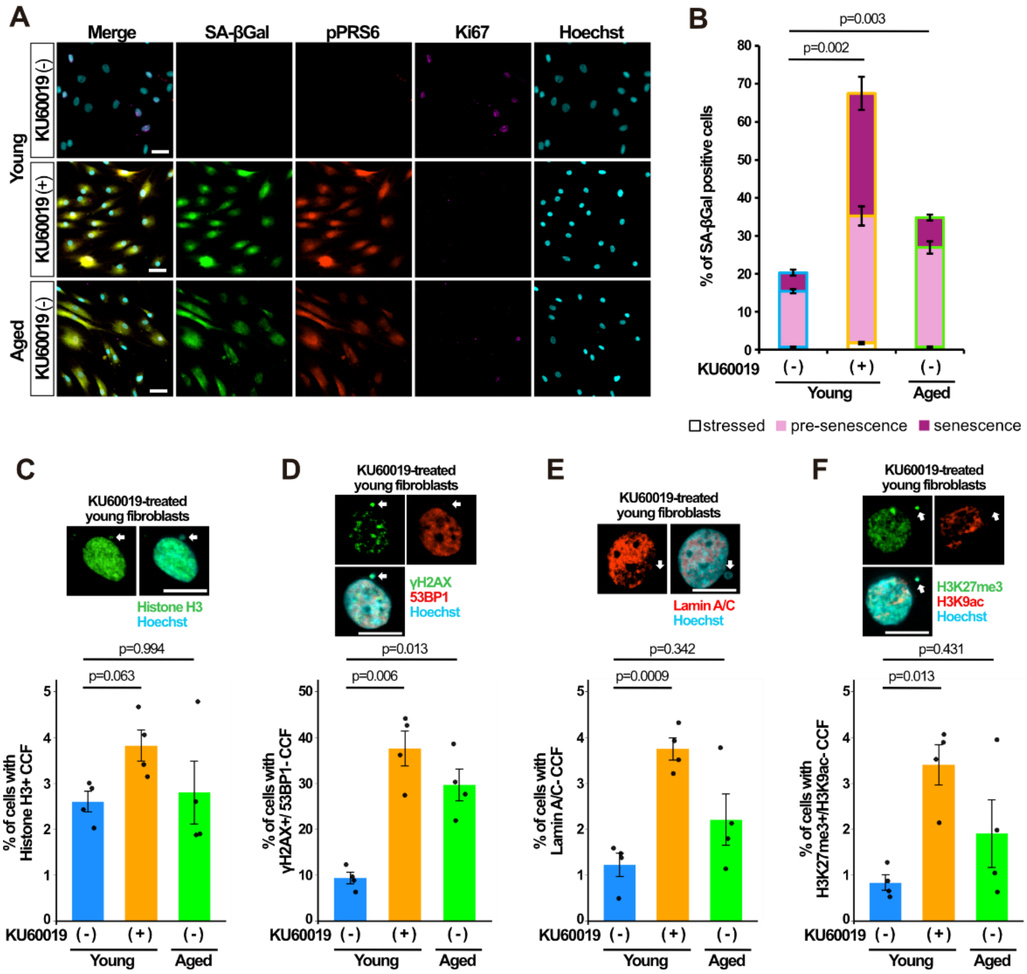

Figure S4. KU60019-treated fibroblasts exhibit senescence-associated phenotypes, related to Figure 3.

(A) Representative images of SA-βGal staining and immunostaining for pRPS6 and Ki67 in untreated young, KU60019-treated young, and aged fibroblasts. Scale bar, 100 μm.

(B) Percentage of senescent (SA-βGal$^+$, pRPS6$^+$, Ki67$^-$), pre-senescent (SA-βGal$^+$, pRPS6$^-$), and stressed (SA-βGal$^+$, pRPS6$^+$, Ki67$^+$) cells. n = 4 independent experiments.

(C) Representative images of histone H3 and Hoechst 33258 staining in KU60019-treated young fibroblasts (upper panels). Scale bar, 10 μm. Lower panel shows the percentage of cells with histone H3$^+$ cytoplasmic chromatin fragments (CCFs). CCFs were defined as Hoechst$^+$ extranuclear DNA structures. n = 3 independent experiments.

(D) Representative images of γH2AX, 53BP1, and Hoechst 33258 staining in KU60019-treated young fibroblasts (upper panels). Scale bar, 10 μm. Lower panel shows the percentage of cells with γH2AX$^+$, 53BP1$^-$ CCFs. n = 3 independent experiments.

(E) Representative images of lamin A/C and Hoechst 33258 staining in KU60019-treated young fibroblasts (upper panels). Scale bar, 10 μm. Lower panel shows the percentage of cells with lamin A/C^{-} CCFs. n = 3 independent experiments.
(F) Representative images of H3K27me3, H3K9ac, and Hoechst 33258 staining in KU60019-treated young fibroblasts (upper panels). Scale bar, 10 μm. Lower panel shows the percentage of cells with H3K27me3^{+}, H3K9ac^{-} CCFs. n = 3 independent experiments.
Data are shown as mean ± SEM. *P* values were calculated using Welch's ANOVA followed by Holm-adjusted Games–Howell post hoc comparisons.

Figure S5.

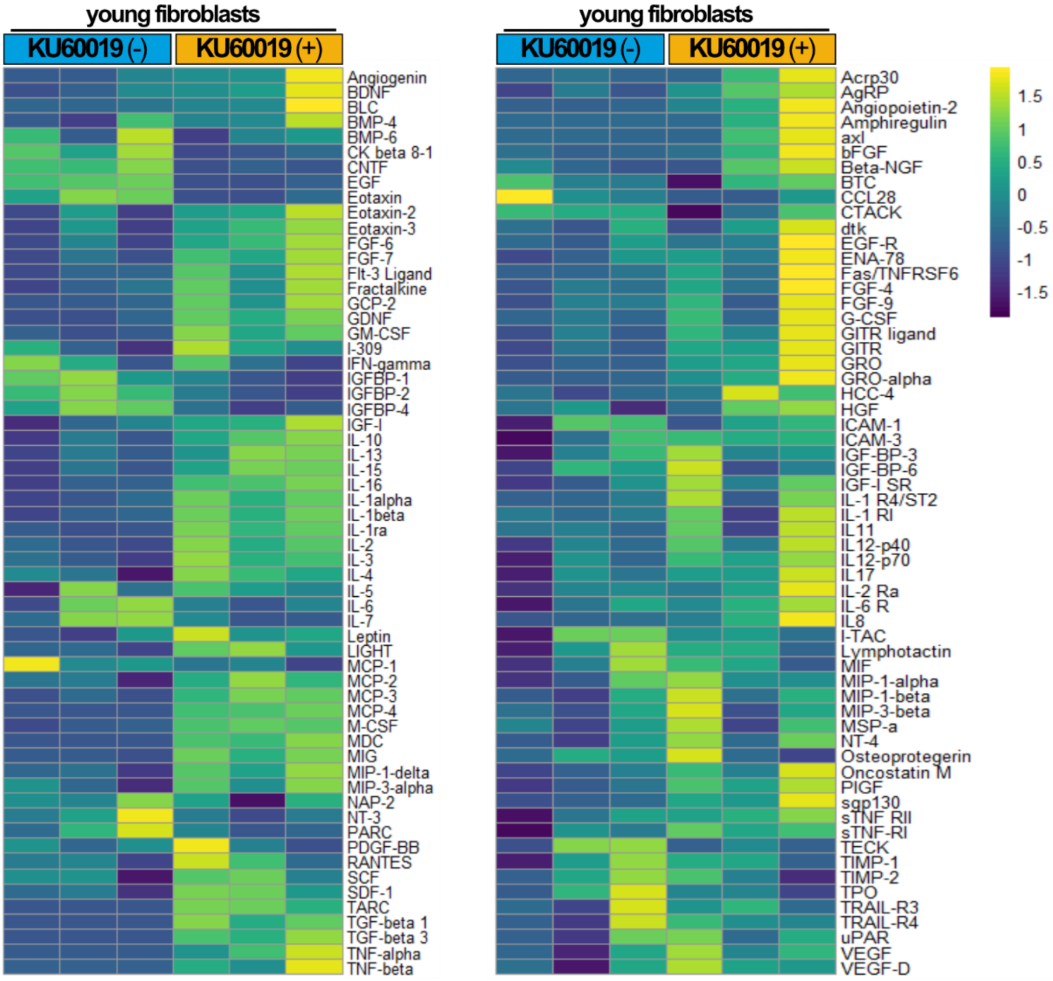

Figure S5. KU60019 treatment induces SASP factor secretion in young fibroblasts, related to Figure 3.

Heatmap of senescence-associated secretory phenotype (SASP) factors detected using a cytokine array in conditioned media from untreated and KU60019-treated young fibroblasts. Each column represents an independent experiment. n = 3 independent experiments per condition. Values represent normalized signal intensities for each factor relative to the untreated condition.

Figure S6.

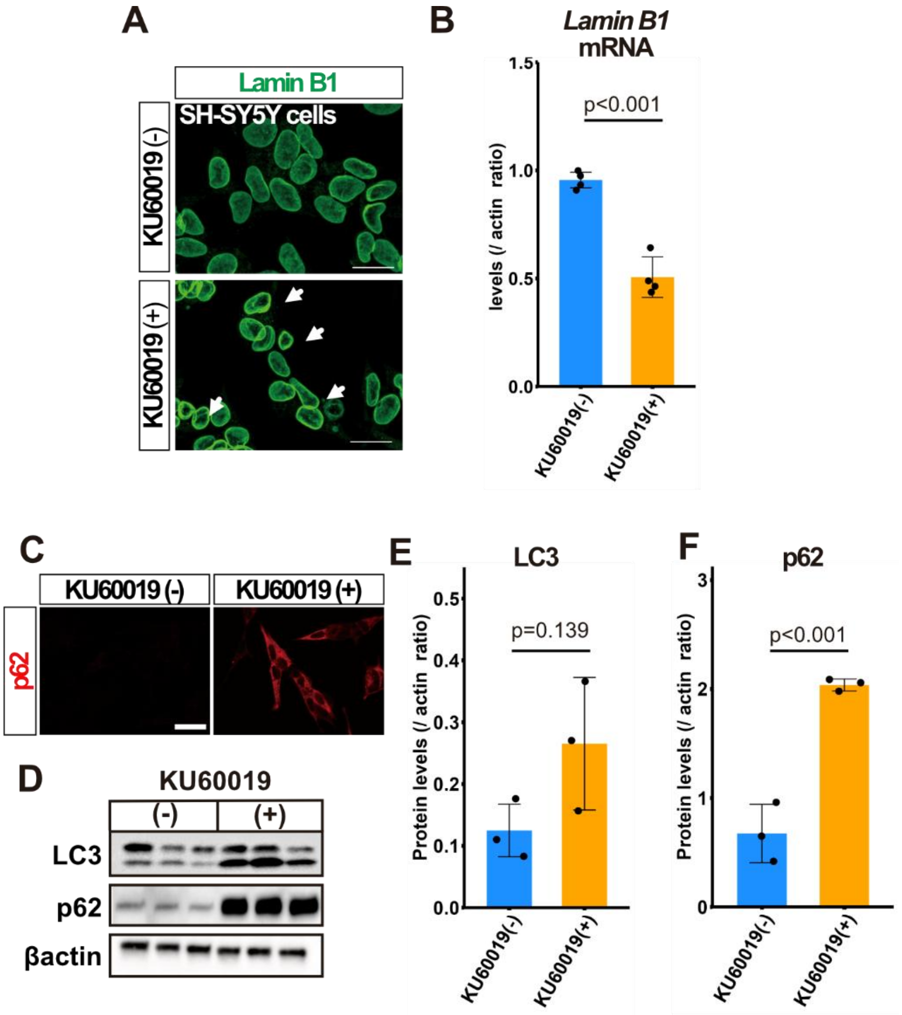

Figure S6. KU60019-treated SH-SY5Y cells exhibit senescence-associated phenotypes, related to Figure 3.

(**A**) Representative images of lamin B1 immunostaining in untreated and KU60019-treated SH-SY5Y cells. Scale bar, 10 μm.

(**B**) Lamin B1 mRNA expression levels. n = 4 independent experiments.

(**C**) Representative images of p62 immunostaining in untreated and KU60019-treated SH-SY5Y cells. Scale bar, 10 μm.

(**D**) Immunoblot analysis of LC3 and p62 protein levels in untreated and KU60019-treated SH-SY5Y cells.

(**E, F**) Quantification of the immunoblot results shown in (**D**). n = 3 independent experiments. Data are shown as mean ± SEM. *P* values were calculated using Welch's t-test.

Figure S7.

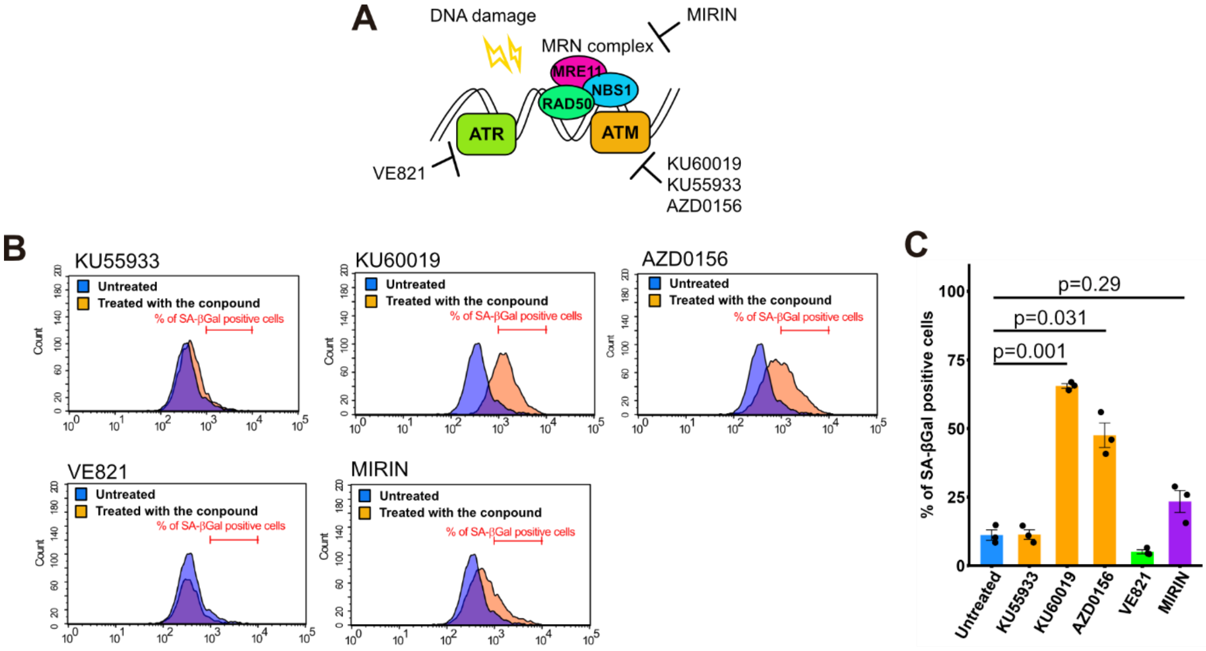

Figure S7. Screening of DNA repair inhibitors for their ability to accelerate senescence-associated phenotypes, related to Figure 3.

(A) Schematic illustration of inhibitors targeting factors involved in ATM-mediated DNA repair.

(B) Histograms showing the distribution of SA-βGal staining intensity in young fibroblasts treated with the indicated inhibitors. Blue indicates untreated cells, and orange indicates inhibitor-treated cells.

(C) Percentage of SA-βGal^{+} cells. Blue bars represent untreated cells, orange bars represent ATM inhibitor–treated cells, green bars represent ATR inhibitor–treated cells, and purple bars represent MRN complex inhibitor–treated cells. n = 3 independent experiments.

Data are shown as mean ± SEM. *P* values were calculated using Welch's ANOVA followed by Holm-adjusted Games–Howell post hoc comparisons.

Figure S8.

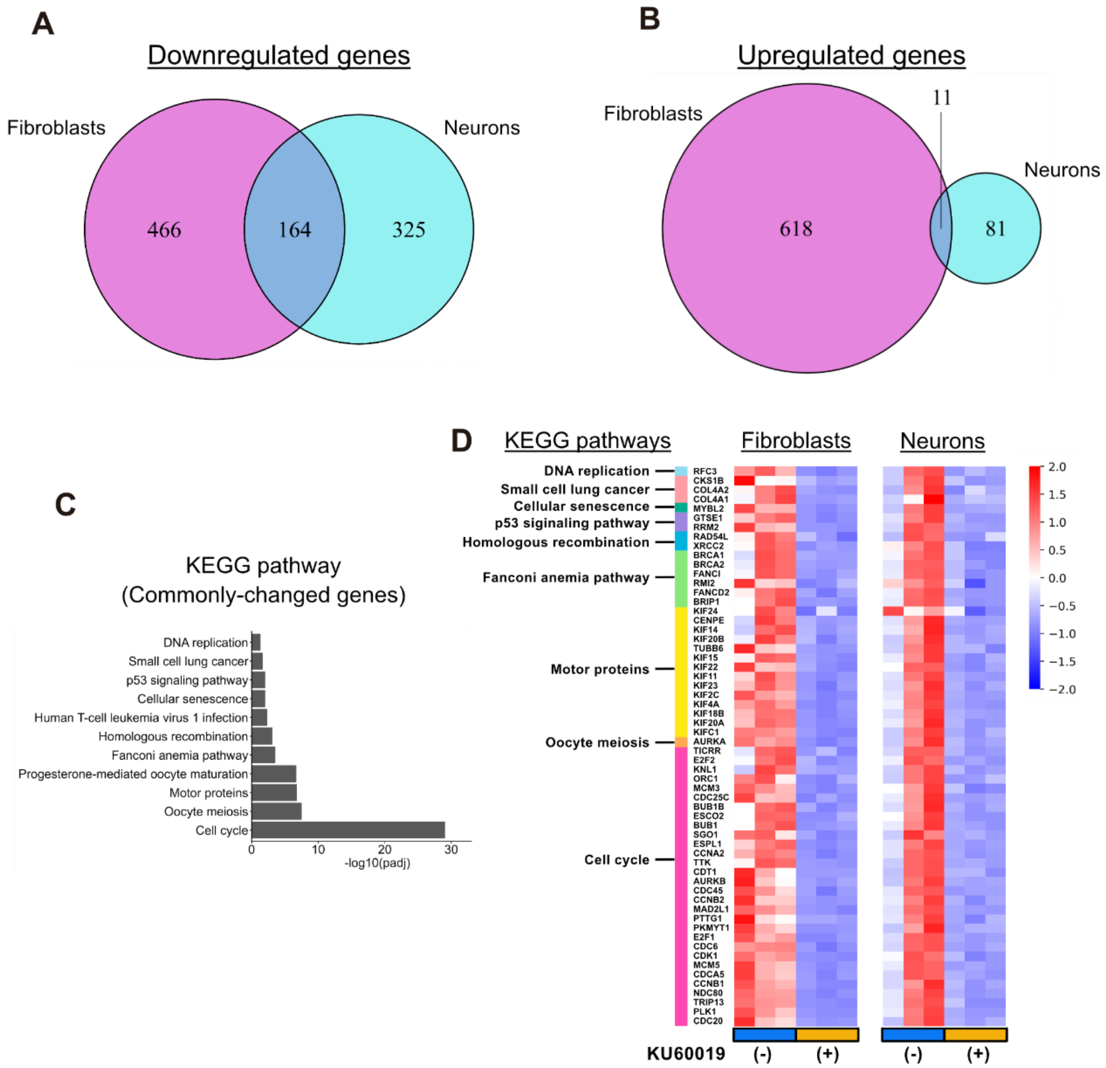

Figure S8. Transcriptomic analysis identifies genes commonly altered by KU60019 in neurons and fibroblasts, related to Figure 4.

(A, B) Venn diagrams showing genes commonly regulated by KU60019 treatment in hiPSC-derived neurons and young fibroblasts. A total of 164 genes were commonly downregulated (A), and 9 genes were commonly upregulated (B).

(C) KEGG pathway enrichment analysis of genes commonly downregulated following KU60019 treatment in hiPSC-derived dopaminergic neurons and young fibroblasts.

(D) Heatmaps showing the expression of genes associated with pathways enriched in the KEGG analysis in hiPSC-derived dopaminergic neurons and young fibroblasts.

Figure S9.

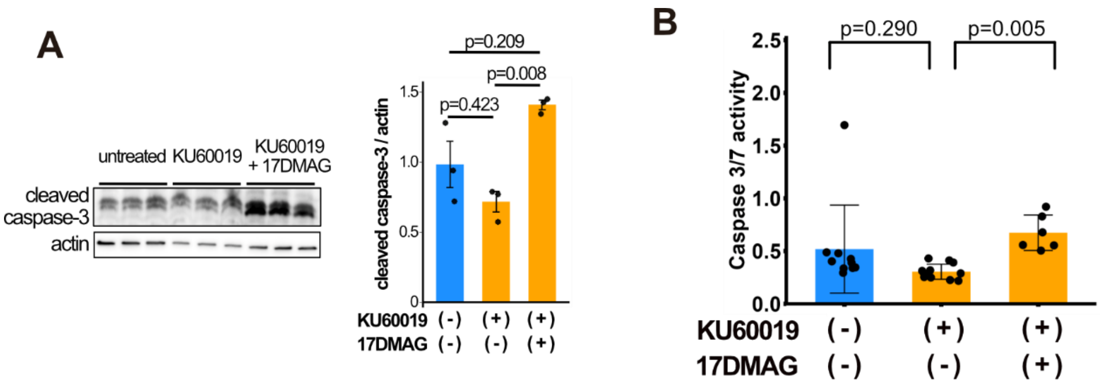

Figure S9. 17DMAG induces apoptosis in KU60019-treated cells, related to Figure 5.

(A) Immunoblot analysis of cleaved caspase-3 protein levels in untreated, KU60019-treated, and KU60019 + 17DMAG–treated neurons. n = 3 independent experiments.

(B) Caspase-3/7 activity in untreated, KU60019-treated, and KU60019 + 17DMAG–treated young fibroblasts. n = 6–12 independent experiments.

Data are shown as mean ± SEM. *P* values were calculated using Welch's ANOVA followed by Holm-adjusted Games–Howell post hoc comparisons.

Figure S10.

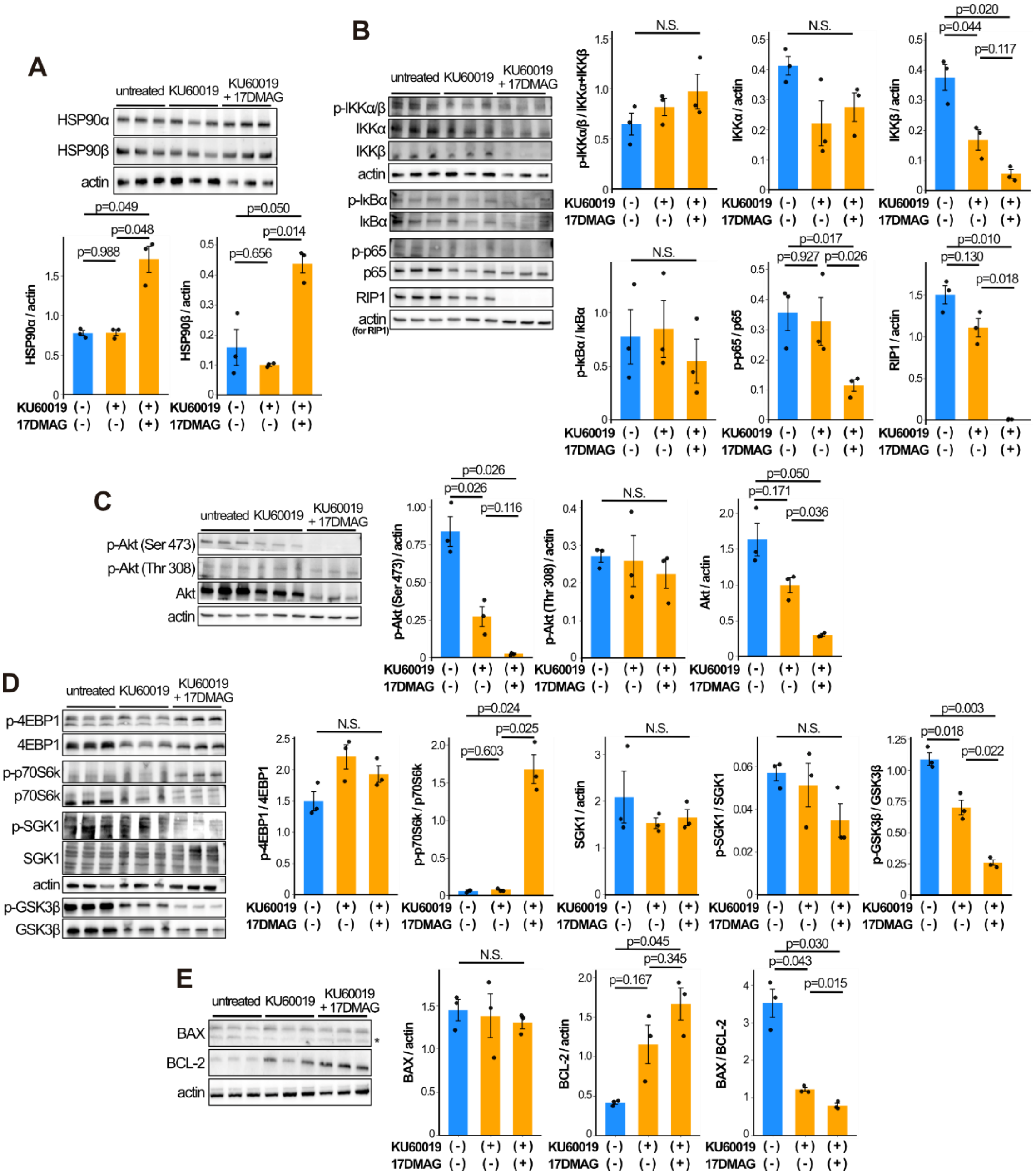

Figure S10. Reproducibility of KU60019-induced signaling alterations across independent hiPSC-derived neuronal lines, related to Figure 6.

(A) Immunoblot analysis of HSP90 protein levels in untreated, KU60019-treated, and KU60019 + 17DMAG–treated hiPSC-derived neurons from an independent neuronal line. n = 3 independent experiments.

(B) Immunoblot analysis of NF-κB signaling components and their phosphorylated forms in untreated, KU60019-treated, and KU60019 + 17DMAG–treated neurons. n = 3 independent experiments.
(C) Immunoblot analysis of Akt and phosphorylated Akt protein levels in untreated, KU60019-treated, and KU60019 + 17DMAG–treated neurons. n = 3 independent experiments.
(D) Immunoblot analysis of Akt-mTOR pathway components and their phosphorylated forms in untreated, KU60019-treated, and KU60019 + 17DMAG–treated neurons. n = 3 independent experiments.
(E) Immunoblot analysis of BAX and BCL-2 protein levels in untreated, KU60019-treated, and KU60019 + 17DMAG–treated neurons. n = 3 independent experiments.
The same actin blot is shown for RIP1 in panel (B) and for the proteins shown in panel (C) because these proteins were detected from the same membrane using the same set of lysates. Data are shown as mean ± SEM. *P* values were calculated using Welch's ANOVA followed by Holm-adjusted Games–Howell post hoc comparisons. N.S., not significant.

Figure S11.

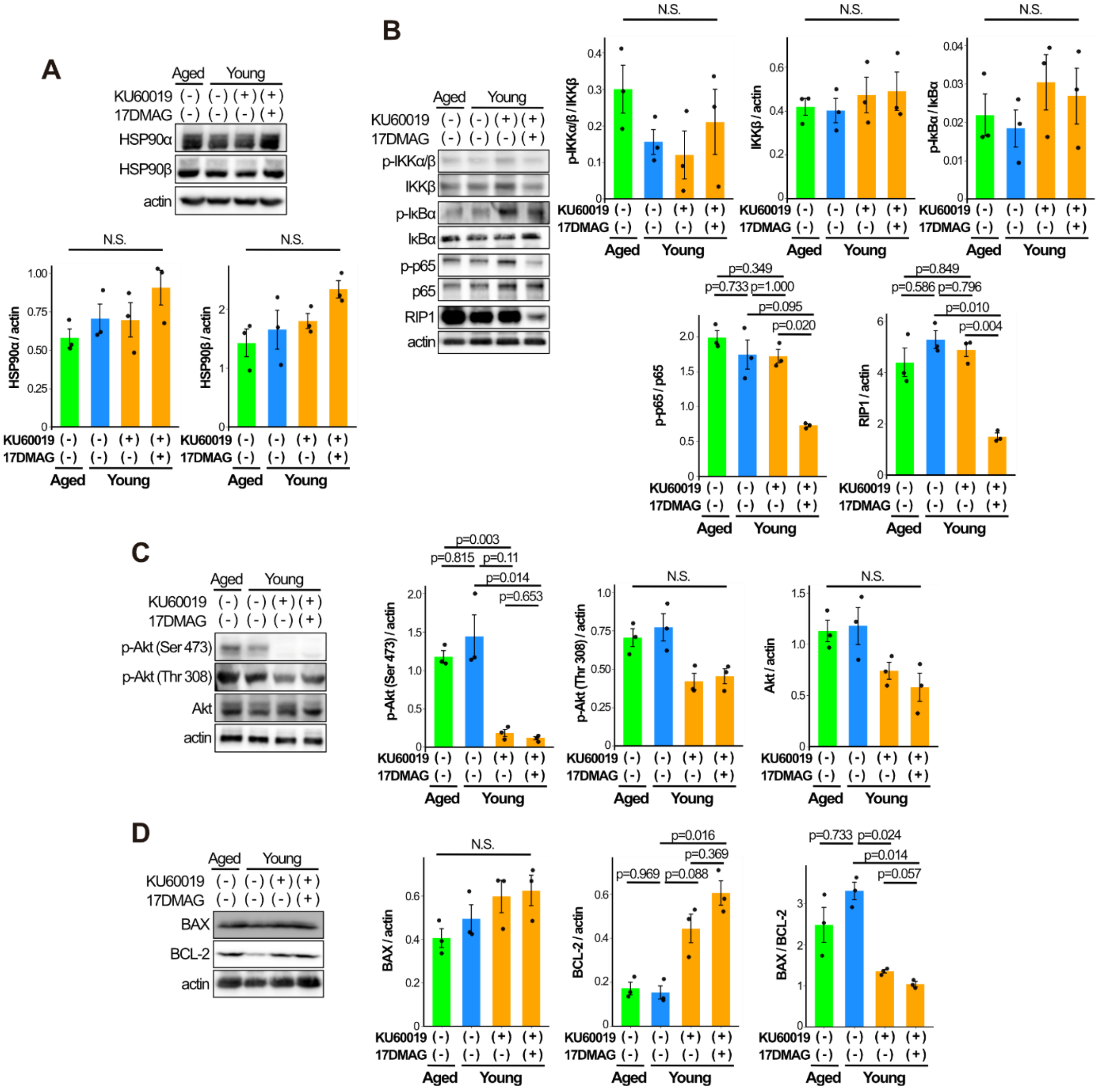

Figure S11. Reproducibility of KU60019-induced signaling alterations in fibroblasts, related to Figure 6.

(A) Immunoblot analysis of HSP90 protein levels in aged fibroblasts and in untreated, KU60019-treated, and KU60019 + 17DMAG–treated young fibroblasts. n = 3 independent experiments.

(B) Immunoblot analysis of NF-κB signaling components and their phosphorylated forms in aged fibroblasts and in untreated, KU60019-treated, and KU60019 + 17DMAG–treated young fibroblasts. n = 3 independent experiments.

(C) Immunoblot analysis of Akt and phosphorylated Akt in aged fibroblasts and in untreated, KU60019-treated, and KU60019 + 17DMAG–treated young fibroblasts. n = 3 independent experiments.

(D) Immunoblot analysis of BAX and BCL-2 protein levels in aged fibroblasts and in untreated, KU60019-treated, and KU60019 + 17DMAG–treated young fibroblasts. n = 3 independent experiments.

Data are shown as mean ± SEM. *P* values were calculated using Welch's ANOVA followed by Holm-adjusted Games–Howell post hoc comparisons. N.S., not significant.

Table S1. List of compounds and corresponding synapsin-GFP fluorescence intensity values from the compound screening, related to Figure 1. (Excel file)

Table S2. List of differentially expressed genes in hiPSC-derived dopaminergic neurons following KU60019 treatment, related to Figure 4 and Figure S8. (Excel file)

Table S3. RNA-seq–based comparison of gene expression in untreated young fibroblasts, KU60019-treated young fibroblasts, and aged fibroblasts, related to Figure 4 and Figure S8. (Excel file)
